# Cost Effectiveness of Adjunctive Neurofeedback vs. Psychotherapy or Pharmacotherapy for Post-Traumatic Stress Disorder

**DOI:** 10.3390/healthcare13192388

**Published:** 2025-09-23

**Authors:** Jeffrey D. Voigt, Aron Tendler, Carl Marci, Linda L. Carpenter

**Affiliations:** 1 Medical Device Consultants of Ridgewood, Ridgewood, NJ 07450, USA; 2 GrayMatters Health, Haifa 3303403, Israel; aron.tendler@gmail.com; 3 Massachusetts General Hospital, 300 Ocean Ave., Revere, MA 02151, USA; cmarci@om1.com; 4 Department of Psychiatry and Human Behavior, Alpert Medical School of Brown University, 345 Blackstone Blvd., Providence, RI 02906, USA; linda_carpenter_md@brown.edu

**Keywords:** neurofeedback, PTSD, cost effectiveness, psychotherapy, pharmacotherapy

## Abstract

**Background:** Neurofeedback shows promise as an adjunctive therapy for post-traumatic stress disorder (PTSD), but its cost effectiveness has not been studied. **Objectives:** To assess the cost and effectiveness of neurofeedback plus other therapies (NF + OT) vs. guideline therapies alone. **Methods:** TreeAge software was used to develop Markov models comparing NF + OT therapy to psychotherapy and pharmacotherapy over 1–3 years. Costs were derived from Medicare rates and literature. Effectiveness was measured using CAPS-5 score reductions converted to quality-adjusted life years (QALYs) using regression analysis. Dropout and relapse rates were derived from systematic reviews and meta-analysis. **Results:** NF + OT resulted in greater improvements in CAPS-5 scores and was less costly than OT. In the base case, NF + OT was less expensive (on average) for years 1–3 by USD 2568−USD 4140 (vs. psychotherapy) and USD 2282−USD 7217 (vs. pharmacotherapy). QALYs improved by 0.04 compared to psychotherapy and 0.24 compared to pharmacotherapy. NF + OT dominated (lower cost, better outcomes) psychotherapy 12% of the time and pharmacotherapy 26.5% of the time in Monte Carlo simulation. Further, Monte Carlo simulation did not demonstrate dominance at any point in time for either pharmacotherapy or psychotherapy over NF + OT. **Conclusions:** Based on lower costs and improved effectiveness, NF + OT should be considered for treating PTSD.

## 1. Introduction

Approximately 5–9% of people exposed to a traumatic event will go on to develop post-traumatic stress disorder (PTSD) [1]. Patients with chronic PTSD experience work and relationship dysfunction and, in some cases, become refractory to evidence-based treatments (EBTs) including cognitive behavior therapy and pharmacotherapy [2]. The 2018 direct health care costs for treating patients with PTSD were estimated at USD 76.1 billion in the United States [3]. Related comorbid conditions (substance abuse, homelessness, and disability) add another USD 35.7 billion [3].

A major limitation of both psychotherapy and pharmacotherapy is high non-adherence rates [4,5]. Non-adherence rates of 33% for pharmacotherapy [4] and 17.2% to 24% for psychotherapy [5,6] may result from patients’ reluctance to reexperience trauma during therapy, perceived lack of response to treatment, the treatment-resistant nature of PTSD, and medication side-effects. These factors can lead to higher costs and worse outcomes.

Neurofeedback (NF) teaches self-regulation of brain functions by providing real-time feedback of brain activity to modify behavior and emotional states. With NF, brain function is commonly captured via electroencephalogram (EEG). NF has been extensively studied in controlled clinical trials, with significant efficacy outcomes demonstrated on multiple measures examined in a recent meta-analysis [7]. PTSD interventions relevant to this analysis demonstrated cost effectiveness over 3-year timeframes anywhere from 37% to 100% at a quality-adjusted life year (QALY) of USD 25,000 [8,9]. A QALY is a unit of measurement that combines a person’s life expectancy with their quality of life over time into a single value. QALYs are part of an incremental cost-effectiveness ratio (ICER) equation [incremental costs over time (aggregated)/incremental QALYs over time (aggregated)] used to guide health care policy and overall cost effectiveness.

Newer NF technologies can assess deeper processes of the brain associated with PTSD symptoms, such as amygdala activity, using functional magnetic resonance imaging (fMRI) [10,11]. By integrating simultaneous EEG recordings with fMRI, and through application of machine learning, researchers have identified a set of coefficients for a targeted network; this is referred to as an EEG–fMRI pattern (EFP). A novel NF system (Prism^(R)^ by GrayMatters Health) has been designed to provide EFP-based feedback to patients, allowing for self-neuromodulation of deeper and more distributed networks. Prism delivers amygdala-derived-EFP NF, and was recently FDA cleared for adjunctive use with psychotherapy or pharmacotherapy in the treatment of PTSD [12] (Supplementary S1). Meta-analyses of randomized controlled studies incorporating EFP-informed NF for treating PTSD undertaken for this current analysis have been shown to have a larger effect size (pre- vs. posttreatment) vs. traditional EEG NF: 1.48; 95% CI (0.14 to 2.83); *p* = 0.02 for EFP-informed NF vs. 0.68; 95% CI (0.2 to 1.15); *p* = 0.006 for traditional NF when examining PTSD outcome measures (Supplementary S2).

It is the aim of the present cost-effectiveness analysis to use Markov modeling to examine NF + other therapies (OTs) vs. psychotherapy or pharmacotherapy on cost effectiveness. OTs are defined as follows: psychotherapy, pharmacotherapy, or a combination of psychotherapy + pharmacotherapy (P + P). While P + P is not an accepted EBT due to a lack of proven clinical benefit [13,14,15,16], NF has been used with P + P, demonstrating positive clinical outcomes in meta-analyses [7]. Markov modeling/analysis is a probabilistic technique predicting a future state of a variable; it uses ranges of values (as seen in clinical practice) selected randomly over simulations to identify that future state [17]. This study is needed, as there are not any cost-effectiveness comparisons of NF (an emerging therapy) to other forms of therapy in treating PTSD.

## 2. Methods

TreeAge Pro Version 2025 R2.0 software (Williamstown, MA, USA) was used to develop Markov models comparing NF + OT to psychotherapy alone and to pharmacotherapy alone. The models evaluated costs and effectiveness over 1 to 3 years, with quarterly cycles (12 total cycles). A 1- to 3-year timeframe was chosen based on available data and associated enrollee turnover in health care insurance programs [18].

Direct medical costs were derived from published Medicare and Veterans Administration (VA) reimbursement rates. Medicare rates were chosen as they most closely resemble actual costs for care [19]. Associated additional costs in treating mild, moderate, and severe PTSD were derived from the literature and, again, mainly reflected costs for care [20,21]. These costs were as of the year 2023. Effectiveness was derived from systematic reviews and meta-analysis of psychotherapy, pharmacotherapy, and NF + OT, which used Clinician-Administered PTSD Scale for DSM-V (CAPS-5) score reductions to define effectiveness [7,22,23]. Search terms used in identifying the systematic reviews and meta-analyses were systematic review AND meta-analysis AND PTSD AND randomized controlled trials AND clinician administered PTSD scale AND psychotherapy OR pharmacotherapy OR neurofeedback for the years 2000-present. These publications (as referenced) were used as the basis for clinical guidelines by specialty societies such as psychiatry and psychology. The control groups used in the systematic reviews and meta-analyses as comparators were either placebo or no treatment (e.g., waiting list) and were accepted by these specialty societies. NF comparators were either sham or no treatment. CAPS-5 score reductions were converted to EuroQol Visual Analogue Scale (EQ-VAS) scores to calculate Quality-Adjusted Life Years (QALYs) using a linear regression model (calculated by the authors) based on findings from a randomized controlled study of 147 patients examining both CAPS-5 and EQ-VAS in evaluating PTSD (see Supplementary S3 for the linear regression model used along with the coefficients) [24]. This regression model demonstrated that improvements in CAPS-5 scores (i.e., lower scores) were associated with improved quality of life (EQ-VAS) (i.e., higher VAS scores)—a finding consistent with prior studies [25,26]. The key assumptions for clinical improvement in PTSD symptoms were derived from CAPS-5 scores. Improvements in CAPS-5 for psychotherapy, pharmacotherapy, and NT + OT are identified in the Results Section 3.1 and Section 3.2 and, again, were derived from systematic reviews and meta-analyses. CAPS-5 were converted to EQ-VAS using linear regression analysis from data obtained on CAP-5 and EQ-VAS in the peer-reviewed literature [24]. CAPS-5 was chosen as the outcome of interest, as it is a clinician-reported outcome and not self-reported as is PCL-5.

The key cost assumptions used In the model are found in Supplementary S4 and S5 and include the following (Table 1):

Patients moved between different health states quarterly over 3 years based on treatment response, starting from an initial clinical state (e.g., “severe” PTSD (CAPS-5 score 60–80), “moderate” PTSD (40–59), “mild” PTSD (20–39)); relapse (14% for psychotherapy [28]; 17.4 ± 5% for pharmacotherapy [29]; and 14% for NF + OT [7]); re-treatment (20–80% range for all therapies); dropout (33% for pharmacotherapy [4], 17.2–24% for psychotherapy [5,6]; and 13.2% NF + OT [7]); and death (See Figure S1 for health states of each treatment). These health states were affected by dropouts and relapses, which resulted in a patient reverting to their original condition. As referenced, relapse and dropout rates were derived from systematic reviews and meta-analyses. Patients cycled through various states—e.g., from severe PTSD to moderate to mild and asymptomatic PTSD based on therapy success (i.e., CAPS-5 improvement)—and continued therapy until they were asymptomatic or dropped out or relapsed (based on probabilities derived from systematic reviews and meta-analyses). Gamma distributions were used to model positive values that change over time (e.g., increasing costs). Beta distributions were used to model probabilities or proportions that fall between 0 and 1 (e.g., probability of relapse, probability of dropout). Patients continued to be treated (no matter the treatment method) as they improved on the PTSD continuum over the 3 years until asymptomatic or relapsed or dropped out. If a patient relapsed, they were retreated with the same therapy but entered back into the model at their original PTSD state. Patients who dropped out were assumed to remain at the health state (mild, moderate, or severe PTSD) they dropped out at. If a patient ended up in the “asymptomatic health state” based on cycling through the model, they remained in that state for the 3-year period. Tornado plots, 1-way sensitivity of variables which affected costs, and incremental cost-effectiveness scatter diagrams (10,000 Monte Carlo simulations) were generated at 3 years. Costs and utilities were discounted at 3% annually [30]. A WTP threshold of USD 0/QALY was used in this analysis.

Risks of death were modeled based on the PTSD health state the patient was in during that cycle (mild, moderate, severe) [20]. Relapse rates were derived from a systematic review and meta-analysis and were assumed to be the same for all therapies evaluated [28]. Side effects were noted and were reflected in the cost calculations over time, most specifically for medications [31].

Patients who entered the model were in one of three PTSD states, 40–45 years old, and majority female, consistent with the published demographics of PTSD [32]. Calculations of cost and effectiveness with the variables used for each health state are found in Supplementary S6.

The consolidated health economic evaluation reporting standards (CHEERS) checklist was utilized (Supplementary S7) [33]. For further clarification on data extracted from systematic reviews and meta-analyses that were used in the Markov model, see Supplementary S8.

Tornado plots were generated (using one way sensitivity) to identify those variables (varied plus or minus their value) that affected the cost and effectiveness outcomes.

The perspective of this analysis is from the US health care system and reflects direct costs for care only.

Lastly, based on the higher percentage of military participants in the psychotherapy group, CAPS-5 scores (which demonstrate a lower improvement in military patients) in the psychotherapy group were normalized (Supplementary S9) to align with the NF + OT group (which had a lower percentage of military patients in the systematic review).

The objectives of this analysis were to compare therapies on cost and effectiveness over a 3-year period (NF + OT, pharmacotherapy, and psychotherapy) in the treatment of patients (as described above) in various states of PTSD (mild, moderate, and severe).

## 3. Results

### 3.1. NF + OT vs. Psychotherapy

**Figure 1:**
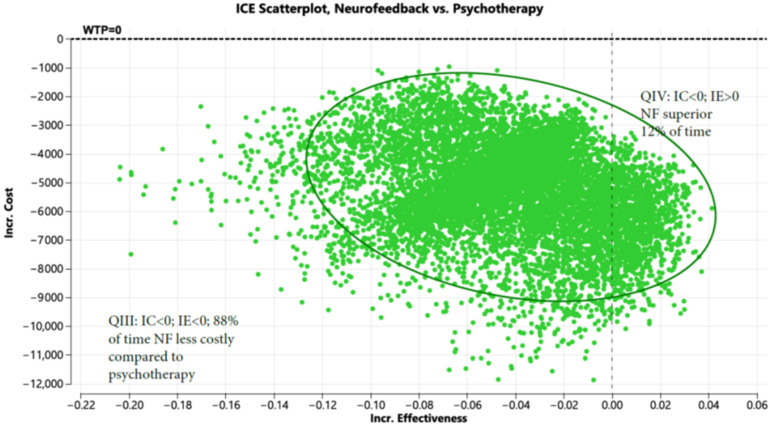
Incremental cost-effectiveness scatterplot of neurofeedback vs. psychotherapy (Note: Green represents iteration which favor NF based on cost).

The Markov model for psychotherapy can be found in Figure S2. Supplementary S4 shows the variables and distributions used in the Markov Model. As shown in prior systematic reviews and meta-analyses, compared to psychotherapy alone, NF + OT is associated with relatively lower dropout rates (13.2% [7] vs. 17.2–24%) [5,6] and greater improvements in CAPS-5 scores (7.01; 95% CI: 1.36 to 12.66 [7]; vs. 5.98 ± 4.94 [22]). Compared to psychotherapy alone, NF + OT saved USD 2568 in year 1, USD 3062 in year 2, and USD 4140 in year 3. It also improved QALYs by 0.04 over the three-year period (Table S1). Shown in Figure 1 is the incremental cost-effectiveness scatterplot generated, with NF + OT dominating (lower cost and improved outcomes) psychotherapy 12% of the time and was less costly 88% of the time.

Figure 1 displays the incremental cost (Y axis) and incremental effectiveness (X axis) of neurofeedback (NF) compared to psychotherapy. The scatterplot demonstrates that NF is less costly than psychotherapy 88 percent of the time and both less costly with improved effectiveness 12 percent of the time when evaluated in a Markov model (10,000 iterations).

Shown in Figure S3 is the tornado diagram with associated threshold values (all else being equal), which would result in one therapy being less expensive than the other. Table 2 shows the threshold values for variables in this model.

### 3.2. NF + OT vs. Pharmacotherapy

**Figure 2:**
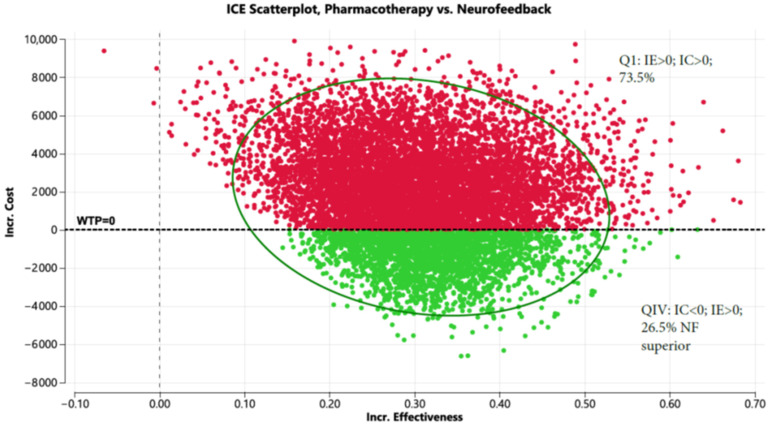
Incremental cost-effectiveness scatterplot comparing pharmacotherapy to neurofeedback (Red represents pharmacotherapy and green represents NF+OT).

The Markov model for pharmacotherapy can be found in Figure S6. Shown in Supplementary S5 are the variables and distributions used in the Markov Model. Based on the fact that, relative to PTSD pharmacotherapy, treatment with NF + OT has lower dropout rates (13.2% vs. 33%) [5,7], and greater improvements in CAPS-5 scores (7.01; 95% CI: 1.36 to 12.66 [7] vs. 6.64; 95% CI: 4.16 to 9.11 [23]), NF + OT was less expensive by USD 2282, USD 4735, and USD 7217 in years 1, 2, and 3, respectively (Table S1) and improved QALYs by 0.24 over the three-year period. Figure 2 shows the incremental cost-effectiveness scatterplot; NF + OT dominated pharmacotherapy 26.5% of the time and is less costly than pharmacotherapy 73.5 percent of the time.

Figure 2 displays the incremental cost (Y axis) and incremental effectiveness (X axis) of pharmacotherapy compared to neurofeedback. The scatterplot demonstrates that NF is less costly than pharmacotherapy 73.5 percent of the time and both less costly and more effective 26.5 percent of the time when evaluated in a Markov model (10,000 iterations).

Shown in Figure S7 is the tornado diagram with associated threshold values for cost. Table 3 shows threshold values for variables in this model.

A separate analysis was run to evaluate the cost effectiveness of the novel NF technology (Prism) delivered as an adjunct therapy to OT (Prism + OT). Supplementary S1 describes the fMRI-informed amygdala-derived EFP and the Prism NF session protocol. A recently published paper found that 3 months after an 8-week course of treatment, CAPS-5 scores were reduced by an average of 13.2 points (95% CI: 10 to 16.4) [34] and continued to improve posttreatment. Using these values in both Markov models, it was found that Prism + OT had cost savings vs. psychotherapy of USD 6766, USD 7764, and USD 8267, aggregated for years 1, 2, and 3, respectively (Table S2), and improved QALYs of 0.08 over 3 years (2.77 vs. 2.69). Additionally, when evaluating Prism + OT vs. pharmacotherapy, it was found that Prism + OT dominated (less expensive, with improved QALYs) pharmacotherapy 86% of the time (Figure S10), with cost savings of USD 6480, USD 9437, and USD 12,449 for years 1, 2, and 3, respectively (Table S2).

## 4. Discussion

The current cost-effectiveness analysis demonstrates that adding NF to standard treatments produces cost savings and improved outcomes. Two main reasons for this relate to better outcomes with NF + OT and lower dropout rates with NF + OT (13.2%) [7] relative to those reported for psychotherapy or pharmacotherapy, which were in the range of 17–33% [5,6]. Patients who drop out of therapy likely return to their previous condition, which is more expensive to treat than continuing therapy with ongoing improvement [20]. Possible reasons for lower dropout rates in NF trials with OT include the focus on trauma symptom reduction by relaxation and stress reduction with NF rather than on trauma-focused treatment with exposure (which characterizes evidence-based psychotherapies for PTSD [5,6]); the lack of side effects associated with NF [7]; and the ability of NF to regulate emotions across other comorbid conditions such as anxiety, depression, bipolar disorder, and substance abuse that commonly present in patients with PTSD (see below). These possible reasons merit exploration in future studies on NF + OT.

Other factors may have influenced dropout rates and outcomes. No specific criteria excluded PTSD patients with active psychiatric comorbidities in any of the systematic reviews and meta-analyses we referenced, and such comorbidities impact dropout/retention [35,36,37]. Comorbidities as a variable were not assessed in the model. Treatment duration with combination NF + psychotherapy (where psychotherapy was used as the OT) was the same as the treatment duration for psychotherapy alone. While those in the NF + OT group exhibited a range of severity (as measured by CAPS-5) and more severe PTSD at treatment entry is associated with inferior response [34,38], details about range of symptom severity were lacking in the other systematic reviews and meta-analyses we utilized. The age of patients was similar across all systematic reviews and meta-analyses. There was a preponderance of females (68%) and civilians (87%) in the NF + OT group [7], with similar demographics seen in the pharmacotherapy systematic review and meta-analysis used in this analysis [4].

Female and civilian populations show better clinical outcomes at follow-up compared to male and military populations. The low male participation rate is common in civilian PTSD studies and stands in stark contrast to the close to 100% male participation characteristic of military studies [5]. The effects of gender and population type on PTSD symptomatology still need to be more fully understood [6]. PTSD associated with both combat/military and physical assault traumas benefits less from EBT than PTSD arising after other types of trauma such as accidents, illness, and disasters [39]. As mentioned in the Methods section, based on the higher percentage of military participants in the psychotherapy group (i.e., double that of the NF + OT group), we normalized CAPS-5 scores in the psychotherapy group to align with the NF + OT group; this, in turn, adjusted upward the CAPS-5 average reduction from 5.98 to 6.25 in the psychotherapy group (Supplementary S9). In a separate analysis not reported in the Results section, this adjustment step demonstrated that the CAPS-5 reduction with psychotherapy would need to be at least 13 points or more to consider changing therapies (see Figure S9 for methodology used).

This analysis builds on a prior randomized controlled trial that found NF to be cost effective at a willingness-to-pay threshold of USD 25,000/QALY [9]. The differences between the current study and Leem et al. [9] are as follows: First, Leem is a small study of 22 patients. The current analysis utilized meta-analyses to examine similar outcomes of much larger numbers of patients. Second, the Leem et al. [9] analysis found significantly higher QoL improvements with NF vs. control. The current analysis on QoL was more tempered, likely owing to its examination of relapse and dropouts from each therapy, which was not included in Leem et al. [9]. Third, Leem et al. [9] examined costs from a societal perspective (including loss of productivity and transportation costs); the current analysis only examined the direct costs for care.

Why does NF + OT outperform psychotherapy or pharmacotherapy alone? One explanation is that PTSD patients commonly present with multiple comorbidities. Brady and colleagues found 59% of men and 44% of women with PTSD met diagnostic criteria for three or more other psychiatric disorders, including depression, anxiety disorders, and substance use disorders [40]. These commonly occurring comorbid conditions and the heterogeneity of clinical presentation in PTSD may limit the overall efficacy of treatments designed to address a limited set of symptoms [41]. Transdiagnostic clinical interventions apply similar treatment principles to address a symptom that is common across multiple different mental disorders. This is in contrast to tailoring a treatment protocol addressing one specific diagnosis [41]. Emotion regulation therapy (using NF) is an example of a transdiagnostic intervention that can reduce symptoms across multiple comorbid conditions. When used as an adjunct to other evidence-based treatments, enhanced emotional regulation is associated with significant improvement of symptoms of comorbid disorders [42]. A more detailed rationale on all the benefits from integrating amygdala-EFP-NF with psychotherapy was recently published, along with a detailed case report on one of the participants from the clinical trials [43]. A more recent publication on the use of amygdala EFP neurofeedback and its effects on PTSD symptom clusters (such as arousal/reactivity, avoidance, and mood) demonstrates EFP neurofeedback’s effect on emotional regulation (ER) via the amydala (a key structure for ER and PTSD pathophysiology). Thus, patients have a better ability to monitor, identify, and modify emotional responses seen in PTSD over the longer term [44].

What is notable regarding the above analysis is that NF + OT improves QALYs by 0.24 (on a scale from 0 to 1). This improvement, when converted to CAPS-5, demonstrates that NF + OT can improve a PTSD patient from severe to mild or from moderate to asymptomatic when evaluated on the CAPS-5 scale. Again, this is due to a lower dropout rate and improved CAPS-5 over time vs. pharmacotherapy.

This analysis has several limitations. We examined costs and outcomes quarterly over 3 years but did not assess lifetime costs and outcomes. Longer term data outside of 3 years could not be identified in the literature for any of the therapies. Additionally, the systematic reviews and meta-analysis that provided our model data came from different studies. While the PTSD patients evaluated based on therapeutic intervention were of similar age and gender, there were differences in duration of PTSD illness and other comorbidities. This may have affected the findings. The linear regression equation used in converting CAPS-5 to EQ-5D scores came from a randomized controlled study [24]. The findings of this study, namely that there is a negative correlation between PTSD health instruments such as CAPS-5 and EQ-5D (in other words, as CAPS-5 scores decrease, EQ-5D scores increase or a better quality of life is realized), have been validated in other studies [24,45]. The comparators to the therapies of NF, psychotherapy, and pharmacotherapy included sham, placebo, and waiting list. The control arm is a combination of sham NF and waitlist from psychotherapy trials and placebo arms from pharmacotherapy trials. While this is not ideal, it is consistently carried out in the meta-analytic literature.

## 5. Conclusions

In conclusion, due to improved efficacy and lower dropout rates compared to pharmacotherapy or psychotherapy alone, NF + OT results in lower costs in the vast majority of cases vs. psychotherapy and pharmacotherapy. As well, outcomes are improved with NF + OT the vast majority of time vs. pharmacotherapy. A specific form of NF, using an amygdala-based biomarker (Prism) may provide even better outcomes but requires further study.

## Figures and Tables

**Table 1:** Key cost assumptions.

Variable	Cost	Assumed # Sessions	Total Cost
Cost treating mild PTSD exclusive of therapy quarterly [20]	USD 3450	N/A	USD 3450
Cost treating moderate PTSD exclusive of therapy quarterly [20]	USD 4900	N/A	USD 4900
Cost treating severe PTSD exclusive of therapy quarterly [20]	USD 5880	N/A	USD 5880
Cost psychotherapy *	USD 147	13.5	USD 1985
Cost pharmacotherapy yearly [27]	USD 1415	N/A	USD 1415
Cost NF + psychotherapy *	USD 140	13.5	USD 1890

* Medicare reimbursement rates for CPT code 90876 (NF + 45 min psychotherapy sessions) plus 90837 (psychotherapy; 60 min). The numbers in Table 1 represent 2023 costs associated with treating PTSD, complications, and other non-PTSD treatment care associated with it. # means abbreviation for “Number”. The numbers in Table 1 represent 2023 costs associated with treating PTSD, complications, and other non-treatment care associated with it.

**Table 2:** One-way sensitivity analysis NF + OT vs. psychotherapy.

Variable	Value Used in Model	Value at Which Alternative Therapy (Psychotherapy) Becomes the Less Expensive Alternative	Figure
Probability of dropout from psychotherapy	17.2–24%	<9.9%	Figure S4
Probability of dropout from NF + OT	13.2%	>27%	Figure S5

**Table 3:** One-way sensitivity analysis NF + OT vs. pharmacotherapy.

Variable	Value Used in Model	Value at Which Alternative Therapy (Pharmacotherapy) Becomes the Less Expensive Alternative	Figure
Dropouts from pharmacotherapy (percent)	33%	<9.8%	Figure S8
Dropouts from NF + OT	13.2%	>37.6%	Figure S9

## Data Availability

All supporting data can be found in the Supplementary Materials.

## Supplementary Materials

The following supporting information can be downloaded at: https://www.mdpi.com/article/10.3390/healthcare13192388/s1, Supplementary materials published online include the following: Supplementary items and descriptions; Supplementary S1. Description Prism technology; Supplementary S2. Effect sizes EFP informed NF vs. traditional NF; Supplementary S3. Regression equation CAPS-5 and EQ-VAS; Supplementary S4. Variables and distributions NF+OT vs. psychotherapy; Supplementary S5. Variables and distributions NF+OT vs. pharmacotherapy; Supplementary S6. Calculations used in each health state; Supplementary S7. CHEERS checklist; Supplementary S8. Methodology of systematic reviews and meta-analyses used in manuscript; Supplementary S9. Adjustment of CAPS-5 scores; Figure S1. State transition diagram; Figure S2. Decision tree NF+OT vs. psychotherapy; Figure S3. Tornado diagram: ICER NF+OT vs. psychotherapy; Figure S4. One way sensitivity psychotherapy—probability dropout; Figure S5. One way sensitivity NF+OT—probability dropout; Figure S6. Decision tree NF+OT vs. pharmacotherapy; Figure S7. Tornado diagram: NF+OT vs. pharmacotherapy; Figure S8. One way sensitivity pharmacotherapy-probability dropout; Figure S9. One way sensitivity NF+OT dropout rate; Figure S10. ICE scatterplot Prism vs. pharmacotherapy; Figure S11. One way sensitivity CAPS reduction for psychotherapy; Table S1. Neurofeedback+OT vs. Pharmacotherapy and psychotherapy; costs and savings; Table S2. Prism+OT vs. pharmacotherapy and psychotherapy; costs and savings; Table S3. Summary outputs by stage and state NF vs. psychotherapy; Table S4. Summary outputs by stage and state NF vs. pharmacotherapy.
