# Supplementary material for: Cost Effectiveness of Adjunctive Neurofeedback vs. Psychotherapy or Pharmacotherapy for Post-Traumatic Stress Disorder
Source: Healthcare (Basel). 2025 Sep 23;13(19):2388. doi: 10.3390/healthcare13192388 (PMC12524126; doi:10.3390/healthcare13192388)
Supplement: Supplementary file 1 [file healthcare-13-02388-s001.zip › healthcare-3842148-supplementary.pdf]

Supplementary item	Description	Page number
Supplementary S1	Description Prism technology	2
Supplementary S2	Effect sizes EFP informed NF vs. traditional NF	3
Supplementary S3	Regression equation CAPS-5 and EQ-VAS	4
Supplementary S4	Variables and distributions NF+OT vs. psychotherapy	5-11
Supplementary S5	Variables and distributions NF+OT vs. pharmacotherapy	12-18
Supplementary S6	Calculations used in each health state	19-44
Supplementary S7	CHEERS checklist	45-46
Supplementary S8	Methodology of systematic reviews and meta-analyses used in manuscript	47-48
Supplementary S9	Adjustment of CAPS-5 scores	49
Figure S1	State transition diagram	50
Figure S2	Decision tree NF+OT vs. psychotherapy	51
Figure S3	Tornado diagram ICER NF+OT vs. psychotherapy	52
Figure S4	One way sensitivity psychotherapy - probability dropout	53
Figure S5	One way sensitivity NF+OT - probability dropout	54
Figure S6	Decision tree NF+OT vs. pharmacotherapy	55
Figure S7	Tornado diagram NF+OT vs. pharmacotherapy	56
Figure S8	One way sensitivity pharmacotherapy- probability dropout	57
Figure S9	One way sensitivity NF+OT dropout rate	58
Figure S10	ICE scatterplot Prism vs. pharmacotherapy	59
Figure S11	One way sensitivity CAPS reduction for psychotherapy	60
Table S1	Neurofeedback+OT vs. Pharmacotherapy and psychotherapy; costs and savings	61
Table S2	Prism+OT vs. pharmacotherapy and psychotherapy; costs and savings	61
Table S3	Summary outputs by stage and state NF vs. psychotherapy	62-72
Table S4	Summary outputs by stage and state NF vs. pharmacotherapy	73-83

Supplementary S1 Prism:

Prism's technology integrates simultaneous EEG and fMRI (deeper brain) recordings designated amygdala-derived-EEG-fMRI-Pattern (EFP). By utilizing machine learning for predicting fMRI activity in specific brain regions such as the amygdala from simultaneously acquired EEG data, a set of coefficients, designated Electronic Finger Print (EFP)–EEG-fMRI- were derived[1]. This EFP enables an fMRI-informed EEG signal and provides feedback to the patient. By using the Amygdala-derived-EFP in a NF system, the effect of NF training on a patient's response to non-traumatic stimuli can be measured[2].

Training using Prism technology:

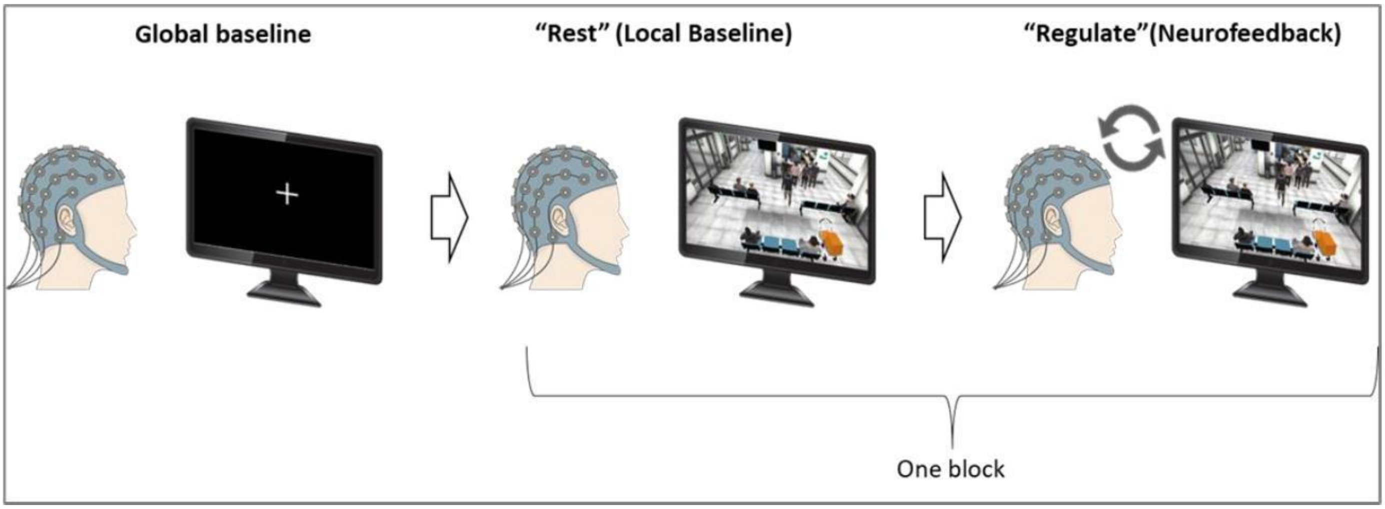

The activities related to training sessions are as follows:

For baseline:
1.1.1 The subject is seated in front of a monitor screen.
1.1.2 Active and reference electrodes are placed, while a ground electrode is clipped onto the subject's earlobe.
1.1.3 The signal quality is verified using an indicator in the Prism software
1.1.4 Before the training starts subjects are asked to limit their movements as much as possible throughout the session (movement however is allowed in between neurofeedback blocks)
1.2.1 Global baseline "rest" recoding, during which the subject is presented with a black screen with a crosshair in its middle. During this part of training, the subject looks at the crosshair but is not given any other specific task.

For regulate:
1.2.2 Neurofeedback cycles, are comprised of (rest and regulate):

> 1.2.3 "Rest" during which the subject is presented with the animated audio-visual interface, which is not yet responsive to the subject's EEG signal. The subject is asked to watch the interface but not attempt to control the signal.
> 1.2.4 "Regulate", i.e. neurofeedback, during which the subject's AmgyEFP signal regulates the level of activity in the animated audio-visual interface, allowing the patient to learn to control this signal.
> 1.2.5 After the "Regulate" phase and prior to the start of the following cycle, there is a few seconds' break, during which the subject may move, stretch etc., prior to the beginning of the subsequent neurofeedback cycle.

---

[1] Keynan JN, Meir-Hasson Y, Gilam G, et al. 2016. Limbic Activity Modulation Guided by Functional Magnetic Resonance Imaging–Inspired Electroencephalography Improves Implicit Emotion Regulation. Biological Psychiatry;*80*(6): 490–496. https://doi.org/10.1016/j.biopsych.2015.12.024

[2] Madhusoodanan J. Better brain training for treating psychological conditions. Nature. 2021. *doi: https://doi.org/10.1038/d41586-021-01664-x.*

Supplementary S2 NF CAPS-5 improvement pre-post 10-15 sessions of EFP informed NF

	Experimental			Control				Std. Mean Difference
Study or Subgroup	Mean	SD	Total	Mean	SD	Total	Weight	IV, Fixed, 95% CI
Bell 2019	28.1	19	12	18.64	16.9	11	32.9%	0.51 [-0.33, 1.34]
Leem 2021	9.9	9.84	10	1.9	7.39	9	25.2%	0.87 [-0.08, 1.82]
Yeganeh 2016	7.7	8.5	15	1.2	9.7	15	41.8%	0.69 [-0.05, 1.43]
**Total (95% CI)**			**37**			**35**	**100.0%**	**0.68 [0.20, 1.15]**

Heterogeneity: Chi² = 0.32, df = 2 (P = 0.85); I² = 0%
Test for overall effect: Z = 2.77 (P = 0.006)

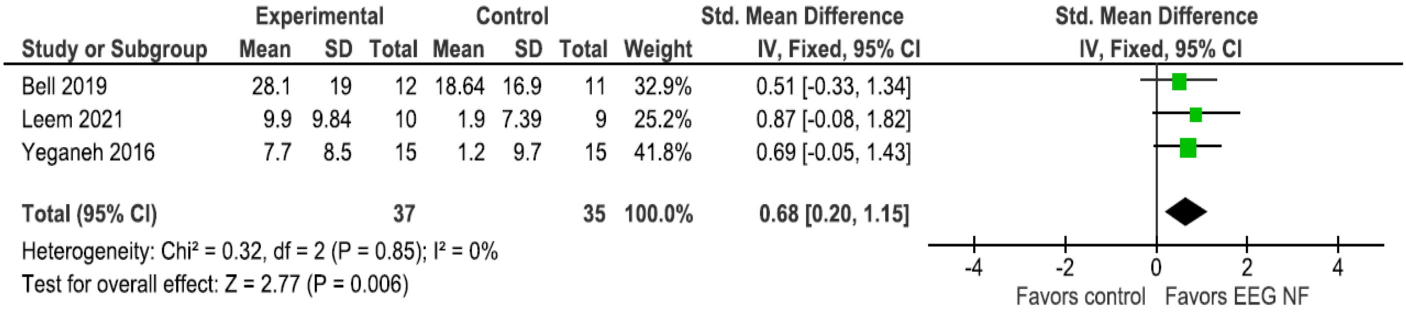

Supplementary S2 - EFP informed NF - CAPS-5 improvement pre-post 10-15 sessions of EFP informed NF

	EFP informed NF			Control				Std. Mean Difference
Study or Subgroup	Mean	SD	Total	Mean	SD	Total	Weight	IV, Random, 95% CI
Fine 2023	8.86	15.4	39	3.28	16	14	36.1%	0.35 (-0.26, 0.97]
Fruchtman-Steinbok(2) 2021 Neu NF	7.23	3.8	13		3.8	13	33.3%	1.59 (0.69, 2.49]
Fruchtman-Steinbok 2021 TR-NF	11.91	4	12		3.8	13	30.6%	2.71 [1.57, 3.84]
**Total (95% CI)**			**64**			**40**	**100.0%**	**1.48 [0.14, 2.83]**

Heterogeneity: Tau'= 1.21; Chi'= 14.51, df = 2 (P = 0.0007); I'= 86%
Test for overall effect Z = 2.16 (P = 0.03)

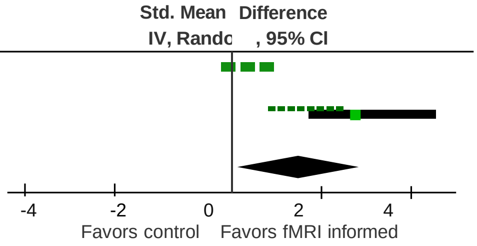

Supplementary S3

EQ VAS correlation to CAPS

Source: Dams J, et al. Psych Quarter 2021;92:459-471.

EQ-VAS (Y)	CAPS (X)	CAPS defintion PTSD
0.95	20.11	asymptomatic
0.9	28.97	mild
0.85	36.5	mild
0.8	46.69	moderate
0.75	55.55	moderate
0.71	65	severe
0.65	73.27	severe
0.6	82.13	extreme
0.53	93.5	extreme

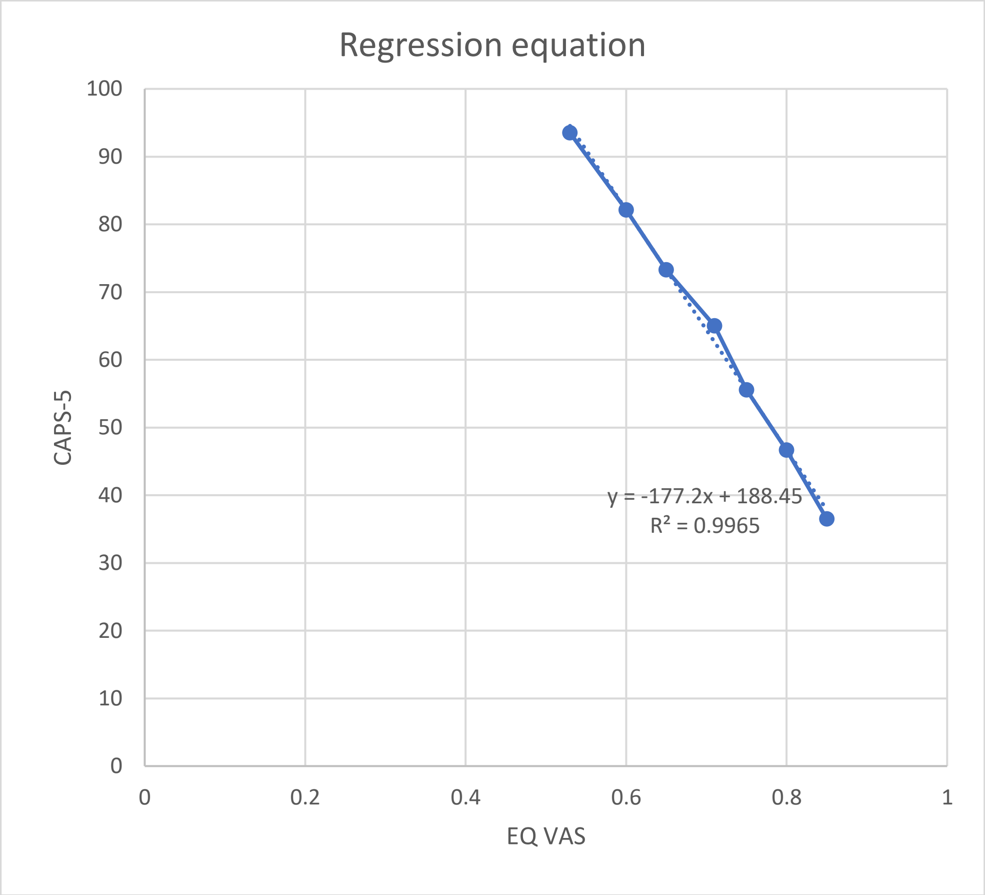

Supplementary S4 – variables NF+OT vs. psychotherapy

Name	Description	Formula	Value	Low	High	Comment
Asymptomatic_PTSD_QoL_quarterly	Quality of life patient who is asymptomatic	0.95/4	0.2375	0	0.225	Source: Dams J, et al. Reliability, validity and responsiveness of the EQ-5D-5L in assessing and valuing health status in adolescents and young adults with posttraumatic stress disorder: a randomized controlled trial. Pysch Quart. 2021;92:459-471.
CPT_90837	CPT payment for psychotherapy - 1 hour	147	147	0	150	CPT code Medicare payment for one hour psychotherapy 2023
CPT_90876	CPT payment for NF + psychotherapy	Cost_NF_plus_psychotherapy	140	0	1000	Medicare 2023 estimated rate on fee schedule
CPT_90901	Reimbursement rate CPT 90901 Neurofeedback	41.43	41.43	0	300	CMS 2023 payment schedule
CPT_90901_booster	NF booster sessions	41.43	41.43	0	300	Additional NF booster session every quarter to maintain status in mild/asymptomatic patients
CPT_99214	CPT 99214 E&M code - established patient	90.82	90.82	0	90.82	CPT code payment for an established patient E&M Medicare 2023
Incidence_death_mid_40s	Incidence of death for a person in their mid 40s	0.0042	0.0042	0	0.0042	https://www.cdc.gov/nchs/data/dvs/MortFinal2007_Worktable23r.pdf
Mild_PTSD_Quality_Life_quarterly	QoL patient with mild PTSD	Mild_PTSD_QoL/4	0.235	0	0.21875	
Moderate_PTSD_Quality_life_quarterly	Moderate PTSD quality of life	Moderate_PTSD_QoL/4	0.19375	0	0.19375	
Number_NF_sessions_treatment	Number of neurofeedback sessions for PTSD treatment	Number_treatment_sessions_NF	2	0	100	
Number_psychotherapy_sessions_treatment	Number psychotherapy sessions in a treatment regimen	Number_treatment_sessions_psychotherapy	11.667	0	20	

Name	Description	Formula	Value	Low	High	Comment
Per_CAPS_reduction_QOL_improvement	Per CAPS unit reduction - associated QOL improvement	0.0456	0.0456	0	0.5	Source: Dams J, et al. Reliability, validity and responsiveness of the EQ-5D-5L in assessing and valuing health status in adolescents and young adults with posttraumatic stress disorder: a randomized controlled trial. Pysch Quart. 2021;92:459-471.
Percent_NF	Percent of patients receiving NF only	0.19	0.19	0	0.99	source: Systematic review and meta-analysis NF RCTs
Percent_NF_meds	Percent of patients receiving NF plus medication	0.23	0.23	0	0.99	**source: Systematic review and meta-analysis NF RCTs**
Percent_NF_meds_psych	Percent of patients receiving NF plus medications plus psychotherapy	0.27	0.27	0	0.27	Source: Systematic review and meta-analysis NF RCTs
Percent_NF_psychotherapy	Percent of patients receiving NF plus psychotherapy	0.31	0.31	0	0.99	source: Systematic review and meta-analysis NF RCTs
Percent_relapse_treatment_PTSD		Prob_treated_PTSD_relapse	0.667	0	0.8	
Prob_dropout_NF_psychotherapy	Probability of dropout of a patient under neurofeeedback and psychotherapy	0.132	0.132	0	0.9	Voigt JD et al. 2024
Prob_dropout_psychotherapy	Probability of dropout from psychotherapy	Probability_dropout_psychotherapy	0.2075	0	0.9	
Prob_relapse_therapy	Probability of relapse from psychotherapy	Probability_relapse_any_therapy	0.0325	0	0.6	
Probability_mild_PTSD	Probability of mild PTSD	0.302	0.302	0	0.302	Kessler RC, et al. Prevalence, severity, and co-morbidity of twelve-month DSM-IV disorders in the national comorbidity survey replication (NCS-R). Arch Gen Psych 2005;62(6):617-627.

Name	Description	Formula	Value	Low	High	Comment
Probability_moderate_PTSD	Probability of moderate PTSD	0.332	0.332	0	0.332	Kessler RC, et al. Prevalence, severity, and co-morbidity of twelve-month DSM-IV disorders in the national comorbidity survey replication (NCS-R). Arch Gen Psych 2005;62(6):617-627.
Probability_side_effects_pharmacotherapy	Probability side effects from pharmacotherapy	0.091	0.091	0	0.091	source: Anderson HD, Pace WD, Libby AM, West DR, Valuck RJ. Rates of 5 common antidepressant side effects among new adult and adolescent cases of depression: a retrospective US claims study. Clin Ther. 2012; 34(1):113–23. https://doi.org/10.1016/j.clinthera.2011.11.024 PMID: 22177545
QoL_improvement_NF_plus_psychotherapy		QoL_improvement_CAPS_reduction_NF/4	1.7525	0	11	Source: Systematic review and meta-analysis NF treatments, under peer review
QoL_improvement_psychotherapy_treatment	Quality of life improvement with psychotherapy treatment	QoL_improvement_CAPS_reduction_psychotherapy/4	1.495	0	30	Source: Morina N, Hoppen TH, Kip A. Study quality and efficacy of psychological interventions for posttraumatic stress disorder: a meta-analysis of randomized controlled trials. Psychological Medicine 2021; 51, 1260–1270. https://doi.org/10.1017/S0033291721001641.
Quarterly_cost_aysmptomatic_PTSD	Quarterly cost mid-mild PTSD	2540	2540	0	2174.75	Quarterly cost asymptomatic PTSD; Source: Marseille E, et al. The cost-effectiveness of MDMA-assisted psychotherapy for the treatment of chronic, treatment resistant PTSD. PLOS ONE. 2020;15(10):e0239997. Note: Costs inflated to 2023 using BLS CPI for outpatient care: Inflation rates: 2020 - 1.023; 2021 - 1.024; 2022 - 1.038; 2023 - 1.073

Name	Description	Formula	Value	Low	High	Comment
Quarterly_cost_mild_PTSD	Quarterly cost mild PTSD	3450	3450	0	2487.25	Source: Marseille E, et al. The cost-effectiveness of MDMA-assisted psychotherapy for the treatment of chronic, treatment resistant PTSD. PLOS ONE. 2020;15(10):e0239997. Note: Costs inflated to 2023 using BLS CPI for outpatient care: Inflation rates: 2020 - 1.023; 2021 - 1.024; 2022 - 1.038; 2023 - 1.073
Quarterly_cost_moderate_PTSD	Quarterly cost moderate PTSD	4900	4900	0	3731	Source: Marseille E, et al. The cost-effectiveness of MDMA-assisted psychotherapy for the treatment of chronic, treatment resistant PTSD. PLOS ONE. 2020;15(10):e0239997. Note: Costs inflated to 2023 using BLS CPI for outpatient care: Inflation rates: 2020 - 1.023; 2021 - 1.024; 2022 - 1.038; 2023 - 1.073
Quarterly_cost_severe_PTSD	Quarterly cost of severe PTSD	5807	5807	0	4972	Source: Marseille E, et al. The cost-effectiveness of MDMA-assisted psychotherapy for the treatment of chronic, treatment resistant PTSD. PLOS ONE. 2020;15(10):e0239997. Note: Costs inflated to 2023 using BLS CPI for outpatient care: Inflation rates: 2020 - 1.023; 2021 - 1.024; 2022 - 1.038; 2023 - 1.073
Quarterly_cost_SSRIs	Quarterly cost SSRIs	Annual_cost_SSRIs/4	88.25	0	3000	
Relative_risk_death_mild_PTSD	Relative risk of death for a person with mild PTSD as defined by CAPS score	1.74	1.74	0	1.74	Marseille E, et al. The cost-effectiveness of MDMA-assisted psychotherapy for the treatment of chronic, treatment resistant PTSD. PLOS ONE. 2020;15(10):e0239997

Name	Description	Formula	Value	Low	High	Comment
Relative_risk_death_moderate_PTSD	Relative risk of death with moderate PTSD	2.05	2.05	0	2.05	Marseille E, et al. The cost-effectiveness of MDMA-assisted psychotherapy for the treatment of chronic, treatment resistant PTSD. PLOS ONE. 2020;15(10):e0239997
Relative_risk_death_severe_PTSD	Relative risk of death with severe PTSD	2.51	2.51	0	2.51	Marseille E, et al. The cost-effectiveness of MDMA-assisted psychotherapy for the treatment of chronic, treatment resistant PTSD. PLOS ONE. 2020;15(10):e0239997
Savings_per_CAPS_reduction	Savings per each CAPS reduction in costs of care	250	250	0	250	Source: Marseille E, et al. The cost-effectiveness of MDMA-assisted psychotherapy for the treatment of chronic, treatment resistant PTSD. PLOS ONE. 2020;15(10):e0239997
Severe_PTSD_Quality_Life_quarterly	Patient with severe PTSD quality of life	Severe_PTSD_QoL/4	0.165	0	0.16875	

Supplementary S4 – distributions NF+OT vs. Psychotherapy

Name	Description	Type	Parameters	EV	Comment
Annual_cost_SSRIs	Annual costs SSRIs	Gamma	alpha: 2, lambda: 1, overriddenMean: 353	353.0	Source: Ivanova JI, et al. Cost of post-traumatic stress disorder vs. major depressive disorder among patients covered by Medicaid or private insurance. AJMC. 2011;17(8):e314-e323. Inflated from 2011 to 2022 using the Medical CPI for prescription drugs
Moderate_PTSD_QoL	Moderate PTSD as defined by QoL	Uniform	subtype: 2, low: 0.72, high: 0.83	0.775	Source: Dams J, et al. Reliability, validity and responsiveness of the EQ-5D-5L in assessing and valuing health status in adolescents and young adults with posttraumatic stress disorder: a randomized controlled trial. Pysch Quart. 2021;92:459-471.
QoL_improvement_CAPS_reduction_NF	QoL improvement per CAPS reduction NF	Triangular	min: 1.36, likeliest: 7.01, max: 12.66	7.01	
Mild_PTSD_QoL	Mild PTSD as defined by QoL	Uniform	subtype: 2, low: 0.93, high: 0.95	0.94	Source: Dams J, et al. Reliability, validity and responsiveness of the EQ-5D-5L in assessing and valuing health status in adolescents and young adults with posttraumatic stress disorder: a randomized controlled trial. Pysch Quart. 2021;92:459-471.
Number_treatment_sessions_NF	Number of treatment sessions for NF	Gamma	alpha: 2, lambda: 1	12	
Prob_treated_PTSD_relapse	Probability of being treated for a relapse of PTSD	Beta	subtype: 2, alpha: 8, beta: 4	0.66667	
Cost_NF_plus_psychotherapy	Cost per session of NF plus psychotherapy - reimbursement	Gamma	alpha: 2, lambda: 1, overriddenMean: 140	140.0	
Severe_PTSD_QoL	Severe PTSD as defined by QoL	Uniform	subtype: 2, low: 0.6, high: 0.72	0.66	Source: Dams J, et al. Reliability, validity and responsiveness of the EQ-5D-5L in assessing and valuing health status in adolescents and young adults with posttraumatic stress disorder: a randomized controlled trial. Pysch Quart. 2021;92:459-471.

Name	Description	Type	Parameters	EV	Comment
Probability_relapse_any_therapy	Probability of relapse after any therapy	Beta	subtype: 2, alpha: 8, beta: 4, overriddenMean: 0.0325	0.0325	Source: Yearly relapse of 0.141 converted to quarterly rate of 0.0325. Levy HC, et al. A meta-analysis of relapse rates in CBT for anxiety disorders. Jrl Anx Dis. 2021;81:102407 @ 14% relapse
Probability_dropout_psychotherapy	Probability of dropout from psychotherapy	Uniform	subtype: 2, low: 0.175, high: 0.24	0.2075	Sources: Varker T, et al. Dropout from guideline-recommended psychological treatments for posttraumatic stress disorder: A systematic review and meta-analysis. Jrl Affect Dis Rep. 2021:100093; Weber M, et al. Long-term outcomes of psychological treatment for post-traumatic stress disorder: a systematic review and meta-analysis. Psych Med. 2021;51:1420-1430.
QoL_improvement_CAPS_reduction_psychotherapy	QoL improvement per CAPS reduction for psychotherapy	Normal	mean: 5.98, stddev: 4.94	5.98	Mean difference reduction on CAPS of 5.98+/-4.94 with psychotherapy. Source: Morina N, Hoppen TH, Kip A. Study quality and efficacy of psychological interventions for posttraumatic stress disorder: a meta-analysis of randomized controlled trials. Psychological Medicine 2021; 51, 1260–1270. https://doi.org/10.1017/S0033291721001641. Each CAPS point reduction, translates into 0.456 QoL improvement. Source: Dams J, et al. Reliability, validity and responsiveness of the EQ-5D-5L in assessing and valuing health status in adolescents and young adults with posttraumatic stress disorder: a randomized controlled trial. Pysch Quart. 2021;92:459-471.
Number_treatment_sessions_psychotherapy		Triangular	min: 8, likeliest: 12, max: 15	11.667	Source: Watkins LE, et al. Treatment PTSD: A review of evidence-based psychotherapy interventions. Fron Behav Neurosci 2018;12:258.

Supplementary S5 – variables NF+OT vs. pharmacotherapy

Name	Description	Formula	Value	Low	High	Comment
Asymptomatic_PTSD_QoL_quarterly	Quality of life patient who is asymptomatic	0.95/4	0.2375	0	0.225	Source: Dams J, et al. Reliability, validity and responsiveness of the EQ-5D-5L in assessing and valuing health status in adolescents and young adults with posttraumatic stress disorder: a randomized controlled trial. Pysch Quart. 2021;92:459-471.
Cost_drugs_quarterly	Cost for pharmacotherapy - SSRIs quarterly	Annual_yearly_cost_SSRIs/4	353.25	0	353.75	
Cost_side_effect_drugs	Cost of an E&M visit to a primary care physician during the year	CPT_99214	90.82	0	90.82	
CPT_90876	CPT payment for NF + psychotherapy	Cost_NF_plus_psychotherapy	2	0	1000	Medicare 2023 estimated rate on fee schedule
CPT_90901	Neurofeedback	41.43	41.43	0	41.43	Medicare 2023 national average payment rate
CPT_90901_booster	Booster session quarterly to maintain asymptomatic status	41.43	41.43	0	41.43	
CPT_99214	CPT 99214 E&M code - established patient	90.82	90.82	0	90.82	CPT code payment for an established patient E&M Medicare 2023
Incidence_death_mid_40s	Incidence of death for a person in their mid 40s	0.0042	0.0042	0.004	0.0042	https://www.cdc.gov/nchs/data/dvs/MortFinal2007_Worktable23r.pdf
Mild_PTSD_Quality_Life_quarterly	QoL patient with mild PTSD	Mild_PTSD_QoL/4	0.2225	0	0.21875	
Moderate_PTSD_Quality_life_quarterly	Moderate PTSD quality of life	Moderate_PTSD_QoL/4	0.19375	0	0.19375	
Number_NF_sessions_treatment	Number of NF treatment session	Number_treatment_sessions_NF	2	0	50	

Name	Description	Formula	Value	Low	High	Comment
Per_CAPS_reduction_QOL_improvement	Per CAPS unit reduction - associated QOL improvement	0.0456	0.0456	0	0.0456	Source: Dams J, et al. Reliability, validity and responsiveness of the EQ-5D-5L in assessing and valuing health status in adolescents and young adults with posttraumatic stress disorder: a randomized controlled trial. Pysch Quart. 2021;92:459-471.
Percent_patients_NF_meds	Percent of patients NF plus meds	0.23	0.23	0	0.23	derived from systematic review and meta-analysis on NF - examination CAPs patients pre-post
Percent_patients_NF_meds_psych	Percent of patients taking medications plus psychotherapy plus NF	0.27	0.27	0	0.27	Derived from systematic review and meta-analysis on NF - examination CAPs patients pre-post
Percent_patients_NF_only	Percent of patients undergoing NF only	0.19	0.19	0	0.19	derived from systematic review and meta-analysis on NF - examination CAPs patients pre-post
Percent_patients_NF_psychotherapy	Percent of patients undergoing NF plus psychotherapy	0.31	0.31	0	0.31	derived from systematic review and meta-analysis on NF - examination CAPs patients pre-post
Prob_dropout_NF_psychotherapy	Probability of dropout of a patient under neurofeeedback and psychotherapy	0.132	0.132	0	0.5	
Prob_dropout_pharmacotherapy	Probability of dropout from pharmacotherapy	0.33	0.33	0	0.4	Source: Williams_T, Phillips_NJ, Stein_DJ, Ipser_JC. Pharmacotherapy for post traumatic stress disorder (PTSD). Cochrane Database of Systematic Reviews 2022, Issue 3. Art. No.: CD002795. DOI: 10.1002/14651858.CD002795.pub3.
Prob_relapse_therapy	Probability of relapse from psychotherapy	Probability_relapse_psych_therapy	0.0325	0	0.2	
Probability_mild_PTSD	Probability of mild PTSD	0.302	0.302	0	0.302	Kessler RC, et al. Prevalence, severity, and co-morbidity of twelve-month DSM-IV disorders in the national comorbidity survey replication (NCS-R). Arch Gen Psych 2005;62(6):617-627.

Name	Description	Formula	Value	Low	High	Comment
Probability_moderate_PTSD	Probability of moderate PTSD	0.332	0.332	0	0.332	Kessler RC, et al. Prevalence, severity, and co-morbidity of twelve-month DSM-IV disorders in the national comorbidity survey replication (NCS-R). Arch Gen Psych 2005;62(6):617-627.
Probability_side_effects_pharmacotherapy	Probability of developing side effects from pharmacotherapy	0.091	0.091	0	0.091	Source: Anderson HD, Pace WD, Libby AM, West DR, Valuck RJ. Rates of 5 common antidepressant side effects among new adult and adolescent cases of depression: a retrospective US claims study. Clin Ther. 2012; 34(1):113–23. https://doi.org/10.1016/j.clinthera.2011.11.024 PMID: 22177545
Probability_SSRI_relapse		Probability_relapse_SSRIs	0.174	0	0.25	
Probability_treatment_post_relapse		Probability_treated_PTSD_relapse	0.667	0	0.5	
QoL_improvement_NF_plus_psychotherapy		QoL_improvement_CAPS_reduction_NF/4	1.7525	0	11	Source: Systematic review and meta-analysis NF treatments, under peer review
QoL_improvement_pharmacotherapy_treatment	Quality of life improvement with pharmacotherapy treatment	QoL_improvement_CAPS_reduction_pharmacotherapy/4	1.66	0	15	Source: Morina N, Hoppen TH, Kip A. Study quality and efficacy of psychological interventions for posttraumatic stress disorder: a meta-analysis of randomized controlled trials. Psychological Medicine 2021; 51, 1260–1270. https://doi.org/10.1017/S0033291721001641.
Quarterly_cost_aysmptomatic_PTSD	Quarterly cost mid-mild PTSD	2540	2540	0	2174.75	Quarterly cost asymptomatic PTSD; Source: Marseille E, et al. The cost-effectiveness of MDMA-assisted psychotherapy for the treatment of chronic, treatment resistant PTSD. PLOS ONE. 2020;15(10):e0239997. Note: Costs inflated to 2023 using BLS CPI for outpatient care: Inflation rates: 2020 - 1.023; 2021 - 1.024; 2022 - 1.038; 2023 - 1.073

Name	Description	Formula	Value	Low	High	Comment
Quarterly_cost_mild_PTSD	Quarterly cost mild PTSD	3450	3450	0	2487.25	Source: Marseille E, et al. The cost-effectiveness of MDMA-assisted psychotherapy for the treatment of chronic, treatment resistant PTSD. PLOS ONE. 2020;15(10):e0239997. Note: Costs inflated to 2023 using BLS CPI for outpatient care: Inflation rates: 2020 - 1.023; 2021 - 1.024; 2022 - 1.038; 2023 - 1.073
Quarterly_cost_moderate_PTSD	Quarterly cost moderate PTSD	4900	4900	0	6000	Source: Marseille E, et al. The cost-effectiveness of MDMA-assisted psychotherapy for the treatment of chronic, treatment resistant PTSD. PLOS ONE. 2020;15(10):e0239997. Note: Costs inflated to 2023 using BLS CPI for outpatient care: Inflation rates: 2020 - 1.023; 2021 - 1.024; 2022 - 1.038; 2023 - 1.073
Quarterly_cost_severe_PTSD	Quarterly cost of severe PTSD	5807	5807	0	9000	Source: Marseille E, et al. The cost-effectiveness of MDMA-assisted psychotherapy for the treatment of chronic, treatment resistant PTSD. PLOS ONE. 2020;15(10):e0239997. Note: Costs inflated to 2023 using BLS CPI for outpatient care: Inflation rates: 2020 - 1.023; 2021 - 1.024; 2022 - 1.038; 2023 - 1.073
Relative_risk_death_mild_PTSD	Relative risk of death for a person with mild PTSD as defined by CAPS score	1.74	1.74	1.7	1.74	Marseille E, et al. The cost-effectiveness of MDMA-assisted psychotherapy for the treatment of chronic, treatment resistant PTSD. PLOS ONE. 2020;15(10):e0239997
Relative_risk_death_moderate_PTSD	Relative risk of death with moderate PTSD	2.05	2.05	0	2.05	Marseille E, et al. The cost-effectiveness of MDMA-assisted psychotherapy for the treatment of chronic, treatment resistant PTSD. PLOS ONE. 2020;15(10):e0239997
Relative_risk_death_severe_PTSD	Relative risk of death with severe PTSD	2.51	2.51	0	4	Marseille E, et al. The cost-effectiveness of MDMA-assisted psychotherapy for the treatment of chronic, treatment resistant PTSD. PLOS ONE. 2020;15(10):e0239997

Name	Description	Formula	Value	Low	High	Comment
Savings_per_CAPS_reduction	Savings per each CAPS reduction in costs of care	250	250	0	1000	Source: Marseille E, et al. The cost-effectiveness of MDMA-assisted psychotherapy for the treatment of chronic, treatment resistant PTSD. PLOS ONE. 2020;15(10):e0239997
Severe_PTSD_Quality_Life_quarterly	Patient with severe PTSD quality of life	Severe_PTSD_QoL/4	0.165	0	0.16875	

Supplementary S5 – distributions NF+OT vs. Pharmacotherapy

**Name**	**Description**	**Type**	**Parameters**	**EV**	**Comment**
Probability_relapse_psych_therapy	Probability of relapse after psychotherapy	Beta	subtype: 2, alpha: 8, beta: 4, overriddenMean: 0.0325	0.0325	Source: Levy HC, et al. A meta-analysis of relapse rates in CBT for anxiety disorders. Jrl Anx Dis. 2021;81:102407 @ 14% relapse. Converted to a quarterly rate of 0.0325 (.141/4)
Severe_PTSD_QoL	Severe PTSD as defined by QoL	Uniform	subtype: 2, low: 0.6, high: 0.72	0.66	Source: Dams J, et al. Reliability, validity and responsiveness of the EQ-5D-5L in assessing and valuing health status in adolescents and young adults with posttraumatic stress disorder: a randomized controlled trial. Pysch Quart. 2021;92:459-471.
QoL_improvement_CAPS_reduction_pharmacotherapy	QoL improvement per CAPS reduction for pharmacotherapy	Uniform	subtype: 2, low: 4.16, high: 9.11, overriddenMean: 6.64	6.64	Source: Ipser JC et al. Evidence-based pharmacotherapy of post-traumatic stress disorder (PTSD). Int Jr. Neuropsychopharma. 2012;15:825-840.
Moderate_PTSD_QoL	Moderate PTSD as defined by QoL	Uniform	subtype: 2, low: 0.72, high: 0.83	0.775	Source: Dams J, et al. Reliability, validity and responsiveness of the EQ-5D-5L in assessing and valuing health status in adolescents and young adults with posttraumatic stress disorder: a randomized controlled trial. Pysch Quart. 2021;92:459-471.
QoL_improvement_CAPS_reduction_NF	QoL improvement per CAPS reduction NF	Triangular	min: 1.36, likeliest: 7.01, max: 12.66	7.01	

Mild_PTSD_QoL	Mild PTSD as defined by QoL	Uniform	subtype: 2, low: 0.83, high: 0.95	0.89	Source: Dams J, et al. Reliability, validity and responsiveness of the EQ-5D-5L in assessing and valuing health status in adolescents and young adults with posttraumatic stress disorder: a randomized controlled trial. Pysch Quart. 2021;92:459-471.
Number_treatment_sessions_NF	Number of treatment sessions for NF	Gamma	alpha: 2, lambda: 1	2.0	
Annual_yearly_cost_SSRIs	Annual cost per year of SSRIs	Gamma	alpha: 2, lambda: 1, overriddenMean: 1413	1413.0	Source: Ivanova JI, et al. Cost of post-traumatic stress disorder vs. major depressive disorder among patients covered by Medicaid or private insurance. AJMC. 2011;17(8):e314-e323. Inflated from 2011 to 2022 using the Medical CPI for prescription drugs
Cost_NF_plus_psychotherapy	Cost per session of NF plus psychotherapy - reimbursement	Gamma	alpha: 2, lambda: 1	2.0	
Probability_treated_PTSD_relapse	Probability receiving treatment for PTSD relapse	Beta	subtype: 2, alpha: 8, beta: 4	0.66667	
Probability_relapse_SSRIs	Probability replace with SSRI treatment	Normal	mean: 0.174, stddev: 0.05	0.174	Source: Davidson J, et al. Efficacy of sertraline in preventing relapse of posttraumatic stress disorder. Amer Jr. Psych. 2001;158:1974-1981.

Supplementary S6 – calculations used in each comparison model

NF + adjunctive therapy v. psychotherapy calculations

Health state	Reward	Therapy state for NF+adjunctive
Less mild/asymptomatic	Initial cost; stage 0	Quarterly_cost_aysmptomatic_PTSD+(Percent_NF_meds+Percent_NF_meds_psych)*Quarterly_cost_SSRIs+CPT_99214*(Percent_NF_meds+Percent_NF_meds_psych)*Probability_side_effects_pharmacotherapy
	Incremental cost; stages 1-11	Quarterly_cost_aysmptomatic_PTSD+(Percent_NF_meds+Percent_NF_meds_psych)*Quarterly_cost_SSRIs+CPT_99214*(Percent_NF_meds+Percent_NF_meds_psych)*Probability_side_effects_pharmacotherapy+CPT_90901_booster
	Final cost; stage 12	Quarterly_cost_aysmptomatic_PTSD+(Percent_NF_meds+Percent_NF_meds_psych)*Quarterly_cost_SSRIs+CPT_99214*(Percent_NF_meds+Percent_NF_meds_psych)*Probability_side_effects_pharmacotherapy+CPT_90901_booster
	Initial effectiveness; stage 0	Asymptomatic_PTSD_QoL_quarterly
	Incremental effectiveness; states 1-11	Asymptomatic_PTSD_QoL_quarterly
	Final effectiveness; stage 12	Asymptomatic_PTSD_QoL_quarterly
Mild PTSD treatment	Initial cost; stage 0	If(Mild_PTSD_Quality_Life_quarterly+QoL_improvement_NF_plus_psychotherapy*Per_CAPS_reduction_QOL_improvement/4>=0.2375;Quarterly_cost_aysmptomatic_PTSD;Quarterly_cost_mild_PTSD)+CPT_90876*Number_NF_sesssions_treatment*Percent_NF_psychotherapy+Quarterly_cost_SSRIs*Percent_NF_meds+CPT_90901*Number_NF_sesssions_treatment*Percent_NF+(CPT_90876*Number_NF_sesssions_treatment+Quarterly_cost_SSRIs)*Percent_NF_meds_psych+CPT_99214*(Percent_NF_meds+Percent_NF_meds_psych)*Probability_side_effects_pharmacotherapy

Health state	Reward	Therapy state for NF+adjunctive
	Incremental cost; stages 1-11	If(Mild_PTSD_Quality_Life_quarterly+QoL_improvement_NF_plus_psychotherapy*Per_CAPS_reduction_QOL_improvement/4>=0.2375;Quarterly_cost_aysmptomatic_PTSD;Quarterly_cost_mild_PTSD)+(Percent_NF_meds+Percent_NF_meds_psych)*Quarterly_cost_SSRIs+CPT_99214*(Percent_NF_meds+Percent_NF_meds_psych)*Probability_side_effects_pharmacotherapy
	Final cost; stage 12	If(Mild_PTSD_Quality_Life_quarterly+QoL_improvement_NF_plus_psychotherapy*Per_CAPS_reduction_QOL_improvement/4>=0.2375;Quarterly_cost_aysmptomatic_PTSD;Quarterly_cost_mild_PTSD)+(Percent_NF_meds+Percent_NF_meds_psych)*Quarterly_cost_SSRIs+CPT_99214*(Percent_NF_meds+Percent_NF_meds_psych)*Probability_side_effects_pharmacotherapy
	Initial effectiveness; stage 0	If(Mild_PTSD_Quality_Life_quarterly+Per_CAPS_reduction_QOL_improvement*QoL_improvement_NF_plus_psychotherapy/4>=0.2375;Asymptomatic_PTSD_QoL_quarterly;Mild_PTSD_Quality_Life_quarterly)
	Incremental effectiveness; states 1-11	If(Mild_PTSD_Quality_Life_quarterly+Per_CAPS_reduction_QOL_improvement*QoL_improvement_NF_plus_psychotherapy/4>=0.2375;Asymptomatic_PTSD_QoL_quarterly;Mild_PTSD_Quality_Life_quarterly)
	Final effectiveness; stage 12	If(Mild_PTSD_Quality_Life_quarterly+Per_CAPS_reduction_QOL_improvement*QoL_improvement_NF_plus_psychotherapy/4>=0.2375;Asymptomatic_PTSD_QoL_quarterly;Mild_PTSD_Quality_Life_quarterly)

Health state	Reward	Therapy state for NF+adjunctive
Moderate PTSD treatment	Initial cost; stage 0	If(Moderate_PTSD_Quality_life_quarterly+QoL_improvement_NF_plus_psychotherapy*Per_CAPS_reduction_QOL_improvement/4>=0.2075;Quarterly_cost_mild_PTSD;Quarterly_cost_moderate_PTSD)+CPT_90876*Number_NF_sesssions_treatment*Percent_NF_psychotherapy+Quarterly_cost_SSRIs*Percent_NF_meds+CPT_90901*Number_NF_sesssions_treatment*Percent_NF+(CPT_90876*Number_NF_sesssions_treatment+Quarterly_cost_SSRIs)*Percent_NF_meds_psych+CPT_99214*(Percent_NF_meds+Percent_NF_meds_psych)*Probability_side_effects_pharmacotherapy
	Incremental cost; stages 1-11	If(Moderate_PTSD_Quality_life_quarterly+QoL_improvement_NF_plus_psychotherapy*Per_CAPS_reduction_QOL_improvement/4>=0.2075;Quarterly_cost_mild_PTSD;Quarterly_cost_moderate_PTSD)+(Percent_NF_meds+Percent_NF_meds_psych)*Quarterly_cost_SSRIs+CPT_99214*(Percent_NF_meds+Percent_NF_meds_psych)*Probability_side_effects_pharmacotherapy
	Final cost; stage 12	If(Moderate_PTSD_Quality_life_quarterly+QoL_improvement_NF_plus_psychotherapy*Per_CAPS_reduction_QOL_improvement/4>=0.2075;Quarterly_cost_mild_PTSD;Quarterly_cost_moderate_PTSD)+(Percent_NF_meds+Percent_NF_meds_psych)*Quarterly_cost_SSRIs+CPT_99214*(Percent_NF_meds+Percent_NF_meds_psych)*Probability_side_effects_pharmacotherapy
	Initial effectiveness; stage 0	If(Moderate_PTSD_Quality_life_quarterly+Per_CAPS_reduction_QOL_improvement*QoL_improvement_NF_plus_psychotherapy/4>=0.2075;Mild_PTSD_Quality_Life_quarterly;Moderate_PTSD_Quality_life_quarterly)
	Incremental effectiveness; states 1-11	If(Moderate_PTSD_Quality_life_quarterly+Per_CAPS_reduction_QOL_improvement*QoL_improvement_NF_plus_psychotherapy/4>=0.2075;Mild_PTSD_Quality_Life_quarterly;Moderate_PTSD_Quality_life_quarterly)

Health state	Reward	Therapy state for NF+adjunctive
	Final effectiveness; stage 12	If(Moderate_PTSD_Quality_life_quarterly+Per_CAPS_reduction_QOL_improvement*QoL_improvement_NF_plus_psychotherapy/4>=0.2075;Mild_PTSD_Quality_Life_quarterly;Moderate_PTSD_Quality_life_quarterly)
Severe PTSD treatment	Initial cost; stage 0	If(Severe_PTSD_Quality_Life_quarterly+QoL_improvement_NF_plus_psychotherapy*Per_CAPS_reduction_QOL_improvement/4>=0.18;Quarterly_cost_moderate_PTSD;Quarterly_cost_severe_PTSD)+CPT_90876*Number_NF_sesssions_treatment*Percent_NF_psychotherapy+Quarterly_cost_SSRIs*Percent_NF_meds+CPT_90901*Number_NF_sesssions_treatment*Percent_NF+(CPT_90876*Number_NF_sesssions_treatment+Quarterly_cost_SSRIs)*Percent_NF_meds_psych+CPT_99214*(Percent_NF_meds+Percent_NF_meds_psych)*Probability_side_effects_pharmacotherapy
	Incremental cost; stages 1-11	If(Severe_PTSD_Quality_Life_quarterly+QoL_improvement_NF_plus_psychotherapy*Per_CAPS_reduction_QOL_improvement/4>=0.18;Quarterly_cost_moderate_PTSD;Quarterly_cost_severe_PTSD)+(Percent_NF_meds+Percent_NF_meds_psych)*Quarterly_cost_SSRIs+CPT_99214*(Percent_NF_meds+Percent_NF_meds_psych)*Probability_side_effects_pharmacotherapy
	Final cost; stage 12	If(Severe_PTSD_Quality_Life_quarterly+QoL_improvement_NF_plus_psychotherapy*Per_CAPS_reduction_QOL_improvement/4>=0.18;Quarterly_cost_moderate_PTSD;Quarterly_cost_severe_PTSD)+(Percent_NF_meds+Percent_NF_meds_psych)*Quarterly_cost_SSRIs+CPT_99214*(Percent_NF_meds+Percent_NF_meds_psych)*Probability_side_effects_pharmacotherapy

Health state	Reward	Therapy state for NF+adjunctive
	Initial effectiveness; stage 0	If(Severe_PTSD_Quality_Life_quarterly+Per_CAPS_reduction_QOL_improvement*QoL_improvement_NF_plus_psychotherapy/4>=0.18;Moderate_PTSD_Quality_life_quarterly;Severe_PTSD_Quality_Life_quarterly)
	Incremental effectiveness; states 1-11	If(Severe_PTSD_Quality_Life_quarterly+Per_CAPS_reduction_QOL_improvement*QoL_improvement_NF_plus_psychotherapy/4>=0.18;Moderate_PTSD_Quality_life_quarterly;Severe_PTSD_Quality_Life_quarterly)
	Final effectiveness; stage 12	If(Severe_PTSD_Quality_Life_quarterly+Per_CAPS_reduction_QOL_improvement*QoL_improvement_NF_plus_psychotherapy/4>=0.18;Moderate_PTSD_Quality_life_quarterly;Severe_PTSD_Quality_Life_quarterly)
Mild PTSD state	Initial cost; stage 0	0
	Incremental cost; stages 1-11	Quarterly_cost_mild_PTSD
	Final cost; stage 12	Quarterly_cost_mild_PTSD
	Initial effectiveness; stage 0	0
	Incremental effectiveness; states 1-11	Mild_PTSD_Quality_Life_quarterly
	Final effectiveness; stage 12	Mild_PTSD_Quality_Life_quarterly
Moderate PTSD state	Initial cost; stage 0	0
	Incremental cost; stages 1-11	Quarterly_cost_moderate_PTSD
	Final cost; stage 12	Quarterly_cost_moderate_PTSD
	Initial effectiveness; stage 0	0
	Incremental effectiveness; states 1-11	Moderate_PTSD_Quality_life_quarterly
	Final effectiveness; stage 12	Moderate_PTSD_Quality_life_quarterly
Severe PTSD state	Initial cost; stage 0	0
	Incremental cost; stages 1-11	Quarterly_cost_severe_PTSD
	Final cost; stage 12	Quarterly_cost_severe_PTSD
	Initial effectiveness; stage 0	0
	Incremental effectiveness; states 1-11	Severe_PTSD_Quality_Life_quarterly
	Final effectiveness; stage 12	Severe_PTSD_Quality_Life_quarterly

Health state	Reward	Therapy state for NF+adjunctive
Dead	Initial cost; stage 0	0
	Incremental cost; stages 1-11	0
	Final cost; stage 12	0
	Initial effectiveness; stage 0	0
	Incremental effectiveness; states 1-11	0
	Final effectiveness; stage 12	0
Relapse Mild PTSD	Initial cost; stage 0	0
	Incremental cost; stages 1-11	0
	Final cost; stage 12	0
	Initial effectiveness; stage 0	0
	Incremental effectiveness; states 1-11	0
	Final effectiveness; stage 12	0
Relapse Moderate PTSD	Initial cost; stage 0	0
	Incremental cost; stages 1-11	0
	Final cost; stage 12	0
	Initial effectiveness; stage 0	0
	Incremental effectiveness; states 1-11	0
	Final effectiveness; stage 12	0
Relapse Severe PTSD	Initial cost; stage 0	0
	Incremental cost; stages 1-11	0
	Final cost; stage 12	0
	Initial effectiveness; stage 0	0
	Incremental effectiveness; states 1-11	0
	Final effectiveness; stage 12	0

Health state	Reward	Therapy state for Psychotherapy
Less mild/asymptomatic	Initial cost; stage 0	Quarterly_cost_aysmptomatic_PTSD
	Incremental cost; stages 1-11	Quarterly_cost_aysmptomatic_PTSD
	Final cost; stage 12	Quarterly_cost_aysmptomatic_PTSD
	Initial effectiveness; stage 0	Asymptomatic_PTSD_QoL_quarterly
	Incremental effectiveness; states 1-11	Asymptomatic_PTSD_QoL_quarterly
	Final effectiveness; stage 12	Asymptomatic_PTSD_QoL_quarterly

Health state	Reward	Therapy state for Psychotherapy
Mild PTSD treatment	Initial cost; stage 0	If(Mild_PTSD_Quality_Life_quarterly+QoL_improvement_psychotherapy_treatment*Per_CAPS_reduction_QOL_improvement/4>=0.2375;Quarterly_cost_aysmptomatic_PTSD;Quarterly_cost_mild_PTSD)+CPT_90837*Number_psychotherapy_sessions_treatment
	Incremental cost; stages 1-11	If(Mild_PTSD_Quality_Life_quarterly+QoL_improvement_psychotherapy_treatment*Per_CAPS_reduction_QOL_improvement/4>=0.2375;Quarterly_cost_aysmptomatic_PTSD;Quarterly_cost_mild_PTSD)
	Final cost; stage 12	If(Mild_PTSD_Quality_Life_quarterly+QoL_improvement_psychotherapy_treatment*Per_CAPS_reduction_QOL_improvement/4>=0.2375;Quarterly_cost_aysmptomatic_PTSD;Quarterly_cost_mild_PTSD)
	Initial effectiveness; stage 0	If(Mild_PTSD_Quality_Life_quarterly+QoL_improvement_psychotherapy_treatment*Per_CAPS_reduction_QOL_improvement/4>=0.2375;Asymptomatic_PTSD_QoL_quarterly;Mild_PTSD_Quality_Life_quarterly)
	Incremental effectiveness; states 1-11	If(Mild_PTSD_Quality_Life_quarterly+QoL_improvement_psychotherapy_treatment*Per_CAPS_reduction_QOL_improvement/4>=0.2375;Asymptomatic_PTSD_QoL_quarterly;Mild_PTSD_Quality_Life_quarterly)
	Final effectiveness; stage 12	If(Mild_PTSD_Quality_Life_quarterly+QoL_improvement_psychotherapy_treatment*Per_CAPS_reduction_QOL_improvement/4>=0.2375;Asymptomatic_PTSD_QoL_quarterly;Mild_PTSD_Quality_Life_quarterly)
Moderate PTSD treatment	Initial cost; stage 0	If(Moderate_PTSD_Quality_life_quarterly+Per_CAPS_reduction_QOL_improvement*QoL_improvement_psychotherapy_treatment/4>=0.2075;Quarterly_cost_mild_PTSD;Quarterly_cost_moderate_PTSD)+CPT_90837*Number_psychotherapy_sessions_treatment

Health state	Reward	Therapy state for Psychotherapy
	Incremental cost; stages 1-11	If(Moderate_PTSD_Quality_life_quarterly+Per_CAPS_reduction_QOL_improvement*QoL_improvement_psychotherapy_treatment/4>=0.2075;Quarterly_cost_mild_PTSD;Quarterly_cost_moderate_PTSD)+CPT_90837*Number_psychotherapy_sessions_treatment
	Final cost; stage 12	If(Moderate_PTSD_Quality_life_quarterly+Per_CAPS_reduction_QOL_improvement*QoL_improvement_psychotherapy_treatment/4>=0.2075;Quarterly_cost_mild_PTSD;Quarterly_cost_moderate_PTSD)
	Initial effectiveness; stage 0	If(Moderate_PTSD_Quality_life_quarterly+Per_CAPS_reduction_QOL_improvement*QoL_improvement_psychotherapy_treatment/4>=0.2075;Mild_PTSD_Quality_Life_quarterly;Moderate_PTSD_Quality_life_quarterly)
	Incremental effectiveness; states 1-11	If(Moderate_PTSD_Quality_life_quarterly+Per_CAPS_reduction_QOL_improvement*QoL_improvement_psychotherapy_treatment/4>=0.2075;Mild_PTSD_Quality_Life_quarterly;Moderate_PTSD_Quality_life_quarterly)
	Final effectiveness; stage 12	If(Moderate_PTSD_Quality_life_quarterly+Per_CAPS_reduction_QOL_improvement*QoL_improvement_psychotherapy_treatment/4>=0.2075;Mild_PTSD_Quality_Life_quarterly;Moderate_PTSD_Quality_life_quarterly)
Severe PTSD treatment	Initial cost; stage 0	If(Severe_PTSD_Quality_Life_quarterly+Per_CAPS_reduction_QOL_improvement*QoL_improvement_psychotherapy_treatment/4>=0.18;Quarterly_cost_moderate_PTSD;Quarterly_cost_severe_PTSD)+CPT_90837*Number_psychotherapy_sessions_treatment
	Incremental cost; stages 1-11	If(Severe_PTSD_Quality_Life_quarterly+Per_CAPS_reduction_QOL_improvement*QoL_improvement_psychotherapy_treatment/4>=0.18;Quarterly_cost_moderate_PTSD;Quarterly_cost_severe_PTSD)

Health state	Reward	Therapy state for Psychotherapy
	Final cost; stage 12	If(Severe_PTSD_Quality_Life_quarterly+Per_CAPS_reduction_QOL_improvement*QoL_improvement_psychotherapy_treatment/4>=0.18;Quarterly_cost_moderate_PTSD;Quarterly_cost_severe_PTSD)
	Initial effectiveness; stage 0	If(Severe_PTSD_Quality_Life_quarterly+Per_CAPS_reduction_QOL_improvement*QoL_improvement_psychotherapy_treatment/4>=0.18;Moderate_PTSD_Quality_life_quarterly;Severe_PTSD_Quality_Life_quarterly)
	Incremental effectiveness; states 1-11	If(Severe_PTSD_Quality_Life_quarterly+Per_CAPS_reduction_QOL_improvement*QoL_improvement_psychotherapy_treatment/4>=0.18;Moderate_PTSD_Quality_life_quarterly;Severe_PTSD_Quality_Life_quarterly)
	Final effectiveness; stage 12	If(Severe_PTSD_Quality_Life_quarterly+Per_CAPS_reduction_QOL_improvement*QoL_improvement_psychotherapy_treatment/4>=0.18;Moderate_PTSD_Quality_life_quarterly;Severe_PTSD_Quality_Life_quarterly)
Mild PTSD state	Initial cost; stage 0	0
	Incremental cost; stages 1-11	Quarterly_cost_mild_PTSD
	Final cost; stage 12	Quarterly_cost_mild_PTSD
	Initial effectiveness; stage 0	0
	Incremental effectiveness; states 1-11	Mild_PTSD_Quality_Life_quarterly
	Final effectiveness; stage 12	Mild_PTSD_Quality_Life_quarterly
Moderate PTSD state	Initial cost; stage 0	0
	Incremental cost; stages 1-11	Mild_PTSD_Quality_Life_quarterly
	Final cost; stage 12	Mild_PTSD_Quality_Life_quarterly
	Initial effectiveness; stage 0	0
	Incremental effectiveness; states 1-11	Quarterly_cost_moderate_PTSD
	Final effectiveness; stage 12	Quarterly_cost_moderate_PTSD

Health state	Reward	Therapy state for Psychotherapy
Severe PTSD state	Initial cost; stage 0	0
	Incremental cost; stages 1-11	Quarterly_cost_severe_PTSD
	Final cost; stage 12	Quarterly_cost_severe_PTSD
	Initial effectiveness; stage 0	0
	Incremental effectiveness; states 1-11	Severe_PTSD_Quality_Life_quarterly
	Final effectiveness; stage 12	Severe_PTSD_Quality_Life_quarterly
Dead	Initial cost; stage 0	0
	Incremental cost; stages 1-11	0
	Final cost; stage 12	0
	Initial effectiveness; stage 0	0
	Incremental effectiveness; states 1-11	0
	Final effectiveness; stage 12	0
Relapse Mild PTSD	Initial cost; stage 0	0
	Incremental cost; stages 1-11	0
	Final cost; stage 12	0
	Initial effectiveness; stage 0	0
	Incremental effectiveness; states 1-11	0
	Final effectiveness; stage 12	0
Relapse Moderate PTSD	Initial cost; stage 0	0
	Incremental cost; stages 1-11	0
	Final cost; stage 12	0
	Initial effectiveness; stage 0	0
	Incremental effectiveness; states 1-11	0
	Final effectiveness; stage 12	0
Relapse Severe PTSD	Initial cost; stage 0	0
	Incremental cost; stages 1-11	0
	Final cost; stage 12	0
	Initial effectiveness; stage 0	0

Health state	Reward	Therapy state for Psychotherapy
	Incremental effectiveness; states 1-11	0
	Final effectiveness; stage 12	0

NF + adjunctive therapy v. pharmacotherapy calculations

Health state	Reward	Therapy state for NF+adjunctive
Less mild/asymptomatic	Initial cost; stage 0	Quarterly_cost_aysmptomatic_PTSD+Cost_drugs_quarterly*(Percent_patients_NF_meds+Percent_patients_NF_meds_psych)+CPT_99214*(Percent_patients_NF_meds+Percent_patients_NF_meds_psych)*Probability_side_effects_pharmacotherapy
	Incremental cost; stages 1-11	Quarterly_cost_aysmptomatic_PTSD+Cost_drugs_quarterly*(Percent_patients_NF_meds+Percent_patients_NF_meds_psych)+CPT_99214*(Percent_patients_NF_meds+Percent_patients_NF_meds_psych)*Probability_side_effects_pharmacotherapy
	Final cost; stage 12	Quarterly_cost_aysmptomatic_PTSD+Cost_drugs_quarterly*(Percent_patients_NF_meds+Percent_patients_NF_meds_psych)+CPT_99214*(Percent_patients_NF_meds+Percent_patients_NF_meds_psych)*Probability_side_effects_pharmacotherapy
	Initial effectiveness; stage 0	Asymptomatic_PTSD_QoL_quarterly
	Incremental effectiveness; states 1-11	Asymptomatic_PTSD_QoL_quarterly
	Final effectiveness; stage 12	Asymptomatic_PTSD_QoL_quarterly
Mild PTSD treatment	Initial cost; stage 0	If(Mild_PTSD_Quality_Life_quarterly+QoL_improvement_NF_plus_psychotherapy*Per_CAPS_reduction_QOL_improvement/4>=0.2375;Quarterly_cost_aysmptomatic_PTSD;Quarterly_cost_mild_PTSD)+CPT_90876*Number_NF_sessions_treatment*Percent_patients_NF_psychotherapy+Cost_drugs_quarterly*Percent_patients_NF_meds+CPT_90901*Number_NF_sessions_treatment*Percent_patients_NF_only+(CPT_

Health state	Reward	Therapy state for NF+adjunctive
		90876*Number_NF_sessions_treatment+Cost_drugs_quarterly)*Percent_patients_NF_meds_psych+CPT_99214*(Percent_patients_NF_meds+Percent_patients_NF_meds_psych)*Probability_side_effects_pharmacotherapy
	Incremental cost; stages 1-11	If(Mild_PTSD_Quality_Life_quarterly+QoL_improvement_NF_plus_psychotherapy*Per_CAPS_reduction_QOL_improvement/4>=0.2375;Quarterly_cost_aysmptomatic_PTSD;Quarterly_cost_mild_PTSD)+Cost_drugs_quarterly*(Percent_patients_NF_meds+Percent_patients_NF_meds_psych)+CPT_99214*(Percent_patients_NF_meds+Percent_patients_NF_meds_psych)*Probability_side_effects_pharmacotherapy
	Final cost; stage 12	If(Mild_PTSD_Quality_Life_quarterly+QoL_improvement_NF_plus_psychotherapy*Per_CAPS_reduction_QOL_improvement/4>=0.2375;Quarterly_cost_aysmptomatic_PTSD;Quarterly_cost_mild_PTSD)+Cost_drugs_quarterly*(Percent_patients_NF_meds+Percent_patients_NF_meds_psych)+CPT_99214*(Percent_patients_NF_meds+Percent_patients_NF_meds_psych)*Probability_side_effects_pharmacotherapy
	Initial effectiveness; stage 0	If(Mild_PTSD_Quality_Life_quarterly+Per_CAPS_reduction_QOL_improvement*QoL_improvement_NF_plus_psychotherapy/4>=0.2375;Asymptomatic_PTSD_QoL_quarterly;Mild_PTSD_Quality_Life_quarterly)
	Incremental effectiveness; states 1-11	If(Mild_PTSD_Quality_Life_quarterly+Per_CAPS_reduction_QOL_improvement*QoL_improvement_NF_plus_psychotherapy/4>=0.2375;Asymptomatic_PTSD_QoL_quarterly;Mild_PTSD_Quality_Life_quarterly)

Health state	Reward	Therapy state for NF+adjunctive
	Final effectiveness; stage 12	If(Mild_PTSD_Quality_Life_quarterly+Per_CAPS_reduction_QOL_improvement*QoL_improvement_NF_plus_psychotherapy/4>=0.2375;Asymptomatic_PTSD_QoL_quarterly;Mild_PTSD_Quality_Life_quarterly)
Moderate PTSD treatment	Initial cost; stage 0	If(Moderate_PTSD_Quality_life_quarterly+QoL_improvement_NF_plus_psychotherapy*Per_CAPS_reduction_QOL_improvement/4>=0.2075;Quarterly_cost_mild_PTSD;Quarterly_cost_moderate_PTSD)+CPT_90876*Number_NF_sessions_treatment*Percent_patients_NF_psychotherapy+Cost_drugs_quarterly*Percent_patients_NF_meds+CPT_90901*Number_NF_sessions_treatment*Percent_patients_NF_only+(CPT_90876*Number_NF_sessions_treatment+Cost_drugs_quarterly)*Percent_patients_NF_meds_psych+CPT_99214*(Percent_patients_NF_meds+Percent_patients_NF_meds_psych)*Probability_side_effects_pharmacotherapy
	Incremental cost; stages 1-11	If(Moderate_PTSD_Quality_life_quarterly+QoL_improvement_NF_plus_psychotherapy*Per_CAPS_reduction_QOL_improvement/4>=0.2075;Quarterly_cost_mild_PTSD;Quarterly_cost_moderate_PTSD)+Cost_drugs_quarterly*(Percent_patients_NF_meds+Percent_patients_NF_meds_psych)+CPT_99214*(Percent_patients_NF_meds+Percent_patients_NF_meds_psych)*Probability_side_effects_pharmacotherapy
	Final cost; stage 12	If(Moderate_PTSD_Quality_life_quarterly+QoL_improvement_NF_plus_psychotherapy*Per_CAPS_reduction_QOL_improvement/4>=0.2075;Quarterly_cost_mild_PTSD;Quarterly_cost_moderate_PTSD)+Cost_drugs_quarterly*(Percent_patients_NF_meds+Percent_patients_NF_meds_psych)+CPT_99214*(Percent_patients_NF_meds+Percent_patients_NF_meds_psych)*Probability_side_effects_pharmacotherapy

Health state	Reward	Therapy state for NF+adjunctive
	Initial effectiveness; stage 0	If(Moderate_PTSD_Quality_life_quarterly+Per_CAPS_reduction_QOL_improvement*QoL_improvement_NF_plus_psychotherapy/4>=0.2075;Mild_PTSD_Quality_Life_quarterly;Moderate_PTSD_Quality_life_quarterly)
	Incremental effectiveness; states 1-11	If(Moderate_PTSD_Quality_life_quarterly+Per_CAPS_reduction_QOL_improvement*QoL_improvement_NF_plus_psychotherapy/4>=0.2075;Mild_PTSD_Quality_Life_quarterly;Moderate_PTSD_Quality_life_quarterly)
	Final effectiveness; stage 12	If(Moderate_PTSD_Quality_life_quarterly+Per_CAPS_reduction_QOL_improvement*QoL_improvement_NF_plus_psychotherapy/4>=0.2075;Mild_PTSD_Quality_Life_quarterly;Moderate_PTSD_Quality_life_quarterly)
Severe PTSD treatment	Initial cost; stage 0	If(Severe_PTSD_Quality_Life_quarterly+QoL_improvement_NF_plus_psychotherapy*Per_CAPS_reduction_QOL_improvement/4>=0.18;Quarterly_cost_moderate_PTSD;Quarterly_cost_severe_PTSD)+CPT_90876*Number_NF_sessions_treatment*Percent_patients_NF_psychotherapy+Cost_drugs_quarterly*Percent_patients_NF_meds+CPT_90901*Number_NF_sessions_treatment*Percent_patients_NF_only+(CPT_90876*Number_NF_sessions_treatment+Cost_drugs_quarterly)*Percent_patients_NF_meds_psych+CPT_99214*(Percent_patients_NF_meds+Percent_patients_NF_meds_psych)*Probability_side_effects_pharmacotherapy

Health state	Reward	Therapy state for NF+adjunctive
	Incremental cost; stages 1-11	If(Severe_PTSD_Quality_Life_quarterly+QoL_improvement_NF_plus_psychotherapy*Per_CAPS_reduction_QOL_improvement/4>=0.18;Quarterly_cost_moderate_PTSD;Quarterly_cost_severe_PTSD)+CPT_90876*Number_NF_sessions_treatment*Percent_patients_NF_psychotherapy+Cost_drugs_quarterly*Percent_patients_NF_meds+CPT_90901*Number_NF_sessions_treatment*Percent_patients_NF_only+(CPT_90876*Number_NF_sessions_treatment+Cost_drugs_quarterly)*Percent_patients_NF_meds_psych+CPT_99214*(Percent_patients_NF_meds+Percent_patients_NF_meds_psych)*Probability_side_effects_pharmacotherapy
	Final cost; stage 12	If(Severe_PTSD_Quality_Life_quarterly+QoL_improvement_NF_plus_psychotherapy*Per_CAPS_reduction_QOL_improvement/4>=0.18;Quarterly_cost_moderate_PTSD;Quarterly_cost_severe_PTSD)+CPT_90876*Number_NF_sessions_treatment*Percent_patients_NF_psychotherapy+Cost_drugs_quarterly*Percent_patients_NF_meds+CPT_90901*Number_NF_sessions_treatment*Percent_patients_NF_only+(CPT_90876*Number_NF_sessions_treatment+Cost_drugs_quarterly)*Percent_patients_NF_meds_psych+CPT_99214*(Percent_patients_NF_meds+Percent_patients_NF_meds_psych)*Probability_side_effects_pharmacotherapy
	Initial effectiveness; stage 0	If(Severe_PTSD_Quality_Life_quarterly+Per_CAPS_reduction_QOL_improvement*QoL_improvement_NF_plus_psychotherapy/4>=0.18;Moderate_PTSD_Quality_life_quarterly;Severe_PTSD_Quality_Life_quarterly)

Health state	Reward	Therapy state for NF+adjunctive
	Incremental effectiveness; states 1-11	If(Severe_PTSD_Quality_Life_quarterly+Per_CAPS_reduction_QOL_improvement*QoL_improvement_NF_plus_psychotherapy/4>=0.18;Moderate_PTSD_Quality_life_quarterly;Severe_PTSD_Quality_Life_quarterly)
	Final effectiveness; stage 12	If(Severe_PTSD_Quality_Life_quarterly+Per_CAPS_reduction_QOL_improvement*QoL_improvement_NF_plus_psychotherapy/4>=0.18;Moderate_PTSD_Quality_life_quarterly;Severe_PTSD_Quality_Life_quarterly)
Mild PTSD state	Initial cost; stage 0	0
	Incremental cost; stages 1-11	Quarterly_cost_mild_PTSD
	Final cost; stage 12	Quarterly_cost_mild_PTSD
	Initial effectiveness; stage 0	0
	Incremental effectiveness; states 1-11	Mild_PTSD_Quality_Life_quarterly
	Final effectiveness; stage 12	Mild_PTSD_Quality_Life_quarterly
Moderate PTSD state	Initial cost; stage 0	0
	Incremental cost; stages 1-11	Quarterly_cost_moderate_PTSD
	Final cost; stage 12	Quarterly_cost_moderate_PTSD
	Initial effectiveness; stage 0	0
	Incremental effectiveness; states 1-11	Quarterly_cost_moderate_PTSD
	Final effectiveness; stage 12	Quarterly_cost_moderate_PTSD
Severe PTSD state	Initial cost; stage 0	0
	Incremental cost; stages 1-11	Quarterly_cost_severe_PTSD
	Final cost; stage 12	Quarterly_cost_severe_PTSD
	Initial effectiveness; stage 0	0
	Incremental effectiveness; states 1-11	Severe_PTSD_Quality_Life_quarterly
	Final effectiveness; stage 12	Severe_PTSD_Quality_Life_quarterly
Dead	Initial cost; stage 0	0
	Incremental cost; stages 1-11	0
	Final cost; stage 12	0
	Initial effectiveness; stage 0	0

Health state	Reward	Therapy state for NF+adjunctive
	Incremental effectiveness; states 1-11	0
	Final effectiveness; stage 12	0
Relapse Mild PTSD	Initial cost; stage 0	0
	Incremental cost; stages 1-11	0
	Final cost; stage 12	0
	Initial effectiveness; stage 0	0
	Incremental effectiveness; states 1-11	0
	Final effectiveness; stage 12	0
Relapse Moderate PTSD	Initial cost; stage 0	0
	Incremental cost; stages 1-11	0
	Final cost; stage 12	0
	Initial effectiveness; stage 0	0
	Incremental effectiveness; states 1-11	0
	Final effectiveness; stage 12	0
Relapse Severe PTSD	Initial cost; stage 0	0
	Incremental cost; stages 1-11	0
	Final cost; stage 12	0
	Initial effectiveness; stage 0	0
	Incremental effectiveness; states 1-11	0
	Final effectiveness; stage 12	0

Health state	Reward	Therapy state for Pharmacotherpay
Less mild/asymptomatic	Initial cost; stage 0	Quarterly_cost_aysmptomatic_PTSD+Cost_drugs_quarterly+Cost_side_effect_drugs*Probability_side_effects_pharmacotherapy

	Incremental cost; stages 1-11	Quarterly_cost_aysmptomatic_PTSD+Cost_drugs_quarterly+Cost_side_effect_drugs*Probability_side_effects_pharmacotherapy
	Final cost; stage 12	Quarterly_cost_aysmptomatic_PTSD+Cost_drugs_quarterly+Cost_side_effect_drugs*Probability_side_effects_pharmacotherapy
	Initial effectiveness; stage 0	Asymptomatic_PTSD_QoL_quarterly
	Incremental effectiveness; states 1-11	Asymptomatic_PTSD_QoL_quarterly
	Final effectiveness; stage 12	Asymptomatic_PTSD_QoL_quarterly
Mild PTSD treatment	Initial cost; stage 0	If(Mild_PTSD_Quality_Life_quarterly+QoL_improvement_pharmacotherapy_treatment*Per_CAPS_reduction_QOL_improvement/4>=0.2375;Quarterly_cost_aysmptomatic_PTSD;Quarterly_cost_mild_PTSD)+Cost_drugs_quarterly+Cost_side_effect_drugs*Probability_side_effects_pharmacotherapy

	Incremental cost; stages 1-11	If(Mild_PTSD_Quality_Life_quarterly+QoL_improvement_pharmacotherapy_treatment*Per_CAPS_reduction_QOL_improvement/4>=0.2375;Quarterly_cost_aysmptomatic_PTSD;Quarterly_cost_mild_PTSD)+Cost_drugs_quarterly+Cost_side_effect_drugs*Probability_side_effects_pharmacotherapy
	Final cost; stage 12	If(Mild_PTSD_Quality_Life_quarterly+QoL_improvement_pharmacotherapy_treatment*Per_CAPS_reduction_QOL_improvement/4>=0.2375;Quarterly_cost_aysmptomatic_PTSD;Quarterly_cost_mild_PTSD)+Cost_drugs_quarterly+Cost_side_effect_drugs*Probability_side_effects_pharmacotherapy
	Initial effectiveness; stage 0	If(Mild_PTSD_Quality_Life_quarterly+QoL_improvement_pharmacotherapy_treatment*Per_CAPS_reduction_QOL_improvement/4>=0.2375;Asymptomatic_PTSD_QoL_quarterly;Mild_PTSD_Quality_Life_quarterly)

	Incremental effectiveness; states 1-11	If(Mild_PTSD_Quality_Life_quarterly+QoL_improvement_pharmacotherapy_treatment*Per_CAPS_reduction_QOL_improvement/4>=0.2375;Asymptomatic_PTSD_QoL_quarterly;Mild_PTSD_Quality_Life_quarterly)
	Final effectiveness; stage 12	If(Mild_PTSD_Quality_Life_quarterly+QoL_improvement_pharmacotherapy_treatment*Per_CAPS_reduction_QOL_improvement/4>=0.2375;Asymptomatic_PTSD_QoL_quarterly;Mild_PTSD_Quality_Life_quarterly)
Moderate PTSD treatment	Initial cost; stage 0	If(Moderate_PTSD_Quality_life_quarterly+Per_CAPS_reduction_QOL_improvement*QoL_improvement_pharmacotherapy_treatment/4>=0.2075;Quarterly_cost_mild_PTSD;Quarterly_cost_moderate_PTSD)+Cost_drugs_quarterly+Cost_side_effect_drugs*Probability_side_effects_pharmacotherapy

	Incremental cost; stages 1-11	If(Moderate_PTSD_Quality_life_quarterly+Per_CAPS_reduction_QOL_improvement*QoL_improvement_pharmacotherapy_treatment/4>=0.2075;Quarterly_cost_mild_PTSD;Quarterly_cost_moderate_PTSD)+Cost_drugs_quarterly+Cost_side_effect_drugs*Probability_side_effects_pharmacotherapy
	Final cost; stage 12	If(Moderate_PTSD_Quality_life_quarterly+Per_CAPS_reduction_QOL_improvement*QoL_improvement_pharmacotherapy_treatment/4>=0.2075;Quarterly_cost_mild_PTSD;Quarterly_cost_moderate_PTSD)+Cost_drugs_quarterly+Cost_side_effect_drugs*Probability_side_effects_pharmacotherapy
	Initial effectiveness; stage 0	If(Moderate_PTSD_Quality_life_quarterly+Per_CAPS_reduction_QOL_improvement*QoL_improvement_pharmacotherapy_treatment/4>=0.2075;Mild_PTSD_Quality_Life_quarterly;Moderate_PTSD_Quality_life_quarterly)

	Incremental effectiveness; states 1-11	If(Moderate_PTSD_Quality_life_quarterly+Per_CAPS_reduction_QOL_improvement*QoL_improvement_pharmacotherapy_treatment/4>=0.2075;Mild_PTSD_Quality_Life_quarterly;Moderate_PTSD_Quality_life_quarterly)
	Final effectiveness; stage 12	If(Moderate_PTSD_Quality_life_quarterly+Per_CAPS_reduction_QOL_improvement*QoL_improvement_pharmacotherapy_treatment/4>=0.2075;Mild_PTSD_Quality_Life_quarterly;Moderate_PTSD_Quality_life_quarterly)
Severe PTSD treatment	Initial cost; stage 0	If(Severe_PTSD_Quality_Life_quarterly+Per_CAPS_reduction_QOL_improvement*QoL_improvement_pharmacotherapy_treatment/4>=0.18;Quarterly_cost_moderate_PTSD;Quarterly_cost_severe_PTSD)+Cost_drugs_quarterly+Cost_side_effect_drugs*Probability_side_effects_pharmacotherapy

	Incremental cost; stages 1-11	If(Severe_PTSD_Quality_Life_quarterly+Per_CAPS_reduction_QOL_improvement*QoL_improvement_pharmacotherapy_treatment/4>=0.18;Quarterly_cost_moderate_PTSD;Quarterly_cost_severe_PTSD)+Cost_drugs_quarterly+Cost_side_effect_drugs*Probability_side_effects_pharmacotherapy
	Final cost; stage 12	If(Severe_PTSD_Quality_Life_quarterly+Per_CAPS_reduction_QOL_improvement*QoL_improvement_pharmacotherapy_treatment/4>=0.18;Quarterly_cost_moderate_PTSD;Quarterly_cost_severe_PTSD)+Cost_drugs_quarterly+Cost_side_effect_drugs*Probability_side_effects_pharmacotherapy
	Initial effectiveness; stage 0	If(Severe_PTSD_Quality_Life_quarterly+Per_CAPS_reduction_QOL_improvement*QoL_improvement_pharmacotherapy_treatment/4>=0.18;Moderate_PTSD_Quality_life_quarterly;Severe_PTSD_Quality_Life_quarterly)

	Incremental effectiveness; states 1-11	If(Severe_PTSD_Quality_Life_quarterly+Per_CAPS_reduction_QOL_improvement*QoL_improvement_pharmacotherapy_treatment/4>=0.18;Moderate_PTSD_Quality_life_quarterly;Severe_PTSD_Quality_Life_quarterly)
	Final effectiveness; stage 12	If(Severe_PTSD_Quality_Life_quarterly+Per_CAPS_reduction_QOL_improvement*QoL_improvement_pharmacotherapy_treatment/4>=0.18;Moderate_PTSD_Quality_life_quarterly;Severe_PTSD_Quality_Life_quarterly)
Mild PTSD state	Initial cost; stage 0	0
	Incremental cost; stages 1-11	Quarterly_cost_mild_PTSD
	Final cost; stage 12	Quarterly_cost_mild_PTSD
	Initial effectiveness; stage 0	0
	Incremental effectiveness; states 1-11	Mild_PTSD_Quality_Life_quarterly
	Final effectiveness; stage 12	Mild_PTSD_Quality_Life_quarterly
Moderate PTSD state	Initial cost; stage 0	0
	Incremental cost; stages 1-11	Quarterly_cost_moderate_PTSD
	Final cost; stage 12	Quarterly_cost_moderate_PTSD
	Initial effectiveness; stage 0	0

	Incremental effectiveness; states 1-11	Moderate_PTSD_Quality_life_quarterly
	Final effectiveness; stage 12	Moderate_PTSD_Quality_life_quarterly
Severe PTSD state	Initial cost; stage 0	0
	Incremental cost; stages 1-11	Quarterly_cost_severe_PTSD
	Final cost; stage 12	Quarterly_cost_severe_PTSD
	Initial effectiveness; stage 0	0
	Incremental effectiveness; states 1-11	Severe_PTSD_Quality_Life_quarterly
	Final effectiveness; stage 12	Severe_PTSD_Quality_Life_quarterly
Dead	Initial cost; stage 0	0
	Incremental cost; stages 1-11	0
	Final cost; stage 12	0
	Initial effectiveness; stage 0	0
	Incremental effectiveness; states 1-11	0
	Final effectiveness; stage 12	0
Relapse Mild PTSD	Initial cost; stage 0	0
	Incremental cost; stages 1-11	0
	Final cost; stage 12	0
	Initial effectiveness; stage 0	0
	Incremental effectiveness; states 1-11	0
	Final effectiveness; stage 12	0
Relapse Moderate PTSD	Initial cost; stage 0	0
	Incremental cost; stages 1-11	0
	Final cost; stage 12	0
	Initial effectiveness; stage 0	0
	Incremental effectiveness; states 1-11	0

	Final effectiveness; stage 12	0
Relapse Severe PTSD	Initial cost; stage 0	0
	Incremental cost; stages 1-11	0
	Final cost; stage 12	0
	Initial effectiveness; stage 0	0
	Incremental effectiveness; states 1-11	0
	Final effectiveness; stage 12	0

Supplementary S7

CHEERS 2022 Checklist

	Item	Guidance for Reporting	Reported in section
**TITLE**			
Title	1	Identify the study as an economic evaluation and specify the interventions being compared.	1
**ABSTRACT**			
Abstract	2	Provide a structured summary that highlights context, key methods, results and alternative analyses.	2
**INTRODUCTION**			
Background and objectives	3	Give the context for the study, the study question and its practical relevance for decision making in policy or practice.	3-4
**METHODS**			
Health economic analysis plan	4	Indicate whether a health economic analysis plan was developed and where available.	5
Study population	5	Describe characteristics of the study population (such as age range, demographics, socioeconomic, or clinical characteristics).	5
Setting and location	6	Provide relevant contextual information that may influence findings.	5
Comparators	7	Describe the interventions or strategies being compared and why chosen.	5
Perspective	8	State the perspective(s) adopted by the study and why chosen.	5
Time horizon	9	State the time horizon for the study and why appropriate.	5
Discount rate	10	Report the discount rate(s) and reason chosen.	5
Selection of outcomes	11	Describe what outcomes were used as the measure(s) of benefit(s) and harm(s).	5
Measurement of outcomes	12	Describe how outcomes used to capture benefit(s) and harm(s) were measured.	5
Valuation of outcomes	13	Describe the population and methods used to measure and value outcomes.	5
Measurement and valuation of resources and costs	14	Describe how costs were valued.	5
Currency, price date, and conversion	15	Report the dates of the estimated resource quantities and unit costs, plus the currency and year of conversion.	5
Rationale and description of model	16	If modelling is used, describe in detail and why used. Report if the model is publicly available and where it can be accessed.	5
Analytics and assumptions	17	Describe any methods for analysing or statistically transforming data, any extrapolation methods, and approaches for validating any model used.	5
Characterizing heterogeneity	18	Describe any methods used for estimating how the results of the study vary for sub-groups.	N/A
Characterizing distributional effects	19	Describe how impacts are distributed across different individuals or adjustments made to reflect priority populations.	8
Characterizing uncertainty	20	Describe methods to characterize any sources of uncertainty in the analysis.	5
Approach to engagement with patients and others affected by the study	21	Describe any approaches to engage patients or service recipients, the general public, communities, or stakeholders (e.g., clinicians or payers) in the design of the study.	N/A
**RESULTS**			
Study parameters	22	Report all analytic inputs (e.g., values, ranges, references) including uncertainty or distributional assumptions.	eAppendix 3,5
Summary of main results	23	Report the mean values for the main categories of costs and outcomes of interest and summarise them in the most appropriate overall measure.	6,7

Effect of uncertainty	24	Describe how uncertainty about analytic judgments, inputs, or projections affect findings. Report the effect of choice of discount rate and time horizon, if applicable.	6,7
Effect of engagement with patients and others affected by the study	25	Report on any difference patient/service recipient, general public, community, or stakeholder involvement made to the approach or findings of the study	8
**DISCUSSION**			
Study findings, limitations, generalizability, and current knowledge	26	Report key findings, limitations, ethical or equity considerations not captured, and how these could impact patients, policy, or practice.	8,9
**OTHER RELEVANT INFORMATION**			
Source of funding	27	Describe how the study was funded and any role of the funder in the identification, design, conduct, and reporting of the analysis	9
Conflicts of interest	28	Report authors conflicts of interest according to journal or International Committee of Medical Journal Editors requirements.	9

Husereau D, Drummond M, Augustovski F, de Bekker-Grob E, Briggs AH, Carswell C, Caulley L, Chaiyakunapruk N, Greenberg D, Loder E, Mauskopf J, Mullins CD, Petrou S, Pwu RF, Staniszewska S; CHEERS 2022 ISPOR Good Research Practices Task Force. Consolidated Health Economic Evaluation Reporting Standards 2022 (CHEERS 2022) Statement: Updated Reporting Guidance for Health Economic Evaluations. BMJ. 2022;376:e067975.

The checklist is Open Access distributed in accordance with the terms of the Creative Commons Attribution (CC BY 4.0) license, which permits others to distribute, remix, adapt and build upon this work, for commercial use, provided the original work is properly cited. See:

http://creativecommons.org/licenses/by/4.0/

Supplementary S8: Systematic reviews and meta-analysis used in the cost-effectiveness analysis – methodology used
18

Study	Design	Method of extraction of data	Data extracted
Williams T, Phillips NJ, Stein DJ, et al. Pharmacotherapy for post-traumatic stress disorder (PTSD). *Cochrane Database of Systematic Reviews* Issue 3. 2022. Art. No.: CD002795.	Review of all randomized controlled trials (RCTs) on use of pharmacotherapy in treating PTSD.	Three review authors (TW, JI, and NP) independently assessed RCTs for inclusion in the review, collated trial data, and assessed trial quality. Contacted investigators to obtain missing data. We stratified summary statistics by medication class, and by medication agent for all medications. Calculated dichotomous and continuous measures using a random-effects model, and assessed heterogeneity.	Sixty six RCT's were identified. Dropout rates of 33% were identified.
Lewis C, Roberts NP, Gibson S, et al. Dropout from psychological therapies for PTSD in adults: A systematic review and meta-analysis. *Eur. Jrl. Psychotraum*. 2020 ;11,1709709.	Review of all RCT's on use of psychotherapy in treating PTSD which analyzed dropout rates.	A search was conducted by the Cochrane Collaboration. At least 70% of study participants were required to be diagnosed with PTSD with a duration of three months or more. Study characteristics and dropout data were extracted by two reviewers independently and in duplicate, using a form that had been pre-piloted. The pooled rate of dropout from psychological therapies was estimated.	One hundred fifteen RCT's identified with 7,724 patients. Dropout rates from RCTs of psychological therapies for PTSD was 16%<br>(95% CI 14–18%).
Weber M, Schumacher S, Hannig W, et al. Long-term outcomes of psychological treatment for post-traumatic stress disorder: a systematic review and meta-analysis. *Psych Med*. 2021;51:1420-1430.	Review of all RCTs on use of psychotherapy in treating PTSD which analyzed dropout rates.	Preferred Reporting Items for Systematic Reviews and Meta-Analyses (PRISMA) statement for conducting and reporting this meta-analysis	Studies were published between 1999 and 2018 and comprised 28–353 participants per study sample (N = 2638); included 22 studies in the meta-analysis. Use of 24% dropout rate.
Voigt JD, Mosier M, Tendler A. Systematic review and meta-analysis of neurofeedback and its effect on post-traumatic stress disorder. *Front Psych*. 2024;15:doi: 10.3389/fpsyt.2024.132348	Review of all RCTs on use of NF as an adjunctive therapy in treating PTSD.	Search conducted by authors and adjudicated by consensus. PRISMA statement used for conducting and reporting on this meta-analysis.	Ten studies used in meta-analysis. Total of 628 patients evaluated. CAPS-5 pre-test/post-test analysis used. Pooled dropout rate of 13.2%
Ipser JC, Stein DJ. Evidence-based pharmacotherapy of post-traumatic stress disorder (PTSD). *Int Jr. Neuropsychopharma.* 2012;15:825-840.	Review of placebo-controlled RCTs of pharmacotherapy for adults diagnosed with PTSD.	Eligible RCTs were identified in February, 2010 by systematically searching the following databases: Medline, EMBASE, the Cochrane Central Register of Controlled Trials, the Cochrane Collaboration Depression, Anxiety and Neurosis Controlled Trials Register, and the National PTSD Center PILOTS database.	CAPS-5 reduction of 6.64 (95% CI: 4.16 to 9.11) with SSRI's; use of 13 studies and 2,642 patients.
Morina N, Hoppen TH, Kip A. Study quality and efficacy of psychological interventions for	RCTs of psychotherapy vs. control (commonly waiting list)	Studies included only evaluated CAPS-5 scores pre and at last follow-up. The aims	32 trials identified with 4,300 patients; with intent to treat (ITT) used when

Study	Design	Method of extraction of data	Data extracted
posttraumatic stress disorder: a meta-analysis of randomized controlled trials. *Psychological Medicine* 2021; 51, 1260–1270. https://doi.org/10.1017/S0033291721001641	that evaluated patients on CAPS-5 improvement over time.	and methods of this meta-analysis were registered with the PROSPERO database (CRD42018094698, http://www.crd.york.ac.uk/prospero).	available. Follow up of 3-12 months post therapy. Hedge's g of 1.21 (95% C: 0.95 to 1.45) converted to mean difference for CAPs-5; 5.98±4.94.
Levy HC, O'Bryan EM, Tolin DF. A meta-analysis of relapse rates in CBT for anxiety disorders. *Jrl Anx Dis.* 2021;81:102407	RCTs of psychotherapy for treatment of PTSD.	The primary outcome variable was the number of relapsed patients at follow-up; with an average of 9 months of follow-up.	17 full-length articles retained for meta-analysis (total *N* = 337 patients). Use of relapse data of 14% for psychotherapy.

Supplementary S9: Adjustment of CAPS-5 scores in psychotherapy to account for differences in study populations being compared

Assumes a CAPS-5 degradation of combat of 30% vs. civilians[1]

Study	CAPS -5	Civilians	Combat	Calc caps civilian	Calc cap combat	total
Psych Med - psychotherapy[2]	5·98	0·735	0·265	6·5	4·55	5·98325
Front Psych - NF[3]	7·01	0·87	0·13	7·3	5·11	7·0153
Adjusting psychotherapy for the same breakout as NF						
Psych Med - psychother	5·98	0·87	0·13	6·5	4·55	6·2465

1. Scnurr PP, PTSD treatment response in military populations. PTSD quarterly research. 2023;34(1): ISSN:1050-835.

2 Weber M, Schumacher S, Hannig W, et al. Long-term outcomes of psychological treatment for post-traumatic stress disorder: a systematic review and meta-analysis. Psych Med. 2021;51:1420-1430.

3 Voigt JD, Mosier M, Tendler A. Systematic review and meta-analysis of neurofeedback and its effect on post-traumatic stress disorder. Front Psych. 2024;15:doi: 10.3389/fpsyt.2024.132348

The psychotherapy group was comprised of 26.5 percent of combat veterans vs. 13 percent for NF+OT. An adjustment was therefore made in the psychotherapy group to reflect the same breakout as in NT+OT group (87 percent civilian and 13 percent military). The assumption was also made that there was a 30 percent degradation in the civilian CAPS-5 scores from 6.5 for military to 4.55 (6.5 X 0.7 = 4.55) which then was then used to comprise 13 percent of the overall CAPS-5 score based on a 87/13 civilian/ military percent breakout for NF+OT. Thus the CAPS-5 scores for psychotherapy were adjusted to 6.25 (6.5 X 0.87 + 6.5 X 0.7 X 0.13).

Figure S1

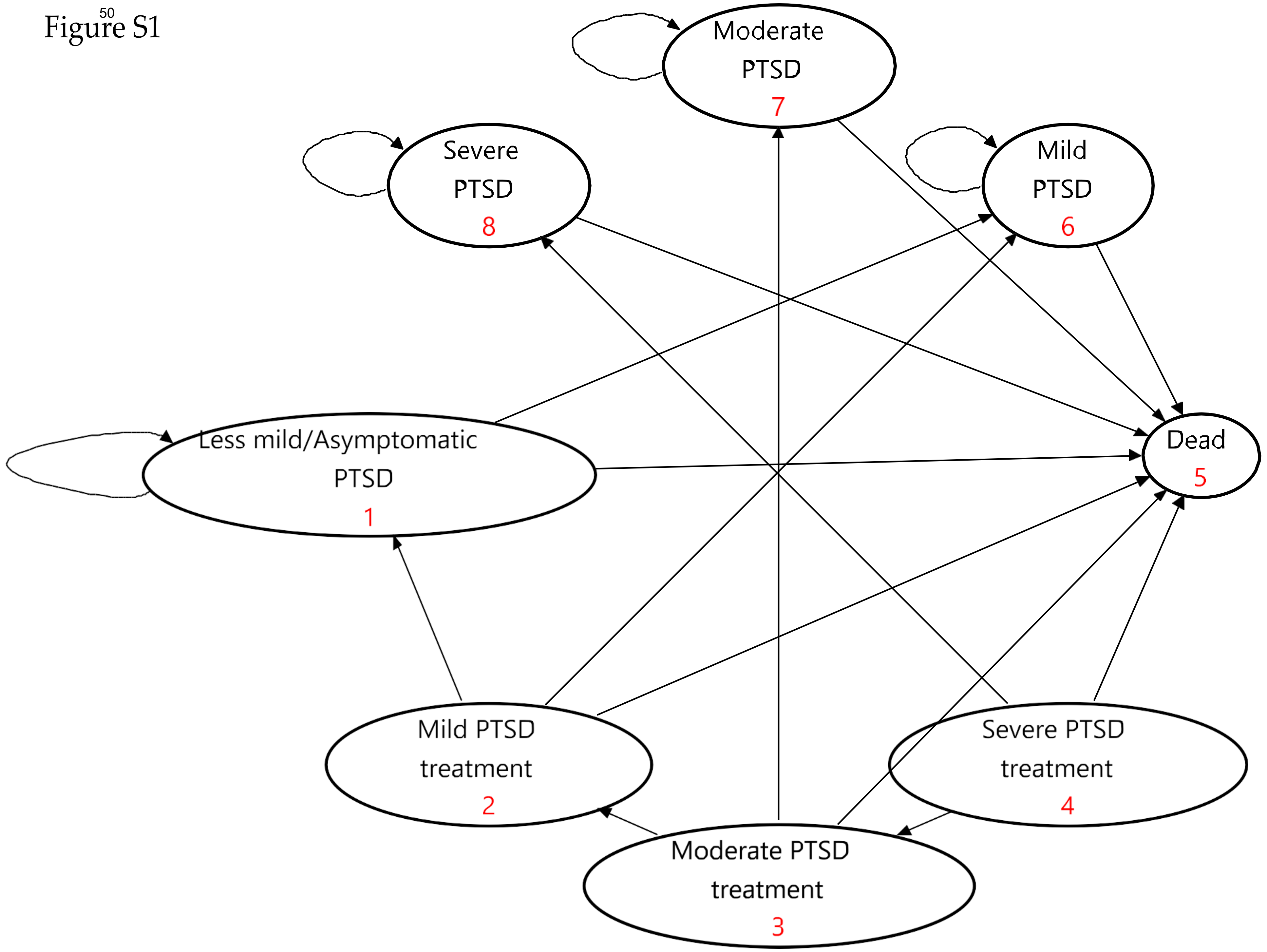

Figure S1 state transition diagram for NF+OT Psychotherapy, and Pharmacotherapy

Figure S2- Markov model

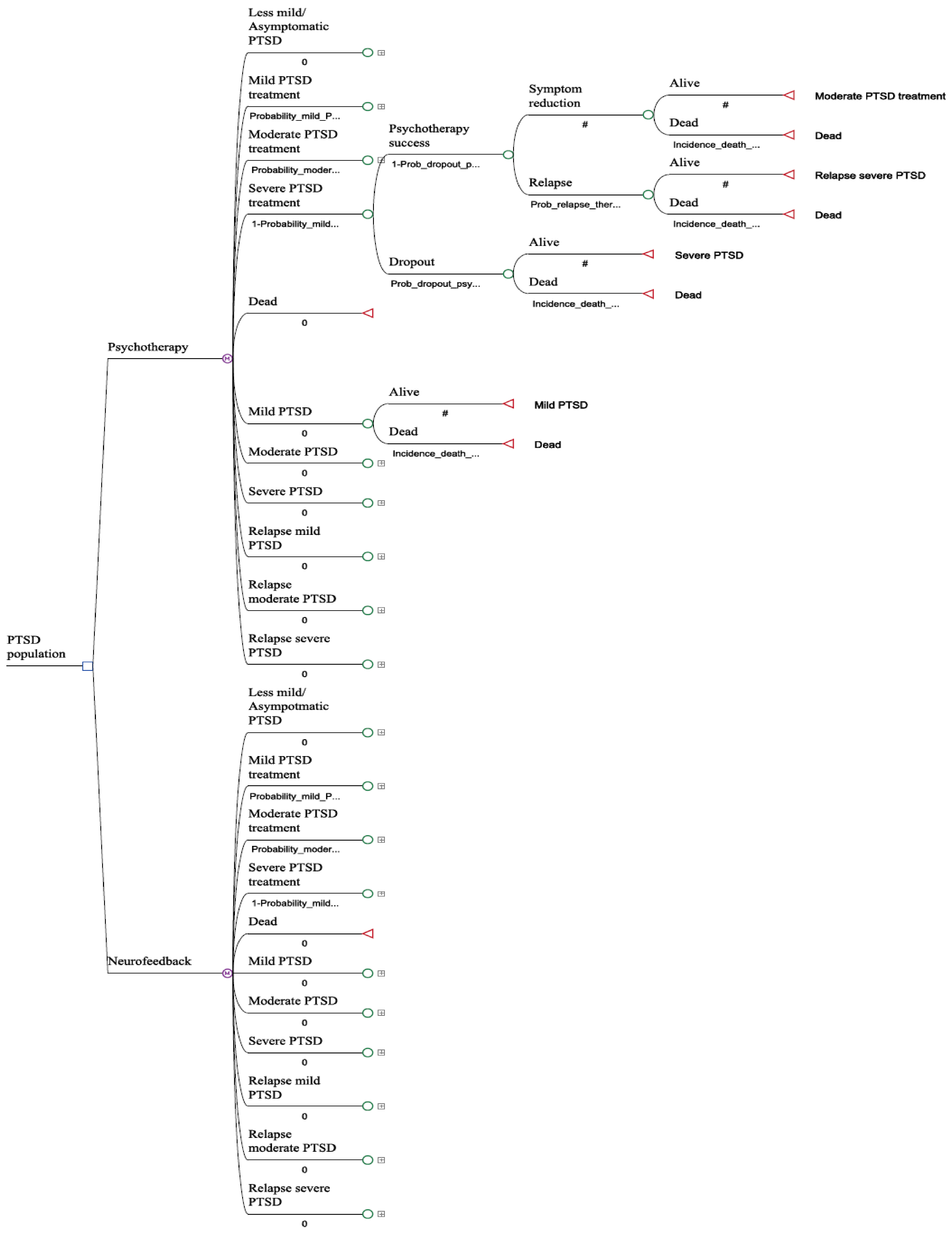

Figure S3

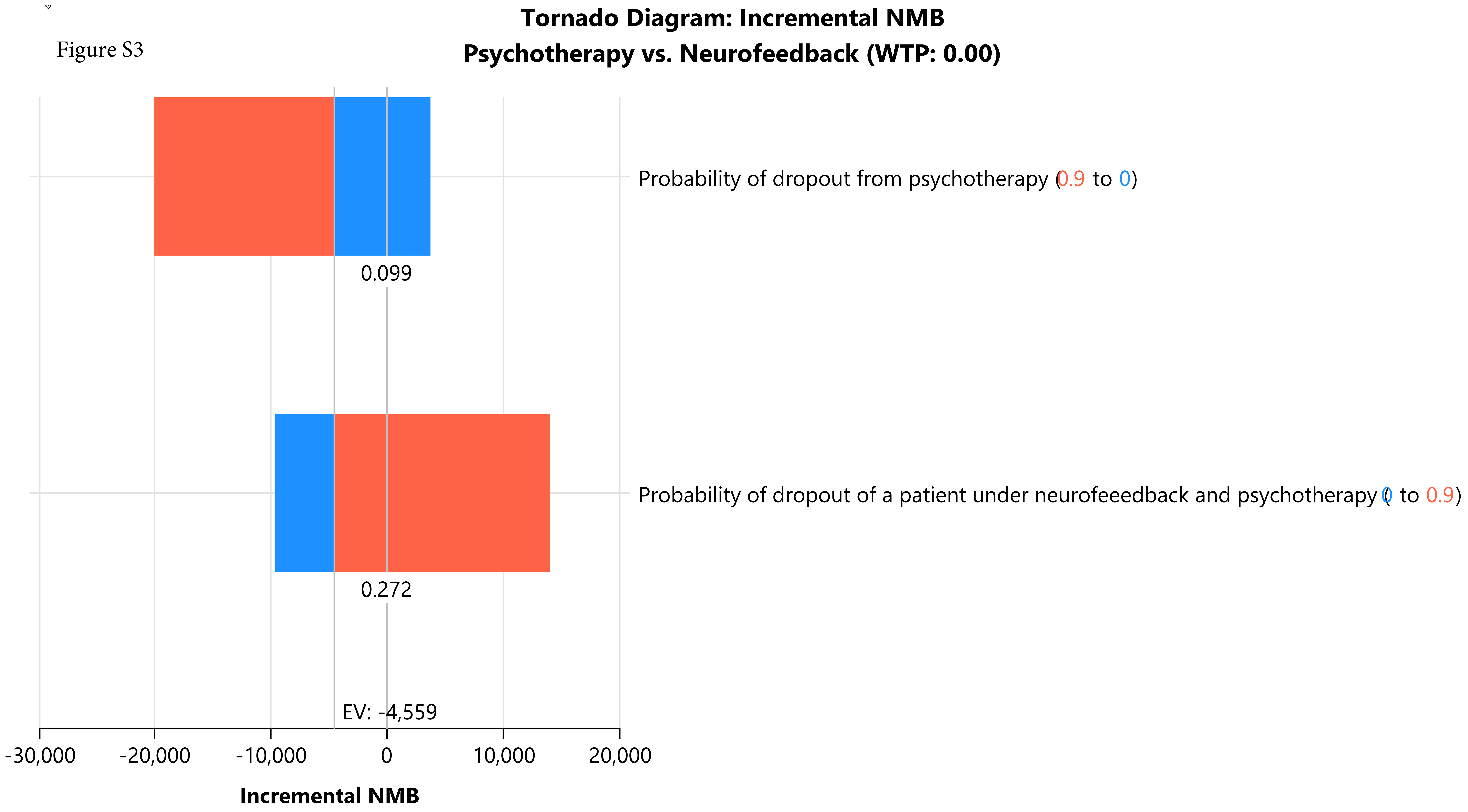
Tornado Diagram: Incremental NMB
Psychotherapy vs. Neurofeedback (WTP: 0.00)
Probability of dropout from psychotherapy (0.9 to 0)
0.099
Probability of dropout of a patient under neurofeeedback and psychotherapy (0 to 0.9)
0.272
EV: -4,559
-30,000
-20,000
-10,000
0
10,000
20,000
Incremental NMB

Figure S4

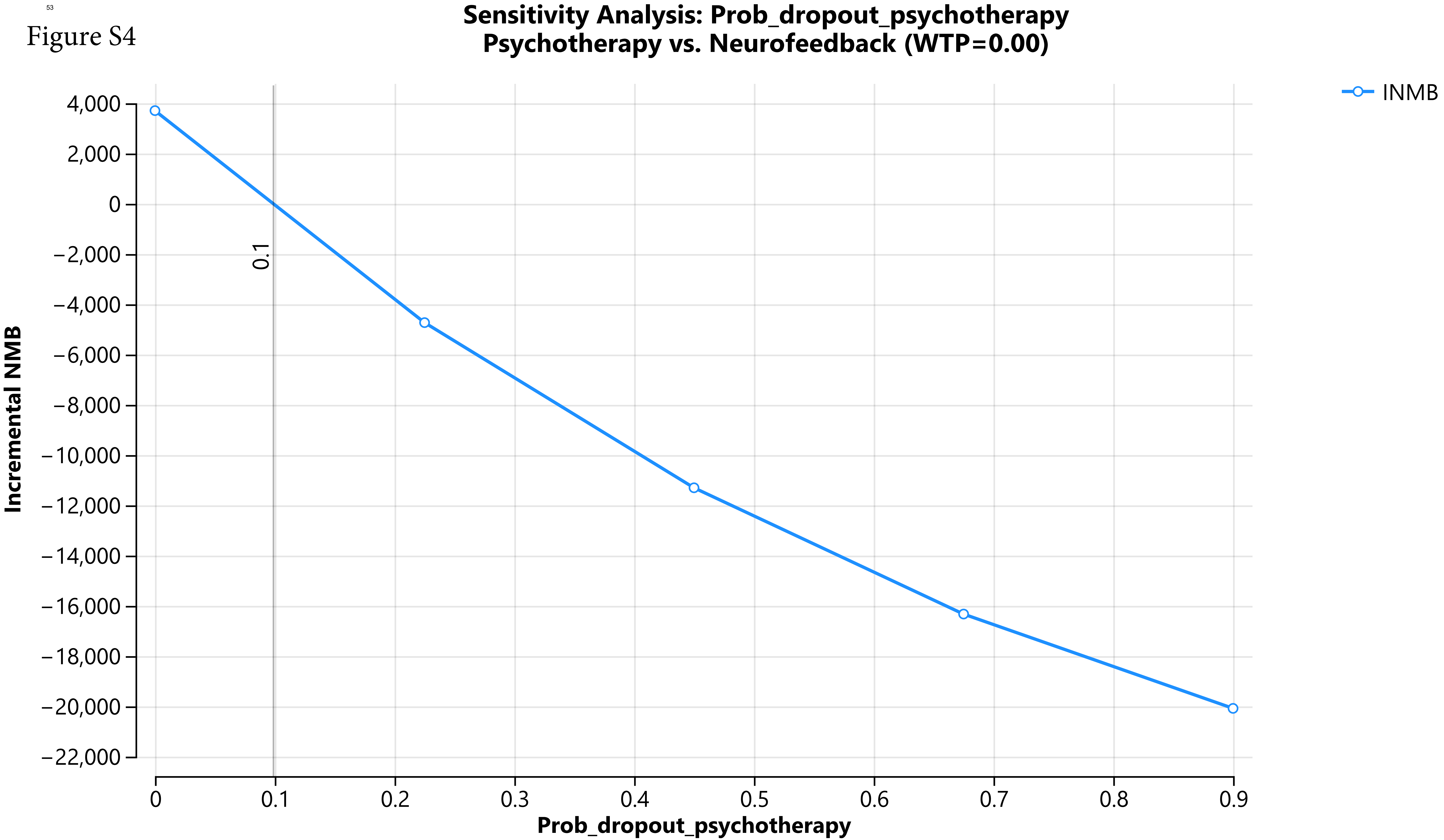
Sensitivity Analysis: Prob_dropout_psychotherapy
Psychotherapy vs. Neurofeedback (WTP=0.00)
INMB
Incremental NMB
4,000
2,000
0
−2,000
−4,000
−6,000
−8,000
−10,000
−12,000
−14,000
−16,000
−18,000
−20,000
−22,000
0.1
0
0.1
0.2
0.3
0.4
0.5
0.6
0.7
0.8
0.9
Prob_dropout_psychotherapy

Figure S5

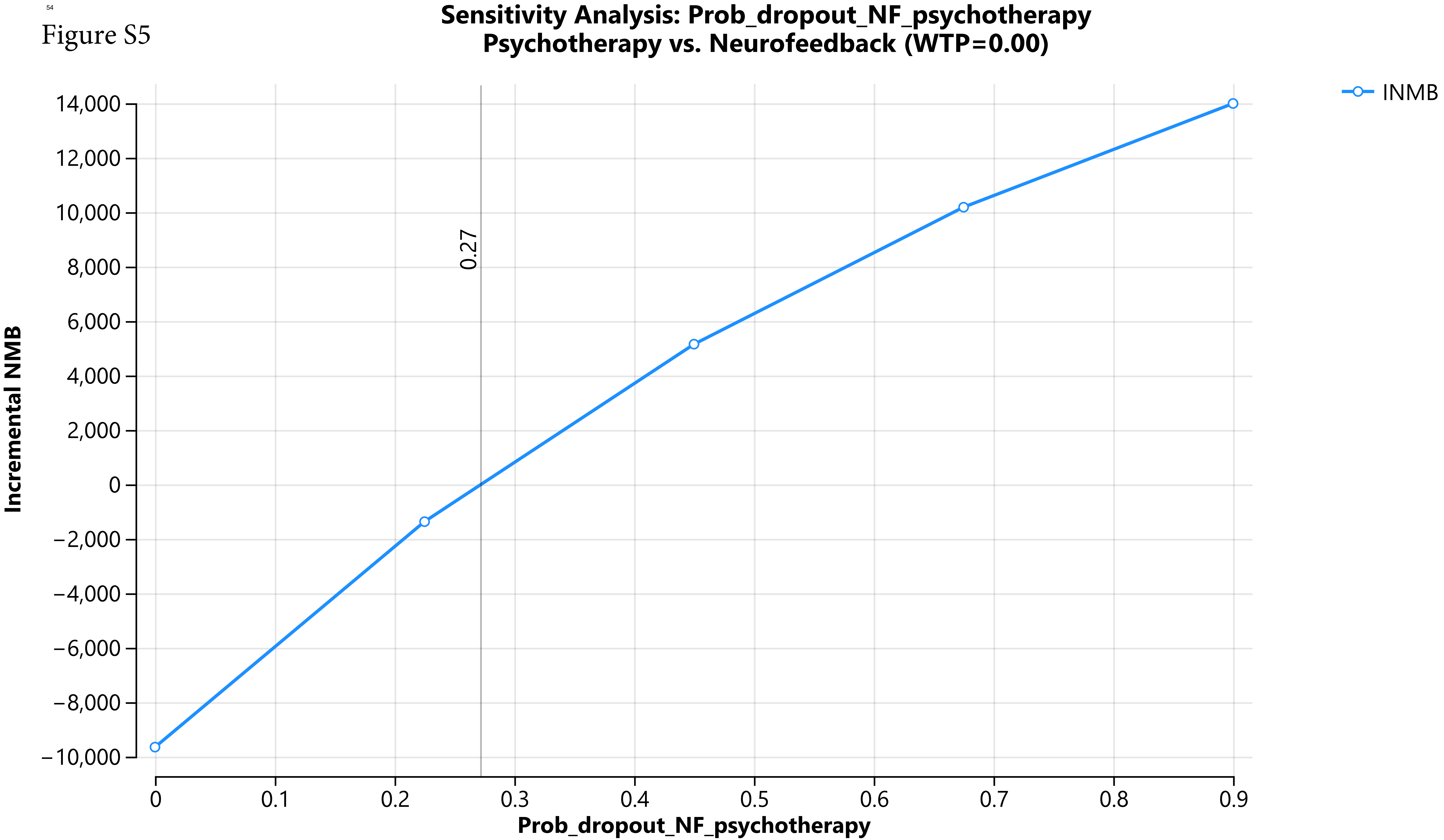
Sensitivity Analysis: Prob_dropout_NF_psychotherapy
Psychotherapy vs. Neurofeedback (WTP=0.00)
INMB
Incremental NMB
14,000
12,000
10,000
8,000
6,000
4,000
2,000
0
−2,000
−4,000
−6,000
−8,000
−10,000
0.27
0
0.1
0.2
0.3
0.4
0.5
0.6
0.7
0.8
0.9
Prob_dropout_NF_psychotherapy

Figure S6
Markov Model

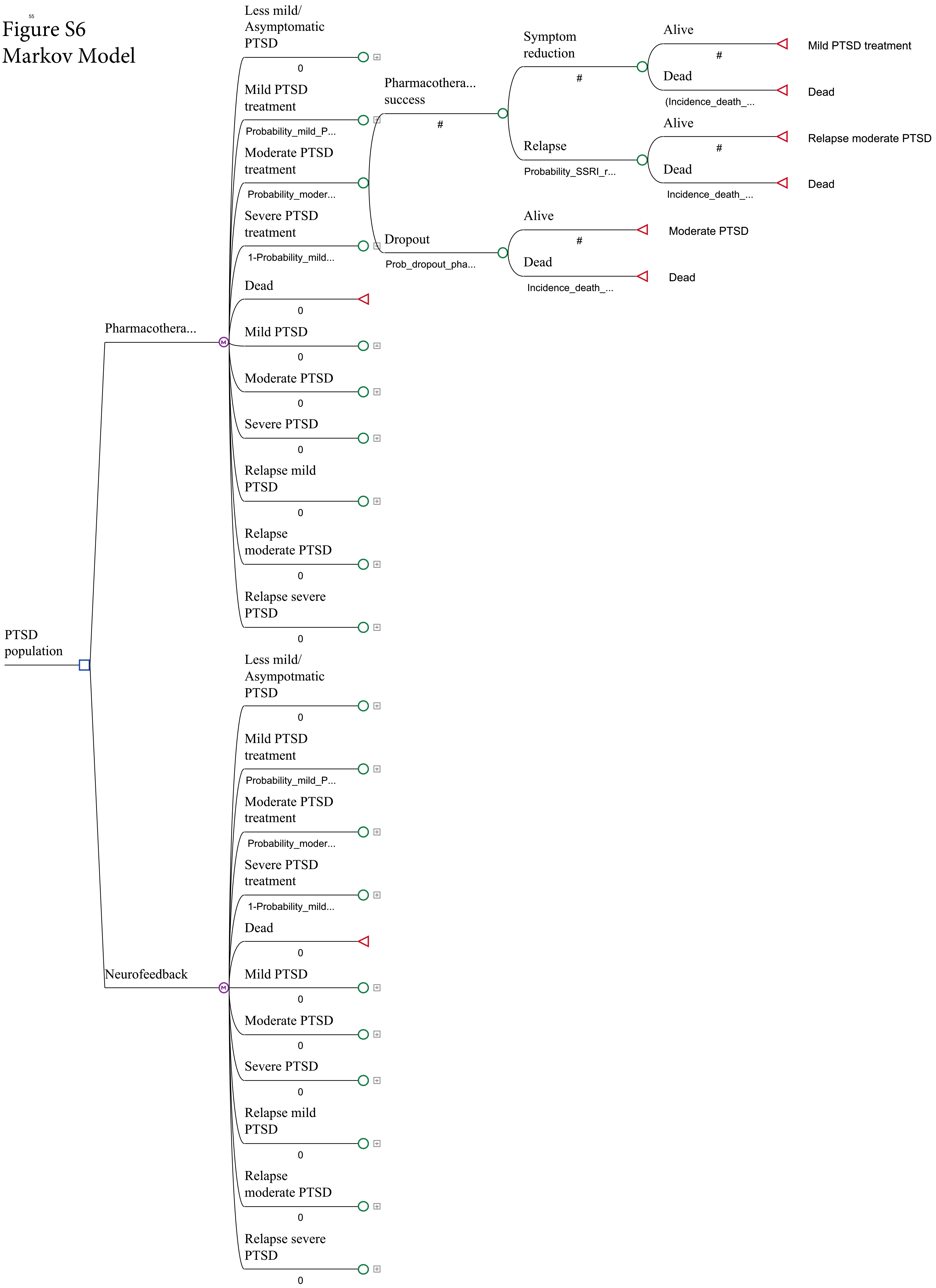

Figure S7

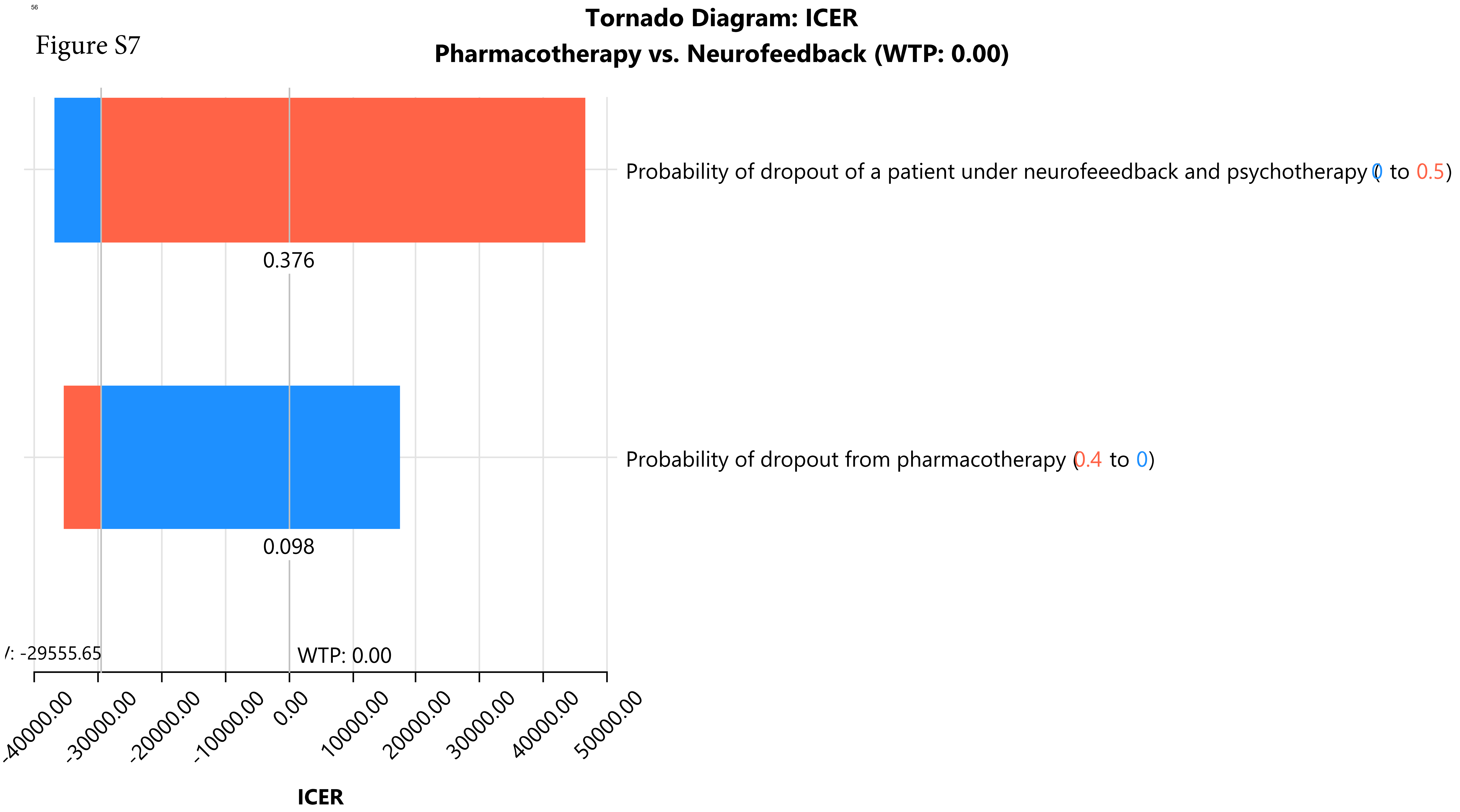
Tornado Diagram: ICER
Pharmacotherapy vs. Neurofeedback (WTP: 0.00)
Probability of dropout of a patient under neurofeeedback and psychotherapy (0 to 0.5)
0.376
Probability of dropout from pharmacotherapy (0.4 to 0)
0.098
: -29555.65
WTP: 0.00
-40000.00
-30000.00
-20000.00
-10000.00
0.00
10000.00
20000.00
30000.00
40000.00
50000.00
ICER

Figure S8

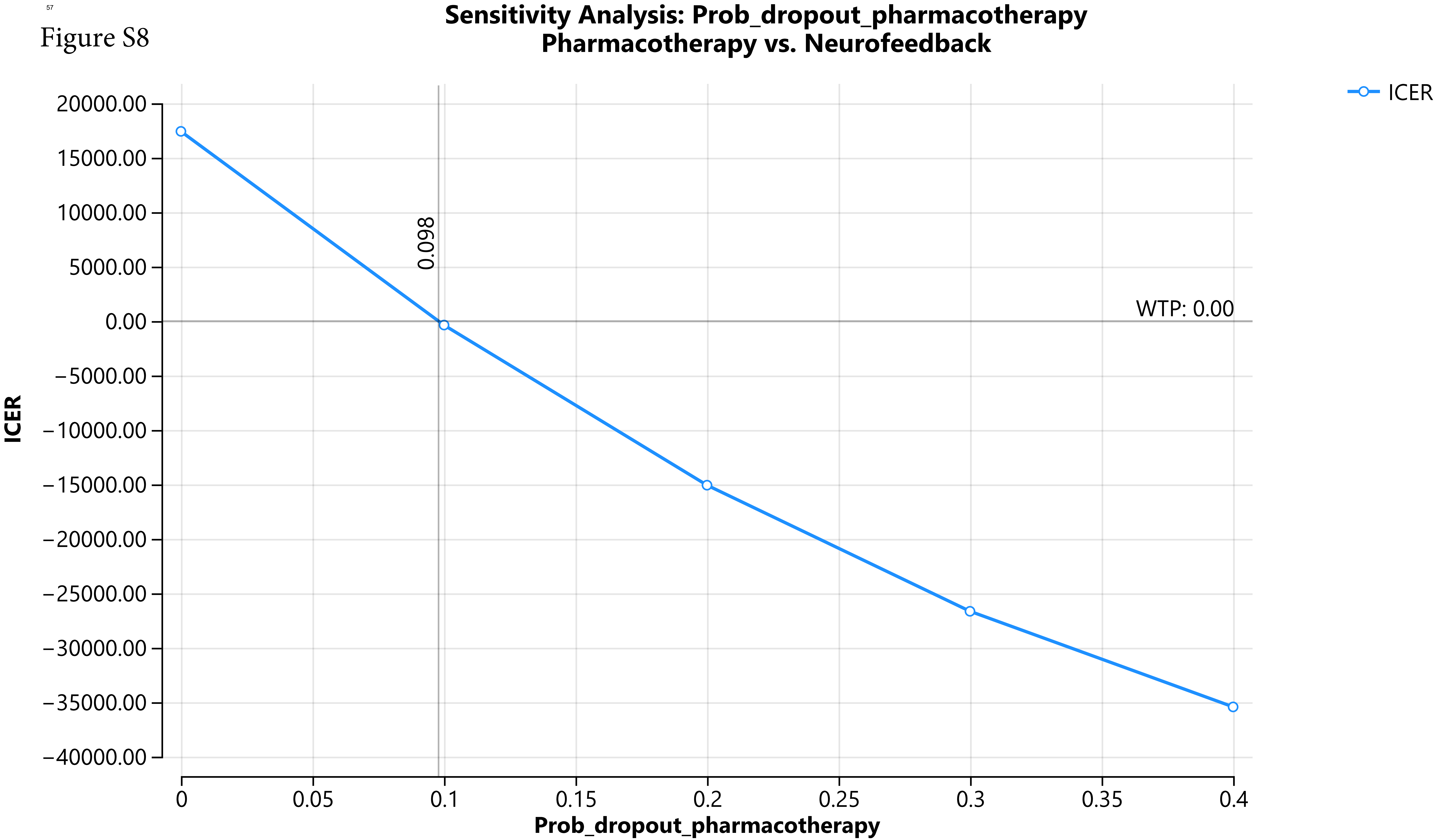
Sensitivity Analysis: Prob_dropout_pharmacotherapy
Pharmacotherapy vs. Neurofeedback
ICER
20000.00
15000.00
10000.00
5000.00
0.00
−5000.00
−10000.00
−15000.00
−20000.00
−25000.00
−30000.00
−35000.00
−40000.00
ICER
0.098
WTP: 0.00
0
0.05
0.1
0.15
0.2
0.25
0.3
0.35
0.4
Prob_dropout_pharmacotherapy

Figure S9

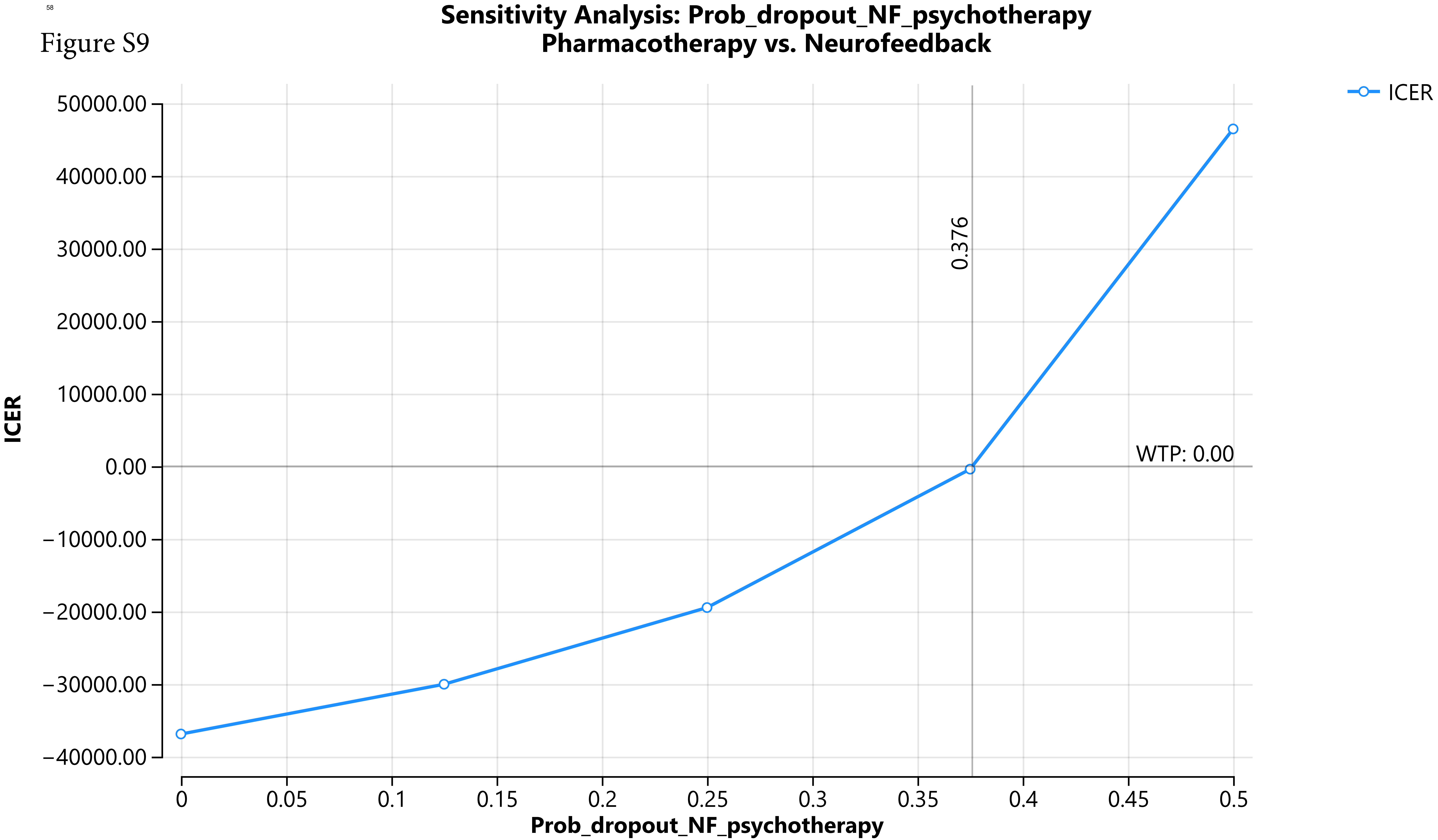
Sensitivity Analysis: Prob_dropout_NF_psychotherapy
Pharmacotherapy vs. Neurofeedback
ICER
50000.00
40000.00
30000.00
20000.00
10000.00
0.00
−10000.00
−20000.00
−30000.00
−40000.00
0.376
WTP: 0.00
0
0.05
0.1
0.15
0.2
0.25
0.3
0.35
0.4
0.45
0.5
Prob_dropout_NF_psychotherapy

Figure S10

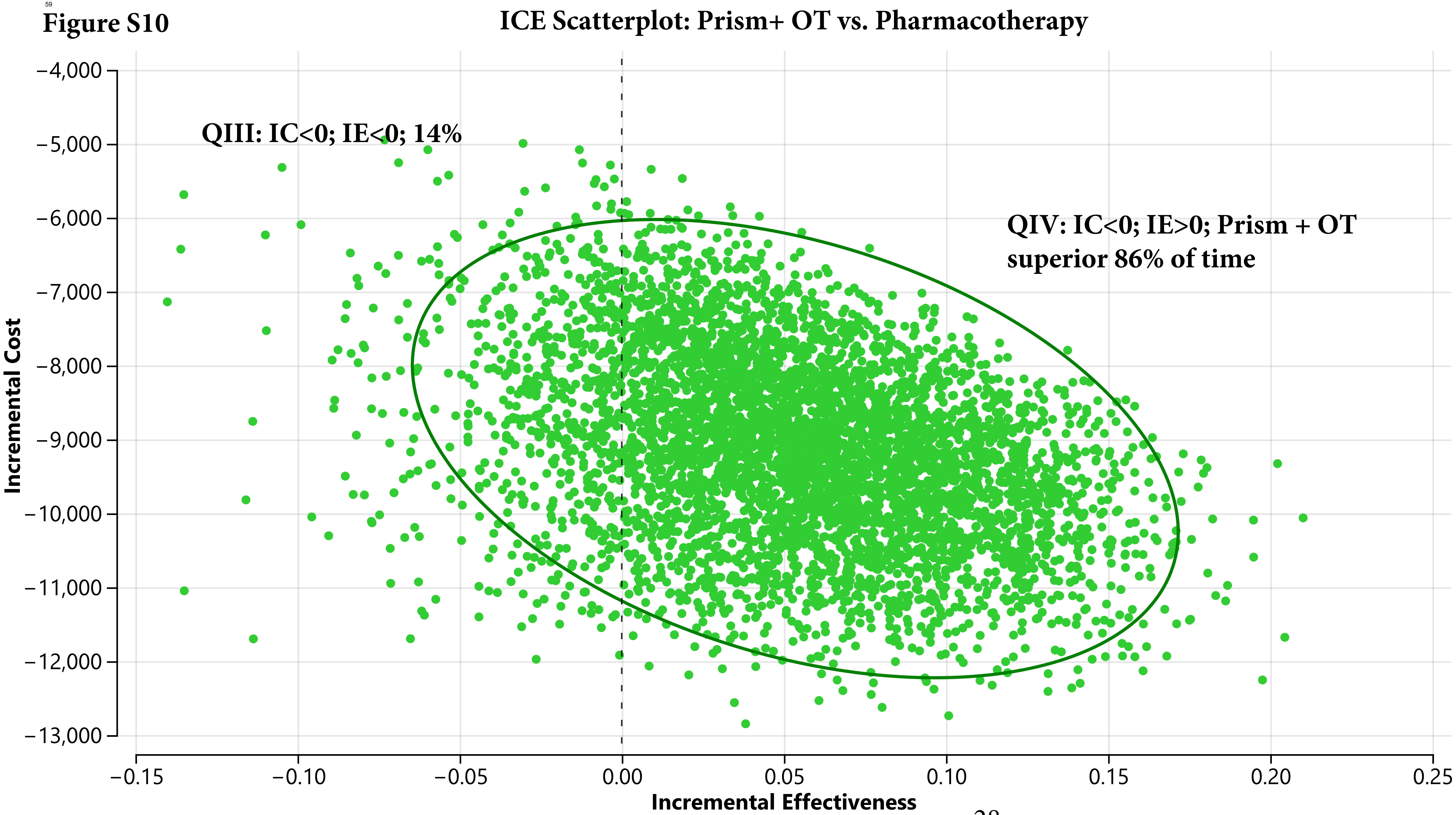
ICE Scatterplot: Prism+ OT vs. Pharmacotherapy
QIII: IC<0; IE<0; 14%
QIV: IC<0; IE>0; Prism + OT superior 86% of time
Incremental Cost
Incremental Effectiveness
−4,000
−5,000
−6,000
−7,000
−8,000
−9,000
−10,000
−11,000
−12,000
−13,000
−0.15
−0.10
−0.05
0.00
0.05
0.10
0.15
0.20
0.25

Figure S11

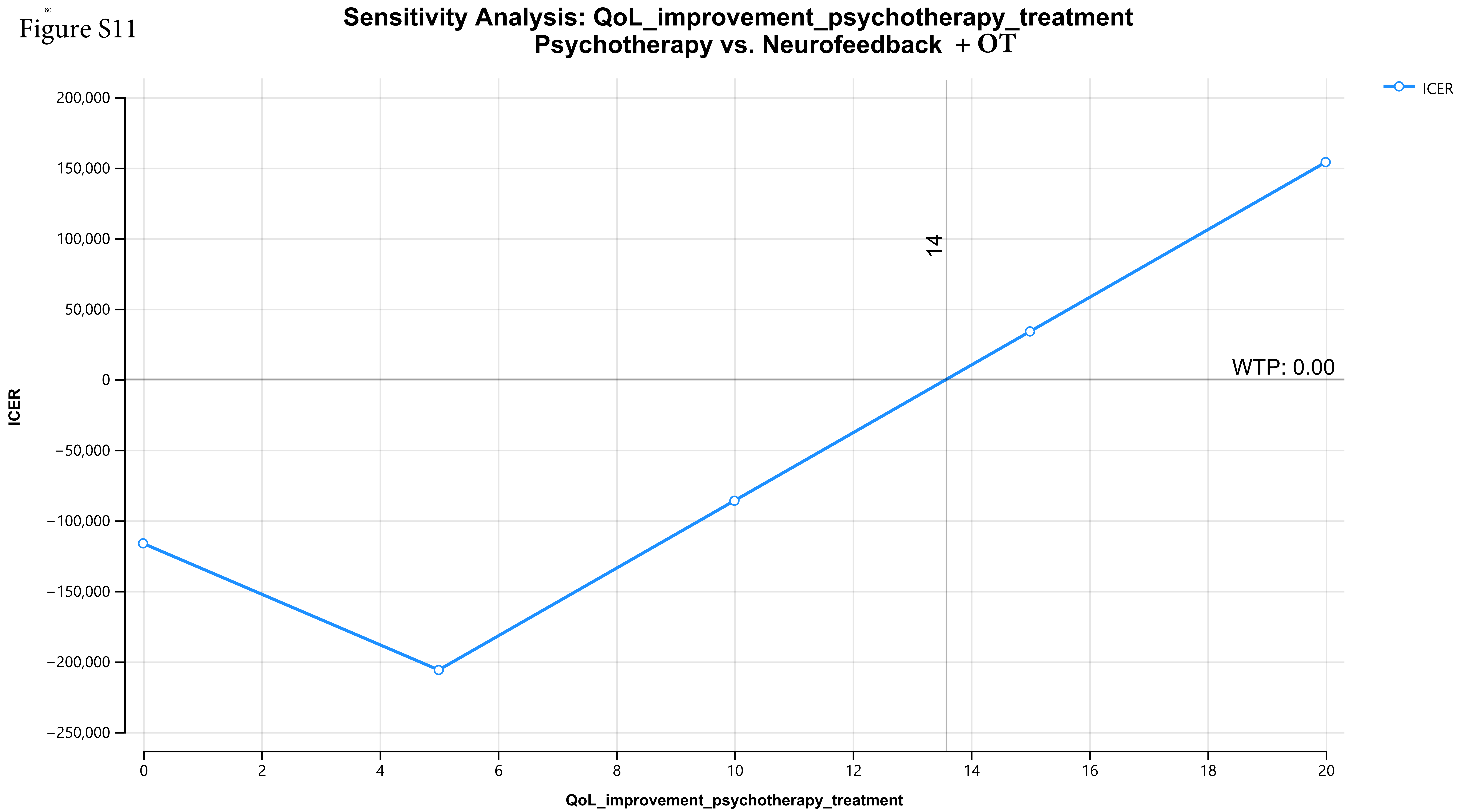

Sensitivity Analysis: QoL_improvement_psychotherapy_treatment
Psychotherapy vs. Neurofeedback + OT
ICER
200,000
150,000
100,000
50,000
0
−50,000
−100,000
−150,000
−200,000
−250,000
ICER
14
WTP: 0.00
0
2
4
6
8
10
12
14
16
18
20
QoL_improvement_psychotherapy_treatment

Table S1 – NF+OT vs. Psychotherapy and Pharmacotherapy

Therapy	Year 1 cost	Year 2 cost	Year 3 cost
Psychotherapy	$18,104	$30,524	$42,206
NF + other therapies	$15,536	$27,462	$38,066
Savings with NF +OT	$2,568	$3,062	$4,140

Therapy	Year 1 cost	Year 2 cost	Year 3 cost
Pharmacotherapy	$17,818	$32,197	$46,388
NF + other therapies	$15,536	$27,462	$39,171
Savings with NF + OT	$2,282	$4,735	$7,217

Table S2 – Prism +OT vs. Psychotherapy and Pharmacotherapy

Therapy	Year 1 cost	Year 2 cost	Year 3 cost
Psychotherapy	$18,104	$30,524	$42,206
Prism + other therapies	$11,338	$22,760	$33,939
Savings with Prism +OT	$6,766	$7,764	$8,267

Therapy	Year 1 cost	Year 2 cost	Year 3 cost
Pharmacotherapy	$17,818	$32,197	$46,388
Prism + other therapies	$11,338	$22,760	$33,939
Savings with Prism + OT	$6,480	$9,437	$12,449

Table S3 – Summary of outputs by stage and state NF+OT vs.

psychotherapy Psychotherapy

STAGE	STATE INDEX	STATE	PROBABILITY	STATE COST	TRANS COST	STAGE COST	TOTAL COST	STATE EFFECTIVENESS	STAGE EFFECTIVENESS	TOTAL EFFECTIVENESS
0	0	Less mild/Asymptomatic PTSD	0.00%	$0	$0	$5,134	$5,134	0.00	0.22	0.22
0	1	Mild PTSD treatment	30.20%	$1,285	-$87	$5,134	$5,134	0.07	0.22	0.22
0	2	Moderate PTSD treatment	33.20%	$1,715	-$95	$5,134	$5,134	0.08	0.22	0.22
0	3	Severe PTSD treatment	36.60%	$2,421	-$105	$5,134	$5,134	0.07	0.22	0.22
0	4	Dead	0.00%	$0	$0	$5,134	$5,134	0.00	0.22	0.22
0	5	Mild PTSD	0.00%	$0	$0	$5,134	$5,134	0.00	0.22	0.22
0	6	Moderate PTSD	0.00%	$0	$0	$5,134	$5,134	0.00	0.22	0.22
0	7	Severe PTSD	0.00%	$0	$0	$5,134	$5,134	0.00	0.22	0.22
0	8	Relapse mild PTSD	0.00%	$0	$0	$5,134	$5,134	0.00	0.22	0.22
0	9	Relapse moderate PTSD	0.00%	$0	$0	$5,134	$5,134	0.00	0.22	0.22
0	10	Relapse severe PTSD	0.00%	$0	$0	$5,134	$5,134	0.00	0.22	0.22
1	0	Less mild/Asymptomatic PTSD	23.13%	$583	$0	$3,493	$8,627	0.05	0.22	0.44
1	1	Mild PTSD treatment	25.41%	$641	-$72	$3,493	$8,627	0.06	0.22	0.44
1	2	Moderate PTSD treatment	28.00%	$1,436	-$80	$3,493	$8,627	0.07	0.22	0.44
1	3	Severe PTSD treatment	0.00%	$0	$0	$3,493	$8,627	0.00	0.22	0.44
1	4	Dead	0.18%	$0	$0	$3,493	$8,627	0.00	0.22	0.44
1	5	Mild PTSD	6.26%	$214	$0	$3,493	$8,627	0.01	0.22	0.44
1	6	Moderate PTSD	6.87%	$334	$0	$3,493	$8,627	0.01	0.22	0.44
1	7	Severe PTSD	7.57%	$437	$0	$3,493	$8,627	0.01	0.22	0.44
1	8	Relapse mild PTSD	0.78%	$0	$0	$3,493	$8,627	0.00	0.22	0.44
1	9	Relapse moderate PTSD	0.85%	$0	$0	$3,493	$8,627	0.00	0.22	0.44
1	10	Relapse severe PTSD	0.94%	$0	$0	$3,493	$8,627	0.00	0.22	0.44
2	0	Less mild/Asymptomatic PTSD	41.82%	$1,047	$0	$3,065	$11,692	0.10	0.22	0.66

STAGE	STATE INDEX	STATE	PROBABILITY	STATE COST	TRANS COST	STAGE COST	TOTAL COST	STATE EFFECTIVENESS	STAGE EFFECTIVENESS	TOTAL EFFECTIVENESS
2	1	Mild PTSD treatment	21.95%	$549	-$62	$3,065	$11,692	0.05	0.22	0.66
2	2	Moderate PTSD treatment	0.57%	$29	-$2	$3,065	$11,692	0.00	0.22	0.66
2	3	Severe PTSD treatment	0.63%	$30	-$2	$3,065	$11,692	0.00	0.22	0.66
2	4	Dead	0.34%	$0	$0	$3,065	$11,692	0.00	0.22	0.66
2	5	Mild PTSD	11.77%	$400	$0	$3,065	$11,692	0.03	0.22	0.66
2	6	Moderate PTSD	12.94%	$625	$0	$3,065	$11,692	0.02	0.22	0.66
2	7	Severe PTSD	7.87%	$450	$0	$3,065	$11,692	0.01	0.22	0.66
2	8	Relapse mild PTSD	1.40%	$0	$0	$3,065	$11,692	0.00	0.22	0.66
2	9	Relapse moderate PTSD	0.72%	$0	$0	$3,065	$11,692	0.00	0.22	0.66
2	10	Relapse severe PTSD	0.00%	$0	$0	$3,065	$11,692	0.00	0.22	0.66
3	0	Less mild/Asymptomatic PTSD	57.23%	$1,422	$0	$3,152	$14,844	0.13	0.21	0.87
3	1	Mild PTSD treatment	1.37%	$34	-$4	$3,152	$14,844	0.00	0.21	0.87
3	2	Moderate PTSD treatment	0.96%	$48	-$3	$3,152	$14,844	0.00	0.21	0.87
3	3	Severe PTSD treatment	0.00%	$0	$0	$3,152	$14,844	0.00	0.21	0.87
3	4	Dead	0.48%	$0	$0	$3,152	$14,844	0.00	0.21	0.87
3	5	Mild PTSD	16.76%	$565	$0	$3,152	$14,844	0.04	0.21	0.87
3	6	Moderate PTSD	13.27%	$636	$0	$3,152	$14,844	0.03	0.21	0.87
3	7	Severe PTSD	7.98%	$453	$0	$3,152	$14,844	0.01	0.21	0.87
3	8	Relapse mild PTSD	1.92%	$0	$0	$3,152	$14,844	0.00	0.21	0.87
3	9	Relapse moderate PTSD	0.01%	$0	$0	$3,152	$14,844	0.00	0.21	0.87
3	10	Relapse severe PTSD	0.02%	$0	$0	$3,152	$14,844	0.00	0.21	0.87
4	0	Less mild/Asymptomatic PTSD	56.36%	$1,390	$0	$3,115	$17,959	0.13	0.21	1.08
4	1	Mild PTSD treatment	2.01%	$50	-$6	$3,115	$17,959	0.00	0.21	1.08
4	2	Moderate PTSD treatment	0.01%	$0	$0	$3,115	$17,959	0.00	0.21	1.08
4	3	Severe PTSD treatment	0.01%	$1	$0	$3,115	$17,959	0.00	0.21	1.08
4	4	Dead	0.63%	$0	$0	$3,115	$17,959	0.00	0.21	1.08
4	5	Mild PTSD	17.65%	$591	$0	$3,115	$17,959	0.04	0.21	1.08

STAGE	STATE INDEX	STATE	PROBABILITY	STATE COST	TRANS COST	STAGE COST	TOTAL COST	STATE EFFECTIVENESS	STAGE EFFECTIVENESS	TOTAL EFFECTIVENESS
4	6	Moderate PTSD	13.45%	$640	$0	$3,115	$17,959	0.03	0.21	1.08
4	7	Severe PTSD	7.96%	$449	$0	$3,115	$17,959	0.01	0.21	1.08
4	8	Relapse mild PTSD	1.89%	$0	$0	$3,115	$17,959	0.00	0.21	1.08
4	9	Relapse moderate PTSD	0.02%	$0	$0	$3,115	$17,959	0.00	0.21	1.08
4	10	Relapse severe PTSD	0.00%	$0	$0	$3,115	$17,959	0.00	0.21	1.08
5	0	Less mild/Asymptomatic PTSD	56.01%	$1,371	$0	$3,099	$21,058	0.13	0.21	1.30
5	1	Mild PTSD treatment	1.27%	$31	-$4	$3,099	$21,058	0.00	0.21	1.30
5	2	Moderate PTSD treatment	0.02%	$1	$0	$3,099	$21,058	0.00	0.21	1.30
5	3	Severe PTSD treatment	0.00%	$0	$0	$3,099	$21,058	0.00	0.21	1.30
5	4	Dead	0.77%	$0	$0	$3,099	$21,058	0.00	0.21	1.30
5	5	Mild PTSD	18.67%	$621	$0	$3,099	$21,058	0.04	0.21	1.30
5	6	Moderate PTSD	13.43%	$634	$0	$3,099	$21,058	0.03	0.21	1.30
5	7	Severe PTSD	7.94%	$444	$0	$3,099	$21,058	0.01	0.21	1.30
5	8	Relapse mild PTSD	1.88%	$0	$0	$3,099	$21,058	0.00	0.21	1.30
5	9	Relapse moderate PTSD	0.00%	$0	$0	$3,099	$21,058	0.00	0.21	1.30
5	10	Relapse severe PTSD	0.00%	$0	$0	$3,099	$21,058	0.00	0.21	1.30
6	0	Less mild/Asymptomatic PTSD	55.11%	$1,339	$0	$3,079	$24,137	0.13	0.21	1.51
6	1	Mild PTSD treatment	1.27%	$31	-$3	$3,079	$24,137	0.00	0.21	1.51
6	2	Moderate PTSD treatment	0.00%	$0	$0	$3,079	$24,137	0.00	0.21	1.51
6	3	Severe PTSD treatment	0.00%	$0	$0	$3,079	$24,137	0.00	0.21	1.51
6	4	Dead	0.92%	$0	$0	$3,079	$24,137	0.00	0.21	1.51
6	5	Mild PTSD	19.52%	$644	$0	$3,079	$24,137	0.04	0.21	1.51
6	6	Moderate PTSD	13.40%	$628	$0	$3,079	$24,137	0.02	0.21	1.51
6	7	Severe PTSD	7.92%	$440	$0	$3,079	$24,137	0.01	0.21	1.51
6	8	Relapse mild PTSD	1.85%	$0	$0	$3,079	$24,137	0.00	0.21	1.51
6	9	Relapse moderate PTSD	0.00%	$0	$0	$3,079	$24,137	0.00	0.21	1.51
6	10	Relapse severe PTSD	0.00%	$0	$0	$3,079	$24,137	0.00	0.21	1.51

STAGE	STATE INDEX	STATE	PROBABILITY	STATE COST	TRANS COST	STAGE COST	TOTAL COST	STATE EFFECTIVENESS	STAGE EFFECTIVENESS	TOTAL EFFECTIVENESS
7	0	Less mild/Asymptomatic PTSD	54.24%	$1,308	$0	$3,060	$27,197	0.12	0.21	1.71
7	1	Mild PTSD treatment	1.23%	$30	-$3	$3,060	$27,197	0.00	0.21	1.71
7	2	Moderate PTSD treatment	0.00%	$0	$0	$3,060	$27,197	0.00	0.21	1.71
7	3	Severe PTSD treatment	0.00%	$0	$0	$3,060	$27,197	0.00	0.21	1.71
7	4	Dead	1.07%	$0	$0	$3,060	$27,197	0.00	0.21	1.71
7	5	Mild PTSD	20.37%	$667	$0	$3,060	$27,197	0.05	0.21	1.71
7	6	Moderate PTSD	13.38%	$622	$0	$3,060	$27,197	0.02	0.21	1.71
7	7	Severe PTSD	7.90%	$436	$0	$3,060	$27,197	0.01	0.21	1.71
7	8	Relapse mild PTSD	1.82%	$0	$0	$3,060	$27,197	0.00	0.21	1.71
7	9	Relapse moderate PTSD	0.00%	$0	$0	$3,060	$27,197	0.00	0.21	1.71
7	10	Relapse severe PTSD	0.00%	$0	$0	$3,060	$27,197	0.00	0.21	1.71
8	0	Less mild/Asymptomatic PTSD	53.36%	$1,278	$0	$3,040	$30,237	0.12	0.21	1.92
8	1	Mild PTSD treatment	1.21%	$29	-$3	$3,040	$30,237	0.00	0.21	1.92
8	2	Moderate PTSD treatment	0.00%	$0	$0	$3,040	$30,237	0.00	0.21	1.92
8	3	Severe PTSD treatment	0.00%	$0	$0	$3,040	$30,237	0.00	0.21	1.92
8	4	Dead	1.21%	$0	$0	$3,040	$30,237	0.00	0.21	1.92
8	5	Mild PTSD	21.19%	$689	$0	$3,040	$30,237	0.05	0.21	1.92
8	6	Moderate PTSD	13.35%	$616	$0	$3,040	$30,237	0.02	0.21	1.92
8	7	Severe PTSD	7.88%	$431	$0	$3,040	$30,237	0.01	0.21	1.92
8	8	Relapse mild PTSD	1.79%	$0	$0	$3,040	$30,237	0.00	0.21	1.92
8	9	Relapse moderate PTSD	0.00%	$0	$0	$3,040	$30,237	0.00	0.21	1.92
8	10	Relapse severe PTSD	0.00%	$0	$0	$3,040	$30,237	0.00	0.21	1.92
9	0	Less mild/Asymptomatic PTSD	52.50%	$1,248	$0	$3,021	$33,258	0.12	0.20	2.12
9	1	Mild PTSD treatment	1.19%	$28	-$3	$3,021	$33,258	0.00	0.20	2.12
9	2	Moderate PTSD treatment	0.00%	$0	$0	$3,021	$33,258	0.00	0.20	2.12
9	3	Severe PTSD treatment	0.00%	$0	$0	$3,021	$33,258	0.00	0.20	2.12

STAGE	STATE INDEX	STATE	PROBABILITY	STATE COST	TRANS COST	STAGE COST	TOTAL COST	STATE EFFECTIVENESS	STAGE EFFECTIVENESS	TOTAL EFFECTIVENESS
9	4	Dead	1.36%	$0	$0	$3,021	$33,258	0.00	0.20	2.12
9	5	Mild PTSD	22.00%	$710	$0	$3,021	$33,258	0.05	0.20	2.12
9	6	Moderate PTSD	13.32%	$611	$0	$3,021	$33,258	0.02	0.20	2.12
9	7	Severe PTSD	7.86%	$427	$0	$3,021	$33,258	0.01	0.20	2.12
9	8	Relapse mild PTSD	1.76%	$0	$0	$3,021	$33,258	0.00	0.20	2.12
9	9	Relapse moderate PTSD	0.00%	$0	$0	$3,021	$33,258	0.00	0.20	2.12
9	10	Relapse severe PTSD	0.00%	$0	$0	$3,021	$33,258	0.00	0.20	2.12
10	0	Less mild/Asymptomatic PTSD	51.66%	$1,219	$0	$3,001	$36,259	0.11	0.20	2.32
10	1	Mild PTSD treatment	1.17%	$28	-$3	$3,001	$36,259	0.00	0.20	2.32
10	2	Moderate PTSD treatment	0.00%	$0	$0	$3,001	$36,259	0.00	0.20	2.32
10	3	Severe PTSD treatment	0.00%	$0	$0	$3,001	$36,259	0.00	0.20	2.32
10	4	Dead	1.51%	$0	$0	$3,001	$36,259	0.00	0.20	2.32
10	5	Mild PTSD	22.80%	$730	$0	$3,001	$36,259	0.05	0.20	2.32
10	6	Moderate PTSD	13.29%	$605	$0	$3,001	$36,259	0.02	0.20	2.32
10	7	Severe PTSD	7.84%	$423	$0	$3,001	$36,259	0.01	0.20	2.32
10	8	Relapse mild PTSD	1.73%	$0	$0	$3,001	$36,259	0.00	0.20	2.32
10	9	Relapse moderate PTSD	0.00%	$0	$0	$3,001	$36,259	0.00	0.20	2.32
10	10	Relapse severe PTSD	0.00%	$0	$0	$3,001	$36,259	0.00	0.20	2.32
11	0	Less mild/Asymptomatic PTSD	50.83%	$1,190	$0	$2,982	$39,241	0.11	0.20	2.53
11	1	Mild PTSD treatment	1.16%	$27	-$3	$2,982	$39,241	0.00	0.20	2.53
11	2	Moderate PTSD treatment	0.00%	$0	$0	$2,982	$39,241	0.00	0.20	2.53
11	3	Severe PTSD treatment	0.00%	$0	$0	$2,982	$39,241	0.00	0.20	2.53
11	4	Dead	1.66%	$0	$0	$2,982	$39,241	0.00	0.20	2.53
11	5	Mild PTSD	23.58%	$750	$0	$2,982	$39,241	0.05	0.20	2.53
11	6	Moderate PTSD	13.26%	$599	$0	$2,982	$39,241	0.02	0.20	2.53
11	7	Severe PTSD	7.82%	$419	$0	$2,982	$39,241	0.01	0.20	2.53
11	8	Relapse mild PTSD	1.71%	$0	$0	$2,982	$39,241	0.00	0.20	2.53

STAGE	STATE INDEX	STATE	PROBABILITY	STATE COST	TRANS COST	STAGE COST	TOTAL COST	STATE EFFECTIVENESS	STAGE EFFECTIVENESS	TOTAL EFFECTIVENESS
11	9	Relapse moderate PTSD	0.00%	$0	$0	$2,982	$39,241	0.00	0.20	2.53
11	10	Relapse severe PTSD	0.00%	$0	$0	$2,982	$39,241	0.00	0.20	2.53
12	0	Less mild/Asymptomatic PTSD	50.01%	$1,162	$0	$2,965	$42,206	0.11	0.20	2.72
12	1	Mild PTSD treatment	1.14%	$26	$0	$2,965	$42,206	0.00	0.20	2.72
12	2	Moderate PTSD treatment	0.00%	$0	$0	$2,965	$42,206	0.00	0.20	2.72
12	3	Severe PTSD treatment	0.00%	$0	$0	$2,965	$42,206	0.00	0.20	2.72
12	4	Dead	1.80%	$0	$0	$2,965	$42,206	0.00	0.20	2.72
12	5	Mild PTSD	24.34%	$769	$0	$2,965	$42,206	0.05	0.20	2.72
12	6	Moderate PTSD	13.23%	$593	$0	$2,965	$42,206	0.02	0.20	2.72
12	7	Severe PTSD	7.80%	$414	$0	$2,965	$42,206	0.01	0.20	2.72
12	8	Relapse mild PTSD	1.68%	$0	$0	$2,965	$42,206	0.00	0.20	2.72
12	9	Relapse moderate PTSD	0.00%	$0	$0	$2,965	$42,206	0.00	0.20	2.72
12	10	Relapse severe PTSD	0.00%	$0	$0	$2,965	$42,206	0.00	0.20	2.72

eTable 3 - NF +OT

STAGE	STATE INDEX	STATE	PROBABILITY	STATE COST	TRANS COST	STAGE COST	TOTAL COST	STATE EFFECTIVENESS	STAGE EFFECTIVENESS	TOTAL EFFECTIVENESS
0	0	Less mild/Asympotmatic PTSD	0.00%	$0	$0	$3,564	$3,564	0.00	0.22	0.22
0	1	Mild PTSD treatment	30.20%	$835	-$111	$3,564	$3,564	0.07	0.22	0.22
0	2	Moderate PTSD treatment	33.20%	$1,221	-$122	$3,564	$3,564	0.08	0.22	0.22
0	3	Severe PTSD treatment	36.60%	$1,876	-$135	$3,564	$3,564	0.07	0.22	0.22
0	4	Dead	0.00%	$0	$0	$3,564	$3,564	0.00	0.22	0.22
0	5	Mild PTSD	0.00%	$0	$0	$3,564	$3,564	0.00	0.22	0.22
0	6	Moderate PTSD	0.00%	$0	$0	$3,564	$3,564	0.00	0.22	0.22
0	7	Severe PTSD	0.00%	$0	$0	$3,564	$3,564	0.00	0.22	0.22
0	8	Relapse mild PTSD	0.00%	$0	$0	$3,564	$3,564	0.00	0.22	0.22
0	9	Relapse moderate PTSD	0.00%	$0	$0	$3,564	$3,564	0.00	0.22	0.22

STAGE	STATE INDEX	STATE	PROBABILITY	STATE COST	TRANS COST	STAGE COST	TOTAL COST	STATE EFFECTIVENESS	STAGE EFFECTIVENESS	TOTAL EFFECTIVENESS
0	10	Relapse severe PTSD	0.00%	$0	$0	$3,564	$3,564	0.00	0.22	0.22
1	0	Less mild/Asympotmatic PTSD	25.34%	$661	$0	$2,855	$6,419	0.06	0.22	0.44
1	1	Mild PTSD treatment	27.83%	$715	-$102	$2,855	$6,419	0.07	0.22	0.44
1	2	Moderate PTSD treatment	30.68%	$1,065	-$112	$2,855	$6,419	0.07	0.22	0.44
1	3	Severe PTSD treatment	0.00%	$0	$0	$2,855	$6,419	0.00	0.22	0.44
1	4	Dead	0.17%	$0	$0	$2,855	$6,419	0.00	0.22	0.44
1	5	Mild PTSD	3.98%	$136	$0	$2,855	$6,419	0.01	0.22	0.44
1	6	Moderate PTSD	4.37%	$213	$0	$2,855	$6,419	0.01	0.22	0.44
1	7	Severe PTSD	4.82%	$278	$0	$2,855	$6,419	0.01	0.22	0.44
1	8	Relapse mild PTSD	0.85%	$0	$0	$2,855	$6,419	0.00	0.22	0.44
1	9	Relapse moderate PTSD	0.93%	$0	$0	$2,855	$6,419	0.00	0.22	0.44
1	10	Relapse severe PTSD	1.03%	$0	$0	$2,855	$6,419	0.00	0.22	0.44
2	0	Less mild/Asympotmatic PTSD	47.83%	$1,239	$0	$2,849	$9,268	0.11	0.22	0.66
2	1	Mild PTSD treatment	26.28%	$670	-$95	$2,849	$9,268	0.06	0.22	0.66
2	2	Moderate PTSD treatment	0.62%	$21	-$2	$2,849	$9,268	0.00	0.22	0.66
2	3	Severe PTSD treatment	0.69%	$33	-$2	$2,849	$9,268	0.00	0.22	0.66
2	4	Dead	0.32%	$0	$0	$2,849	$9,268	0.00	0.22	0.66
2	5	Mild PTSD	7.92%	$269	$0	$2,849	$9,268	0.02	0.22	0.66
2	6	Moderate PTSD	8.72%	$421	$0	$2,849	$9,268	0.02	0.22	0.66
2	7	Severe PTSD	5.15%	$295	$0	$2,849	$9,268	0.01	0.22	0.66
2	8	Relapse mild PTSD	1.61%	$0	$0	$2,849	$9,268	0.00	0.22	0.66
2	9	Relapse moderate PTSD	0.86%	$0	$0	$2,849	$9,268	0.00	0.22	0.66
2	10	Relapse severe PTSD	0.00%	$0	$0	$2,849	$9,268	0.00	0.22	0.66
3	0	Less mild/Asympotmatic PTSD	68.28%	$1,756	$0	$2,959	$12,228	0.16	0.22	0.88
3	1	Mild PTSD treatment	1.59%	$40	-$6	$2,959	$12,228	0.00	0.22	0.88
3	2	Moderate PTSD treatment	1.15%	$39	-$4	$2,959	$12,228	0.00	0.22	0.88
3	3	Severe PTSD treatment	0.00%	$0	$0	$2,959	$12,228	0.00	0.22	0.88
3	4	Dead	0.45%	$0	$0	$2,959	$12,228	0.00	0.22	0.88
3	5	Mild PTSD	11.91%	$402	$0	$2,959	$12,228	0.03	0.22	0.88

STAGE	STATE INDEX	STATE	PROBABILITY	STATE COST	TRANS COST	STAGE COST	TOTAL COST	STATE EFFECTIVENESS	STAGE EFFECTIVENESS	TOTAL EFFECTIVENESS
3	6	Moderate PTSD	9.07%	$435	$0	$2,959	$12,228	0.02	0.22	0.88
3	7	Severe PTSD	5.23%	$297	$0	$2,959	$12,228	0.01	0.22	0.88
3	8	Relapse mild PTSD	2.29%	$0	$0	$2,959	$12,228	0.00	0.22	0.88
3	9	Relapse moderate PTSD	0.02%	$0	$0	$2,959	$12,228	0.00	0.22	0.88
3	10	Relapse severe PTSD	0.02%	$0	$0	$2,959	$12,228	0.00	0.22	0.88
4	0	Less mild/Asympotmatic PTSD	67.33%	$1,719	$0	$2,936	$15,164	0.16	0.22	1.10
4	1	Mild PTSD treatment	2.49%	$63	-$9	$2,936	$15,164	0.01	0.22	1.10
4	2	Moderate PTSD treatment	0.01%	$0	$0	$2,936	$15,164	0.00	0.22	1.10
4	3	Severe PTSD treatment	0.01%	$1	$0	$2,936	$15,164	0.00	0.22	1.10
4	4	Dead	0.58%	$0	$0	$2,936	$15,164	0.00	0.22	1.10
4	5	Mild PTSD	12.86%	$431	$0	$2,936	$15,164	0.03	0.22	1.10
4	6	Moderate PTSD	9.21%	$438	$0	$2,936	$15,164	0.02	0.22	1.10
4	7	Severe PTSD	5.22%	$294	$0	$2,936	$15,164	0.01	0.22	1.10
4	8	Relapse mild PTSD	2.26%	$0	$0	$2,936	$15,164	0.00	0.22	1.10
4	9	Relapse moderate PTSD	0.03%	$0	$0	$2,936	$15,164	0.00	0.22	1.10
4	10	Relapse severe PTSD	0.00%	$0	$0	$2,936	$15,164	0.00	0.22	1.10
5	0	Less mild/Asympotmatic PTSD	67.16%	$1,702	$0	$2,924	$18,088	0.15	0.21	1.31
5	1	Mild PTSD treatment	1.52%	$38	-$5	$2,924	$18,088	0.00	0.21	1.31
5	2	Moderate PTSD treatment	0.03%	$1	$0	$2,924	$18,088	0.00	0.21	1.31
5	3	Severe PTSD treatment	0.00%	$0	$0	$2,924	$18,088	0.00	0.21	1.31
5	4	Dead	0.71%	$0	$0	$2,924	$18,088	0.00	0.21	1.31
5	5	Mild PTSD	13.92%	$463	$0	$2,924	$18,088	0.03	0.21	1.31
5	6	Moderate PTSD	9.20%	$434	$0	$2,924	$18,088	0.02	0.21	1.31
5	7	Severe PTSD	5.21%	$291	$0	$2,924	$18,088	0.01	0.21	1.31
5	8	Relapse mild PTSD	2.25%	$0	$0	$2,924	$18,088	0.00	0.21	1.31
5	9	Relapse moderate PTSD	0.00%	$0	$0	$2,924	$18,088	0.00	0.21	1.31
5	10	Relapse severe PTSD	0.00%	$0	$0	$2,924	$18,088	0.00	0.21	1.31
6	0	Less mild/Asympotmatic PTSD	66.18%	$1,665	$0	$2,906	$20,994	0.15	0.21	1.52
6	1	Mild PTSD treatment	1.53%	$38	-$5	$2,906	$20,994	0.00	0.21	1.52

STAGE	STATE INDEX	STATE	PROBABILITY	STATE COST	TRANS COST	STAGE COST	TOTAL COST	STATE EFFECTIVENESS	STAGE EFFECTIVENESS	TOTAL EFFECTIVENESS
6	2	Moderate PTSD treatment	0.00%	$0	$0	$2,906	$20,994	0.00	0.21	1.52
6	3	Severe PTSD treatment	0.00%	$0	$0	$2,906	$20,994	0.00	0.21	1.52
6	4	Dead	0.85%	$0	$0	$2,906	$20,994	0.00	0.21	1.52
6	5	Mild PTSD	14.84%	$490	$0	$2,906	$20,994	0.03	0.21	1.52
6	6	Moderate PTSD	9.18%	$430	$0	$2,906	$20,994	0.02	0.21	1.52
6	7	Severe PTSD	5.19%	$288	$0	$2,906	$20,994	0.01	0.21	1.52
6	8	Relapse mild PTSD	2.22%	$0	$0	$2,906	$20,994	0.00	0.21	1.52
6	9	Relapse moderate PTSD	0.00%	$0	$0	$2,906	$20,994	0.00	0.21	1.52
6	10	Relapse severe PTSD	0.00%	$0	$0	$2,906	$20,994	0.00	0.21	1.52
7	0	Less mild/Asympotmatic PTSD	65.25%	$1,629	$0	$2,889	$23,883	0.15	0.21	1.73
7	1	Mild PTSD treatment	1.48%	$36	-$5	$2,889	$23,883	0.00	0.21	1.73
7	2	Moderate PTSD treatment	0.00%	$0	$0	$2,889	$23,883	0.00	0.21	1.73
7	3	Severe PTSD treatment	0.00%	$0	$0	$2,889	$23,883	0.00	0.21	1.73
7	4	Dead	0.98%	$0	$0	$2,889	$23,883	0.00	0.21	1.73
7	5	Mild PTSD	15.76%	$516	$0	$2,889	$23,883	0.04	0.21	1.73
7	6	Moderate PTSD	9.16%	$426	$0	$2,889	$23,883	0.02	0.21	1.73
7	7	Severe PTSD	5.18%	$286	$0	$2,889	$23,883	0.01	0.21	1.73
7	8	Relapse mild PTSD	2.19%	$0	$0	$2,889	$23,883	0.00	0.21	1.73
7	9	Relapse moderate PTSD	0.00%	$0	$0	$2,889	$23,883	0.00	0.21	1.73
7	10	Relapse severe PTSD	0.00%	$0	$0	$2,889	$23,883	0.00	0.21	1.73
8	0	Less mild/Asympotmatic PTSD	64.30%	$1,594	$0	$2,871	$26,754	0.14	0.21	1.94
8	1	Mild PTSD treatment	1.46%	$36	-$5	$2,871	$26,754	0.00	0.21	1.94
8	2	Moderate PTSD treatment	0.00%	$0	$0	$2,871	$26,754	0.00	0.21	1.94
8	3	Severe PTSD treatment	0.00%	$0	$0	$2,871	$26,754	0.00	0.21	1.94
8	4	Dead	1.11%	$0	$0	$2,871	$26,754	0.00	0.21	1.94
8	5	Mild PTSD	16.65%	$542	$0	$2,871	$26,754	0.04	0.21	1.94
8	6	Moderate PTSD	9.14%	$422	$0	$2,871	$26,754	0.02	0.21	1.94
8	7	Severe PTSD	5.17%	$283	$0	$2,871	$26,754	0.01	0.21	1.94
8	8	Relapse mild PTSD	2.16%	$0	$0	$2,871	$26,754	0.00	0.21	1.94

STAGE	STATE INDEX	STATE	PROBABILITY	STATE COST	TRANS COST	STAGE COST	TOTAL COST	STATE EFFECTIVENESS	STAGE EFFECTIVENESS	TOTAL EFFECTIVENESS
8	9	Relapse moderate PTSD	0.00%	$0	$0	$2,871	$26,754	0.00	0.21	1.94
8	10	Relapse severe PTSD	0.00%	$0	$0	$2,871	$26,754	0.00	0.21	1.94
9	0	Less mild/Asympotmatic PTSD	63.37%	$1,559	$0	$2,853	$29,608	0.14	0.21	2.15
9	1	Mild PTSD treatment	1.44%	$35	-$5	$2,853	$29,608	0.00	0.21	2.15
9	2	Moderate PTSD treatment	0.00%	$0	$0	$2,853	$29,608	0.00	0.21	2.15
9	3	Severe PTSD treatment	0.00%	$0	$0	$2,853	$29,608	0.00	0.21	2.15
9	4	Dead	1.25%	$0	$0	$2,853	$29,608	0.00	0.21	2.15
9	5	Mild PTSD	17.54%	$566	$0	$2,853	$29,608	0.04	0.21	2.15
9	6	Moderate PTSD	9.12%	$418	$0	$2,853	$29,608	0.02	0.21	2.15
9	7	Severe PTSD	5.15%	$280	$0	$2,853	$29,608	0.01	0.21	2.15
9	8	Relapse mild PTSD	2.13%	$0	$0	$2,853	$29,608	0.00	0.21	2.15
9	9	Relapse moderate PTSD	0.00%	$0	$0	$2,853	$29,608	0.00	0.21	2.15
9	10	Relapse severe PTSD	0.00%	$0	$0	$2,853	$29,608	0.00	0.21	2.15
10	0	Less mild/Asympotmatic PTSD	62.46%	$1,525	$0	$2,836	$32,443	0.14	0.21	2.36
10	1	Mild PTSD treatment	1.42%	$34	-$5	$2,836	$32,443	0.00	0.21	2.36
10	2	Moderate PTSD treatment	0.00%	$0	$0	$2,836	$32,443	0.00	0.21	2.36
10	3	Severe PTSD treatment	0.00%	$0	$0	$2,836	$32,443	0.00	0.21	2.36
10	4	Dead	1.38%	$0	$0	$2,836	$32,443	0.00	0.21	2.36
10	5	Mild PTSD	18.40%	$590	$0	$2,836	$32,443	0.04	0.21	2.36
10	6	Moderate PTSD	9.10%	$414	$0	$2,836	$32,443	0.02	0.21	2.36
10	7	Severe PTSD	5.14%	$277	$0	$2,836	$32,443	0.01	0.21	2.36
10	8	Relapse mild PTSD	2.10%	$0	$0	$2,836	$32,443	0.00	0.21	2.36
10	9	Relapse moderate PTSD	0.00%	$0	$0	$2,836	$32,443	0.00	0.21	2.36
10	10	Relapse severe PTSD	0.00%	$0	$0	$2,836	$32,443	0.00	0.21	2.36
11	0	Less mild/Asympotmatic PTSD	61.55%	$1,492	$0	$2,818	$35,261	0.13	0.20	2.56
11	1	Mild PTSD treatment	1.40%	$33	-$5	$2,818	$35,261	0.00	0.20	2.56
11	2	Moderate PTSD treatment	0.00%	$0	$0	$2,818	$35,261	0.00	0.20	2.56
11	3	Severe PTSD treatment	0.00%	$0	$0	$2,818	$35,261	0.00	0.20	2.56
11	4	Dead	1.52%	$0	$0	$2,818	$35,261	0.00	0.20	2.56

STAGE	STATE INDEX	STATE	PROBABILITY	STATE COST	TRANS COST	STAGE COST	TOTAL COST	STATE EFFECTIVENESS	STAGE EFFECTIVENESS	TOTAL EFFECTIVENESS
11	5	Mild PTSD	19.25%	$612	$0	$2,818	$35,261	0.04	0.20	2.56
11	6	Moderate PTSD	9.08%	$410	$0	$2,818	$35,261	0.02	0.20	2.56
11	7	Severe PTSD	5.12%	$274	$0	$2,818	$35,261	0.01	0.20	2.56
11	8	Relapse mild PTSD	2.07%	$0	$0	$2,818	$35,261	0.00	0.20	2.56
11	9	Relapse moderate PTSD	0.00%	$0	$0	$2,818	$35,261	0.00	0.20	2.56
11	10	Relapse severe PTSD	0.00%	$0	$0	$2,818	$35,261	0.00	0.20	2.56
12	0	Less mild/Asympotmatic PTSD	60.66%	$1,460	$0	$2,805	$38,066	0.13	0.20	2.76
12	1	Mild PTSD treatment	1.38%	$33	$0	$2,805	$38,066	0.00	0.20	2.76
12	2	Moderate PTSD treatment	0.00%	$0	$0	$2,805	$38,066	0.00	0.20	2.76
12	3	Severe PTSD treatment	0.00%	$0	$0	$2,805	$38,066	0.00	0.20	2.76
12	4	Dead	1.65%	$0	$0	$2,805	$38,066	0.00	0.20	2.76
12	5	Mild PTSD	20.09%	$634	$0	$2,805	$38,066	0.04	0.20	2.76
12	6	Moderate PTSD	9.06%	$406	$0	$2,805	$38,066	0.02	0.20	2.76
12	7	Severe PTSD	5.11%	$272	$0	$2,805	$38,066	0.01	0.20	2.76
12	8	Relapse mild PTSD	2.04%	$0	$0	$2,805	$38,066	0.00	0.20	2.76
12	9	Relapse moderate PTSD	0.00%	$0	$0	$2,805	$38,066	0.00	0.20	2.76
12	10	Relapse severe PTSD	0.00%	$0	$0	$2,805	$38,066	0.00	0.20	2.76

Table S4 – Summary outputs by stage and state NF+OT vs.

pharmacotherapy Pharmacotherapy

STAGE	STATE INDEX	STATE	PROBABILITY	STATE COST	TRANS COST	STAGE COST	TOTAL COST	STATE EFFECTIVENESS	STAGE EFFECTIVENESS	TOTAL EFFECTIVENESS
0	0	Less mild/Asymptomatic PTSD	0.00%	$0	$0	$3,838	$3,838	0.00	0.22	0.22
0	1	Mild PTSD treatment	30.20%	$876	-$69	$3,838	$3,838	0.07	0.22	0.22
0	2	Moderate PTSD treatment	33.20%	$1,265	-$76	$3,838	$3,838	0.07	0.22	0.22
0	3	Severe PTSD treatment	36.60%	$1,926	-$84	$3,838	$3,838	0.07	0.22	0.22
0	4	Dead	0.00%	$0	$0	$3,838	$3,838	0.00	0.22	0.22
0	5	Mild PTSD	0.00%	$0	$0	$3,838	$3,838	0.00	0.22	0.22
0	6	Moderate PTSD	0.00%	$0	$0	$3,838	$3,838	0.00	0.22	0.22
0	7	Severe PTSD	0.00%	$0	$0	$3,838	$3,838	0.00	0.22	0.22
0	8	Relapse mild PTSD	0.00%	$0	$0	$3,838	$3,838	0.00	0.22	0.22
0	9	Relapse moderate PTSD	0.00%	$0	$0	$3,838	$3,838	0.00	0.22	0.22
0	10	Relapse severe PTSD	0.00%	$0	$0	$3,838	$3,838	0.00	0.22	0.22
1	0	Less mild/Asymptomatic PTSD	16.70%	$481	$0	$3,253	$7,090	0.04	0.19	0.41
1	1	Mild PTSD treatment	18.34%	$528	-$42	$3,253	$7,090	0.04	0.19	0.41
1	2	Moderate PTSD treatment	20.21%	$765	-$46	$3,253	$7,090	0.04	0.19	0.41
1	3	Severe PTSD treatment	0.00%	$0	$0	$3,253	$7,090	0.00	0.19	0.41
1	4	Dead	0.20%	$0	$0	$3,253	$7,090	0.00	0.19	0.41
1	5	Mild PTSD	9.95%	$341	$0	$3,253	$7,090	0.02	0.19	0.41
1	6	Moderate PTSD	10.93%	$532	$0	$3,253	$7,090	0.02	0.19	0.41
1	7	Severe PTSD	12.05%	$694	$0	$3,253	$7,090	0.02	0.19	0.41
1	8	Relapse mild PTSD	3.51%	$0	$0	$3,253	$7,090	0.00	0.19	0.41
1	9	Relapse moderate PTSD	3.86%	$0	$0	$3,253	$7,090	0.00	0.19	0.41
1	10	Relapse severe PTSD	4.26%	$0	$0	$3,253	$7,090	0.00	0.19	0.41
2	0	Less mild/Asymptomatic PTSD	23.92%	$684	$0	$3,532	$10,623	0.06	0.19	0.60
2	1	Mild PTSD treatment	13.50%	$386	-$31	$3,532	$10,623	0.03	0.19	0.60
2	2	Moderate PTSD treatment	2.57%	$97	-$6	$3,532	$10,623	0.01	0.19	0.60

STAGE	STATE INDEX	STATE	PROBABILITY	STATE COST	TRANS COST	STAGE COST	TOTAL COST	STATE EFFECTIVENESS	STAGE EFFECTIVENESS	TOTAL EFFECTIVENESS
2	3	Severe PTSD treatment	2.84%	$147	-$6	$3,532	$10,623	0.01	0.19	0.60
2	4	Dead	0.37%	$0	$0	$3,532	$10,623	0.00	0.19	0.60
2	5	Mild PTSD	17.14%	$583	$0	$3,532	$10,623	0.04	0.19	0.60
2	6	Moderate PTSD	18.85%	$910	$0	$3,532	$10,623	0.04	0.19	0.60
2	7	Severe PTSD	13.43%	$769	$0	$3,532	$10,623	0.02	0.19	0.60
2	8	Relapse mild PTSD	5.03%	$0	$0	$3,532	$10,623	0.00	0.19	0.60
2	9	Relapse moderate PTSD	2.35%	$0	$0	$3,532	$10,623	0.00	0.19	0.60
2	10	Relapse severe PTSD	0.00%	$0	$0	$3,532	$10,623	0.00	0.19	0.60
3	0	Less mild/Asymptomatic PTSD	27.20%	$772	$0	$3,584	$14,207	0.06	0.19	0.79
3	1	Mild PTSD treatment	4.77%	$136	-$11	$3,584	$14,207	0.01	0.19	0.79
3	2	Moderate PTSD treatment	3.13%	$117	-$7	$3,584	$14,207	0.01	0.19	0.79
3	3	Severe PTSD treatment	0.00%	$0	$0	$3,584	$14,207	0.00	0.19	0.79
3	4	Dead	0.54%	$0	$0	$3,584	$14,207	0.00	0.19	0.79
3	5	Mild PTSD	23.23%	$784	$0	$3,584	$14,207	0.05	0.19	0.79
3	6	Moderate PTSD	20.44%	$980	$0	$3,584	$14,207	0.04	0.19	0.79
3	7	Severe PTSD	14.33%	$814	$0	$3,584	$14,207	0.02	0.19	0.79
3	8	Relapse mild PTSD	5.72%	$0	$0	$3,584	$14,207	0.00	0.19	0.79
3	9	Relapse moderate PTSD	0.30%	$0	$0	$3,584	$14,207	0.00	0.19	0.79
3	10	Relapse severe PTSD	0.33%	$0	$0	$3,584	$14,207	0.00	0.19	0.79
4	0	Less mild/Asymptomatic PTSD	25.08%	$707	$0	$3,598	$17,804	0.06	0.19	0.99
4	1	Mild PTSD treatment	5.55%	$156	-$12	$3,598	$17,804	0.01	0.19	0.99
4	2	Moderate PTSD treatment	0.20%	$7	$0	$3,598	$17,804	0.00	0.19	0.99
4	3	Severe PTSD treatment	0.22%	$11	$0	$3,598	$17,804	0.00	0.19	0.99
4	4	Dead	0.71%	$0	$0	$3,598	$17,804	0.00	0.19	0.99
4	5	Mild PTSD	26.67%	$893	$0	$3,598	$17,804	0.06	0.19	0.99
4	6	Moderate PTSD	21.53%	$1,024	$0	$3,598	$17,804	0.04	0.19	0.99
4	7	Severe PTSD	14.40%	$812	$0	$3,598	$17,804	0.02	0.19	0.99
4	8	Relapse mild PTSD	5.28%	$0	$0	$3,598	$17,804	0.00	0.19	0.99
4	9	Relapse moderate PTSD	0.36%	$0	$0	$3,598	$17,804	0.00	0.19	0.99

STAGE	STATE INDEX	STATE	PROBABILITY	STATE COST	TRANS COST	STAGE COST	TOTAL COST	STATE EFFECTIVENESS	STAGE EFFECTIVENESS	TOTAL EFFECTIVENESS
4	10	Relapse severe PTSD	0.00%	$0	$0	$3,598	$17,804	0.00	0.19	0.99
5	0	Less mild/Asymptomatic PTSD	23.76%	$664	$0	$3,606	$21,410	0.05	0.19	1.18
5	1	Mild PTSD treatment	3.63%	$101	-$8	$3,606	$21,410	0.01	0.19	1.18
5	2	Moderate PTSD treatment	0.36%	$13	-$1	$3,606	$21,410	0.00	0.19	1.18
5	3	Severe PTSD treatment	0.00%	$0	$0	$3,606	$21,410	0.00	0.19	1.18
5	4	Dead	0.88%	$0	$0	$3,606	$21,410	0.00	0.19	1.18
5	5	Mild PTSD	30.21%	$1,004	$0	$3,606	$21,410	0.06	0.19	1.18
5	6	Moderate PTSD	21.67%	$1,023	$0	$3,606	$21,410	0.04	0.19	1.18
5	7	Severe PTSD	14.44%	$808	$0	$3,606	$21,410	0.02	0.19	1.18
5	8	Relapse mild PTSD	5.00%	$0	$0	$3,606	$21,410	0.00	0.19	1.18
5	9	Relapse moderate PTSD	0.02%	$0	$0	$3,606	$21,410	0.00	0.19	1.18
5	10	Relapse severe PTSD	0.03%	$0	$0	$3,606	$21,410	0.00	0.19	1.18
6	0	Less mild/Asymptomatic PTSD	21.61%	$600	$0	$3,601	$25,012	0.05	0.19	1.37
6	1	Mild PTSD treatment	3.53%	$98	-$8	$3,601	$25,012	0.01	0.19	1.37
6	2	Moderate PTSD treatment	0.02%	$1	$0	$3,601	$25,012	0.00	0.19	1.37
6	3	Severe PTSD treatment	0.02%	$1	$0	$3,601	$25,012	0.00	0.19	1.37
6	4	Dead	1.05%	$0	$0	$3,601	$25,012	0.00	0.19	1.37
6	5	Mild PTSD	33.01%	$1,090	$0	$3,601	$25,012	0.07	0.19	1.37
6	6	Moderate PTSD	21.75%	$1,020	$0	$3,601	$25,012	0.04	0.19	1.37
6	7	Severe PTSD	14.41%	$800	$0	$3,601	$25,012	0.02	0.19	1.37
6	8	Relapse mild PTSD	4.55%	$0	$0	$3,601	$25,012	0.00	0.19	1.37
6	9	Relapse moderate PTSD	0.04%	$0	$0	$3,601	$25,012	0.00	0.19	1.37
6	10	Relapse severe PTSD	0.00%	$0	$0	$3,601	$25,012	0.00	0.19	1.37
7	0	Less mild/Asymptomatic PTSD	19.79%	$545	$0	$3,595	$28,606	0.04	0.19	1.56
7	1	Mild PTSD treatment	3.04%	$84	-$7	$3,595	$28,606	0.01	0.19	1.56
7	2	Moderate PTSD treatment	0.04%	$1	$0	$3,595	$28,606	0.00	0.19	1.56
7	3	Severe PTSD treatment	0.00%	$0	$0	$3,595	$28,606	0.00	0.19	1.56
7	4	Dead	1.23%	$0	$0	$3,595	$28,606	0.00	0.19	1.56
7	5	Mild PTSD	35.63%	$1,167	$0	$3,595	$28,606	0.08	0.19	1.56

STAGE	STATE INDEX	STATE	PROBABILITY	STATE COST	TRANS COST	STAGE COST	TOTAL COST	STATE EFFECTIVENESS	STAGE EFFECTIVENESS	TOTAL EFFECTIVENESS
7	6	Moderate PTSD	21.73%	$1,011	$0	$3,595	$28,606	0.04	0.19	1.56
7	7	Severe PTSD	14.38%	$793	$0	$3,595	$28,606	0.02	0.19	1.56
7	8	Relapse mild PTSD	4.16%	$0	$0	$3,595	$28,606	0.00	0.19	1.56
7	9	Relapse moderate PTSD	0.00%	$0	$0	$3,595	$28,606	0.00	0.19	1.56
7	10	Relapse severe PTSD	0.00%	$0	$0	$3,595	$28,606	0.00	0.19	1.56
8	0	Less mild/Asymptomatic PTSD	18.01%	$493	$0	$3,584	$32,190	0.04	0.19	1.75
8	1	Mild PTSD treatment	2.80%	$76	-$6	$3,584	$32,190	0.01	0.19	1.75
8	2	Moderate PTSD treatment	0.00%	$0	$0	$3,584	$32,190	0.00	0.19	1.75
8	3	Severe PTSD treatment	0.00%	$0	$0	$3,584	$32,190	0.00	0.19	1.75
8	4	Dead	1.41%	$0	$0	$3,584	$32,190	0.00	0.19	1.75
8	5	Mild PTSD	37.96%	$1,234	$0	$3,584	$32,190	0.08	0.19	1.75
8	6	Moderate PTSD	21.69%	$1,002	$0	$3,584	$32,190	0.04	0.19	1.75
8	7	Severe PTSD	14.34%	$785	$0	$3,584	$32,190	0.02	0.19	1.75
8	8	Relapse mild PTSD	3.79%	$0	$0	$3,584	$32,190	0.00	0.19	1.75
8	9	Relapse moderate PTSD	0.00%	$0	$0	$3,584	$32,190	0.00	0.19	1.75
8	10	Relapse severe PTSD	0.00%	$0	$0	$3,584	$32,190	0.00	0.19	1.75
9	0	Less mild/Asymptomatic PTSD	16.41%	$445	$0	$3,572	$35,762	0.04	0.19	1.93
9	1	Mild PTSD treatment	2.53%	$69	-$5	$3,572	$35,762	0.01	0.19	1.93
9	2	Moderate PTSD treatment	0.00%	$0	$0	$3,572	$35,762	0.00	0.19	1.93
9	3	Severe PTSD treatment	0.00%	$0	$0	$3,572	$35,762	0.00	0.19	1.93
9	4	Dead	1.59%	$0	$0	$3,572	$35,762	0.00	0.19	1.93
9	5	Mild PTSD	40.07%	$1,294	$0	$3,572	$35,762	0.08	0.19	1.93
9	6	Moderate PTSD	21.65%	$992	$0	$3,572	$35,762	0.04	0.19	1.93
9	7	Severe PTSD	14.30%	$777	$0	$3,572	$35,762	0.02	0.19	1.93
9	8	Relapse mild PTSD	3.45%	$0	$0	$3,572	$35,762	0.00	0.19	1.93
9	9	Relapse moderate PTSD	0.00%	$0	$0	$3,572	$35,762	0.00	0.19	1.93
9	10	Relapse severe PTSD	0.00%	$0	$0	$3,572	$35,762	0.00	0.19	1.93
10	0	Less mild/Asymptomatic PTSD	14.93%	$402	$0	$3,557	$39,319	0.03	0.19	2.12
10	1	Mild PTSD treatment	2.30%	$62	-$5	$3,557	$39,319	0.01	0.19	2.12

STAGE	STATE INDEX	STATE	PROBABILITY	STATE COST	TRANS COST	STAGE COST	TOTAL COST	STATE EFFECTIVENESS	STAGE EFFECTIVENESS	TOTAL EFFECTIVENESS
10	2	Moderate PTSD treatment	0.00%	$0	$0	$3,557	$39,319	0.00	0.19	2.12
10	3	Severe PTSD treatment	0.00%	$0	$0	$3,557	$39,319	0.00	0.19	2.12
10	4	Dead	1.77%	$0	$0	$3,557	$39,319	0.00	0.19	2.12
10	5	Mild PTSD	41.98%	$1,345	$0	$3,557	$39,319	0.09	0.19	2.12
10	6	Moderate PTSD	21.60%	$983	$0	$3,557	$39,319	0.04	0.19	2.12
10	7	Severe PTSD	14.26%	$769	$0	$3,557	$39,319	0.02	0.19	2.12
10	8	Relapse mild PTSD	3.14%	$0	$0	$3,557	$39,319	0.00	0.19	2.12
10	9	Relapse moderate PTSD	0.00%	$0	$0	$3,557	$39,319	0.00	0.19	2.12
10	10	Relapse severe PTSD	0.00%	$0	$0	$3,557	$39,319	0.00	0.19	2.12
11	0	Less mild/Asymptomatic PTSD	13.60%	$364	$0	$3,541	$42,860	0.03	0.18	2.30
11	1	Mild PTSD treatment	2.10%	$56	-$4	$3,541	$42,860	0.00	0.18	2.30
11	2	Moderate PTSD treatment	0.00%	$0	$0	$3,541	$42,860	0.00	0.18	2.30
11	3	Severe PTSD treatment	0.00%	$0	$0	$3,541	$42,860	0.00	0.18	2.30
11	4	Dead	1.95%	$0	$0	$3,541	$42,860	0.00	0.18	2.30
11	5	Mild PTSD	43.71%	$1,390	$0	$3,541	$42,860	0.09	0.18	2.30
11	6	Moderate PTSD	21.55%	$974	$0	$3,541	$42,860	0.04	0.18	2.30
11	7	Severe PTSD	14.23%	$762	$0	$3,541	$42,860	0.02	0.18	2.30
11	8	Relapse mild PTSD	2.86%	$0	$0	$3,541	$42,860	0.00	0.18	2.30
11	9	Relapse moderate PTSD	0.00%	$0	$0	$3,541	$42,860	0.00	0.18	2.30
11	10	Relapse severe PTSD	0.00%	$0	$0	$3,541	$42,860	0.00	0.18	2.30
12	0	Less mild/Asymptomatic PTSD	12.38%	$329	$0	$3,527	$46,388	0.03	0.18	2.49
12	1	Mild PTSD treatment	1.91%	$51	$0	$3,527	$46,388	0.00	0.18	2.49
12	2	Moderate PTSD treatment	0.00%	$0	$0	$3,527	$46,388	0.00	0.18	2.49
12	3	Severe PTSD treatment	0.00%	$0	$0	$3,527	$46,388	0.00	0.18	2.49
12	4	Dead	2.14%	$0	$0	$3,527	$46,388	0.00	0.18	2.49
12	5	Mild PTSD	45.28%	$1,430	$0	$3,527	$46,388	0.09	0.18	2.49
12	6	Moderate PTSD	21.51%	$964	$0	$3,527	$46,388	0.04	0.18	2.49
12	7	Severe PTSD	14.19%	$754	$0	$3,527	$46,388	0.02	0.18	2.49
12	8	Relapse mild PTSD	2.60%	$0	$0	$3,527	$46,388	0.00	0.18	2.49

STAGE	STATE INDEX	STATE	PROBABILITY	STATE COST	TRANS COST	STAGE COST	TOTAL COST	STATE EFFECTIVENESS	STAGE EFFECTIVENESS	TOTAL EFFECTIVENESS
12	9	Relapse moderate PTSD	0.00%	$0	$0	$3,527	$46,388	0.00	0.18	2.49
12	10	Relapse severe PTSD	0.00%	$0	$0	$3,527	$46,388	0.00	0.18	2.49

NF+OT

STAGE	STATE INDEX	STATE	PROBABILITY	STATE COST	TRANS COST	STAGE COST	TOTAL COST	STATE EFFECTIVENESS	STAGE EFFECTIVENESS	TOTAL EFFECTIVENESS
0	0	Less mild/Asympotmatic PTSD	0.00%	$0	$0	$3,627	$3,627	0.00	0.22	0.22
0	1	Mild PTSD treatment	30.20%	$854	-$111	$3,627	$3,627	0.07	0.22	0.22
0	2	Moderate PTSD treatment	33.20%	$1,241	-$122	$3,627	$3,627	0.07	0.22	0.22
0	3	Severe PTSD treatment	36.60%	$1,899	-$135	$3,627	$3,627	0.07	0.22	0.22
0	4	Dead	0.00%	$0	$0	$3,627	$3,627	0.00	0.22	0.22
0	5	Mild PTSD	0.00%	$0	$0	$3,627	$3,627	0.00	0.22	0.22
0	6	Moderate PTSD	0.00%	$0	$0	$3,627	$3,627	0.00	0.22	0.22
0	7	Severe PTSD	0.00%	$0	$0	$3,627	$3,627	0.00	0.22	0.22
0	8	Relapse mild PTSD	0.00%	$0	$0	$3,627	$3,627	0.00	0.22	0.22
0	9	Relapse moderate PTSD	0.00%	$0	$0	$3,627	$3,627	0.00	0.22	0.22
0	10	Relapse severe PTSD	0.00%	$0	$0	$3,627	$3,627	0.00	0.22	0.22
1	0	Less mild/Asympotmatic PTSD	25.34%	$695	$0	$2,965	$6,592	0.06	0.22	0.43
1	1	Mild PTSD treatment	27.83%	$752	-$102	$2,965	$6,592	0.07	0.22	0.43
1	2	Moderate PTSD treatment	30.68%	$1,106	-$112	$2,965	$6,592	0.07	0.22	0.43
1	3	Severe PTSD treatment	0.00%	$0	$0	$2,965	$6,592	0.00	0.22	0.43
1	4	Dead	0.17%	$0	$0	$2,965	$6,592	0.00	0.22	0.43
1	5	Mild PTSD	3.98%	$136	$0	$2,965	$6,592	0.01	0.22	0.43
1	6	Moderate PTSD	4.37%	$213	$0	$2,965	$6,592	0.01	0.22	0.43
1	7	Severe PTSD	4.82%	$278	$0	$2,965	$6,592	0.01	0.22	0.43
1	8	Relapse mild PTSD	0.85%	$0	$0	$2,965	$6,592	0.00	0.22	0.43
1	9	Relapse moderate PTSD	0.93%	$0	$0	$2,965	$6,592	0.00	0.22	0.43

1	10	Relapse severe PTSD	1.03%	$0	$0	$2,965	$6,592	0.00	0.22	0.43
2	0	Less mild/Asympotmatic PTSD	47.83%	$1,302	$0	$2,948	$9,540	0.11	0.22	0.65
2	1	Mild PTSD treatment	26.28%	$705	-$95	$2,948	$9,540	0.06	0.22	0.65
2	2	Moderate PTSD treatment	0.62%	$22	-$2	$2,948	$9,540	0.00	0.22	0.65
2	3	Severe PTSD treatment	0.69%	$34	-$2	$2,948	$9,540	0.00	0.22	0.65
2	4	Dead	0.32%	$0	$0	$2,948	$9,540	0.00	0.22	0.65
2	5	Mild PTSD	7.92%	$269	$0	$2,948	$9,540	0.02	0.22	0.65
2	6	Moderate PTSD	8.72%	$421	$0	$2,948	$9,540	0.02	0.22	0.65
2	7	Severe PTSD	5.15%	$295	$0	$2,948	$9,540	0.01	0.22	0.65
2	8	Relapse mild PTSD	1.61%	$0	$0	$2,948	$9,540	0.00	0.22	0.65
2	9	Relapse moderate PTSD	0.86%	$0	$0	$2,948	$9,540	0.00	0.22	0.65
2	10	Relapse severe PTSD	0.00%	$0	$0	$2,948	$9,540	0.00	0.22	0.65
3	0	Less mild/Asympotmatic PTSD	68.28%	$1,845	$0	$3,051	$12,591	0.16	0.22	0.87
3	1	Mild PTSD treatment	1.59%	$42	-$6	$3,051	$12,591	0.00	0.22	0.87
3	2	Moderate PTSD treatment	1.15%	$41	-$4	$3,051	$12,591	0.00	0.22	0.87
3	3	Severe PTSD treatment	0.00%	$0	$0	$3,051	$12,591	0.00	0.22	0.87
3	4	Dead	0.45%	$0	$0	$3,051	$12,591	0.00	0.22	0.87
3	5	Mild PTSD	11.91%	$402	$0	$3,051	$12,591	0.03	0.22	0.87
3	6	Moderate PTSD	9.07%	$435	$0	$3,051	$12,591	0.02	0.22	0.87
3	7	Severe PTSD	5.23%	$297	$0	$3,051	$12,591	0.01	0.22	0.87
3	8	Relapse mild PTSD	2.29%	$0	$0	$3,051	$12,591	0.00	0.22	0.87
3	9	Relapse moderate PTSD	0.02%	$0	$0	$3,051	$12,591	0.00	0.22	0.87
3	10	Relapse severe PTSD	0.02%	$0	$0	$3,051	$12,591	0.00	0.22	0.87
4	0	Less mild/Asympotmatic PTSD	67.33%	$1,806	$0	$3,026	$15,617	0.16	0.21	1.08
4	1	Mild PTSD treatment	2.49%	$66	-$9	$3,026	$15,617	0.01	0.21	1.08
4	2	Moderate PTSD treatment	0.01%	$0	$0	$3,026	$15,617	0.00	0.21	1.08
4	3	Severe PTSD treatment	0.01%	$1	$0	$3,026	$15,617	0.00	0.21	1.08
4	4	Dead	0.58%	$0	$0	$3,026	$15,617	0.00	0.21	1.08
4	5	Mild PTSD	12.86%	$431	$0	$3,026	$15,617	0.03	0.21	1.08
4	6	Moderate PTSD	9.21%	$438	$0	$3,026	$15,617	0.02	0.21	1.08
4	7	Severe PTSD	5.22%	$294	$0	$3,026	$15,617	0.01	0.21	1.08

4	8	Relapse mild PTSD	2.26%	$0	$0	$3,026	$15,617	0.00	0.21	1.08
4	9	Relapse moderate PTSD	0.03%	$0	$0	$3,026	$15,617	0.00	0.21	1.08
4	10	Relapse severe PTSD	0.00%	$0	$0	$3,026	$15,617	0.00	0.21	1.08
5	0	Less mild/Asympotmatic PTSD	67.16%	$1,788	$0	$3,012	$18,629	0.15	0.21	1.30
5	1	Mild PTSD treatment	1.52%	$40	-$5	$3,012	$18,629	0.00	0.21	1.30
5	2	Moderate PTSD treatment	0.03%	$1	$0	$3,012	$18,629	0.00	0.21	1.30
5	3	Severe PTSD treatment	0.00%	$0	$0	$3,012	$18,629	0.00	0.21	1.30
5	4	Dead	0.71%	$0	$0	$3,012	$18,629	0.00	0.21	1.30
5	5	Mild PTSD	13.92%	$463	$0	$3,012	$18,629	0.03	0.21	1.30
5	6	Moderate PTSD	9.20%	$434	$0	$3,012	$18,629	0.02	0.21	1.30
5	7	Severe PTSD	5.21%	$291	$0	$3,012	$18,629	0.01	0.21	1.30
5	8	Relapse mild PTSD	2.25%	$0	$0	$3,012	$18,629	0.00	0.21	1.30
5	9	Relapse moderate PTSD	0.00%	$0	$0	$3,012	$18,629	0.00	0.21	1.30
5	10	Relapse severe PTSD	0.00%	$0	$0	$3,012	$18,629	0.00	0.21	1.30
6	0	Less mild/Asympotmatic PTSD	66.18%	$1,749	$0	$2,992	$21,621	0.15	0.21	1.51
6	1	Mild PTSD treatment	1.53%	$40	-$5	$2,992	$21,621	0.00	0.21	1.51
6	2	Moderate PTSD treatment	0.00%	$0	$0	$2,992	$21,621	0.00	0.21	1.51
6	3	Severe PTSD treatment	0.00%	$0	$0	$2,992	$21,621	0.00	0.21	1.51
6	4	Dead	0.85%	$0	$0	$2,992	$21,621	0.00	0.21	1.51
6	5	Mild PTSD	14.84%	$490	$0	$2,992	$21,621	0.03	0.21	1.51
6	6	Moderate PTSD	9.18%	$430	$0	$2,992	$21,621	0.02	0.21	1.51
6	7	Severe PTSD	5.19%	$288	$0	$2,992	$21,621	0.01	0.21	1.51
6	8	Relapse mild PTSD	2.22%	$0	$0	$2,992	$21,621	0.00	0.21	1.51
6	9	Relapse moderate PTSD	0.00%	$0	$0	$2,992	$21,621	0.00	0.21	1.51
6	10	Relapse severe PTSD	0.00%	$0	$0	$2,992	$21,621	0.00	0.21	1.51
7	0	Less mild/Asympotmatic PTSD	65.25%	$1,711	$0	$2,973	$24,594	0.15	0.21	1.72
7	1	Mild PTSD treatment	1.48%	$38	-$5	$2,973	$24,594	0.00	0.21	1.72
7	2	Moderate PTSD treatment	0.00%	$0	$0	$2,973	$24,594	0.00	0.21	1.72
7	3	Severe PTSD treatment	0.00%	$0	$0	$2,973	$24,594	0.00	0.21	1.72
7	4	Dead	0.98%	$0	$0	$2,973	$24,594	0.00	0.21	1.72
7	5	Mild PTSD	15.76%	$516	$0	$2,973	$24,594	0.03	0.21	1.72

7	6	Moderate PTSD	9.16%	$426	$0	$2,973	$24,594	0.02	0.21	1.72
7	7	Severe PTSD	5.18%	$286	$0	$2,973	$24,594	0.01	0.21	1.72
7	8	Relapse mild PTSD	2.19%	$0	$0	$2,973	$24,594	0.00	0.21	1.72
7	9	Relapse moderate PTSD	0.00%	$0	$0	$2,973	$24,594	0.00	0.21	1.72
7	10	Relapse severe PTSD	0.00%	$0	$0	$2,973	$24,594	0.00	0.21	1.72
8	0	Less mild/Asympotmatic PTSD	64.30%	$1,674	$0	$2,953	$27,547	0.14	0.21	1.92
8	1	Mild PTSD treatment	1.46%	$37	-$5	$2,953	$27,547	0.00	0.21	1.92
8	2	Moderate PTSD treatment	0.00%	$0	$0	$2,953	$27,547	0.00	0.21	1.92
8	3	Severe PTSD treatment	0.00%	$0	$0	$2,953	$27,547	0.00	0.21	1.92
8	4	Dead	1.11%	$0	$0	$2,953	$27,547	0.00	0.21	1.92
8	5	Mild PTSD	16.65%	$542	$0	$2,953	$27,547	0.03	0.21	1.92
8	6	Moderate PTSD	9.14%	$422	$0	$2,953	$27,547	0.02	0.21	1.92
8	7	Severe PTSD	5.17%	$283	$0	$2,953	$27,547	0.01	0.21	1.92
8	8	Relapse mild PTSD	2.16%	$0	$0	$2,953	$27,547	0.00	0.21	1.92
8	9	Relapse moderate PTSD	0.00%	$0	$0	$2,953	$27,547	0.00	0.21	1.92
8	10	Relapse severe PTSD	0.00%	$0	$0	$2,953	$27,547	0.00	0.21	1.92
9	0	Less mild/Asympotmatic PTSD	63.37%	$1,638	$0	$2,934	$30,481	0.14	0.21	2.13
9	1	Mild PTSD treatment	1.44%	$37	-$5	$2,934	$30,481	0.00	0.21	2.13
9	2	Moderate PTSD treatment	0.00%	$0	$0	$2,934	$30,481	0.00	0.21	2.13
9	3	Severe PTSD treatment	0.00%	$0	$0	$2,934	$30,481	0.00	0.21	2.13
9	4	Dead	1.25%	$0	$0	$2,934	$30,481	0.00	0.21	2.13
9	5	Mild PTSD	17.54%	$566	$0	$2,934	$30,481	0.04	0.21	2.13
9	6	Moderate PTSD	9.12%	$418	$0	$2,934	$30,481	0.02	0.21	2.13
9	7	Severe PTSD	5.15%	$280	$0	$2,934	$30,481	0.01	0.21	2.13
9	8	Relapse mild PTSD	2.13%	$0	$0	$2,934	$30,481	0.00	0.21	2.13
9	9	Relapse moderate PTSD	0.00%	$0	$0	$2,934	$30,481	0.00	0.21	2.13
9	10	Relapse severe PTSD	0.00%	$0	$0	$2,934	$30,481	0.00	0.21	2.13
10	0	Less mild/Asympotmatic PTSD	62.46%	$1,602	$0	$2,914	$33,395	0.14	0.20	2.33
10	1	Mild PTSD treatment	1.42%	$36	-$5	$2,914	$33,395	0.00	0.20	2.33
10	2	Moderate PTSD treatment	0.00%	$0	$0	$2,914	$33,395	0.00	0.20	2.33
10	3	Severe PTSD treatment	0.00%	$0	$0	$2,914	$33,395	0.00	0.20	2.33

10	4	Dead	1.38%	$0	$0	$2,914	$33,395	0.00	0.20	2.33
10	5	Mild PTSD	18.40%	$590	$0	$2,914	$33,395	0.04	0.20	2.33
10	6	Moderate PTSD	9.10%	$414	$0	$2,914	$33,395	0.02	0.20	2.33
10	7	Severe PTSD	5.14%	$277	$0	$2,914	$33,395	0.01	0.20	2.33
10	8	Relapse mild PTSD	2.10%	$0	$0	$2,914	$33,395	0.00	0.20	2.33
10	9	Relapse moderate PTSD	0.00%	$0	$0	$2,914	$33,395	0.00	0.20	2.33
10	10	Relapse severe PTSD	0.00%	$0	$0	$2,914	$33,395	0.00	0.20	2.33
11	0	Less mild/Asympotmatic PTSD	61.55%	$1,568	$0	$2,895	$36,290	0.13	0.20	2.53
11	1	Mild PTSD treatment	1.40%	$35	-$5	$2,895	$36,290	0.00	0.20	2.53
11	2	Moderate PTSD treatment	0.00%	$0	$0	$2,895	$36,290	0.00	0.20	2.53
11	3	Severe PTSD treatment	0.00%	$0	$0	$2,895	$36,290	0.00	0.20	2.53
11	4	Dead	1.52%	$0	$0	$2,895	$36,290	0.00	0.20	2.53
11	5	Mild PTSD	19.25%	$612	$0	$2,895	$36,290	0.04	0.20	2.53
11	6	Moderate PTSD	9.08%	$410	$0	$2,895	$36,290	0.02	0.20	2.53
11	7	Severe PTSD	5.12%	$274	$0	$2,895	$36,290	0.01	0.20	2.53
11	8	Relapse mild PTSD	2.07%	$0	$0	$2,895	$36,290	0.00	0.20	2.53
11	9	Relapse moderate PTSD	0.00%	$0	$0	$2,895	$36,290	0.00	0.20	2.53
11	10	Relapse severe PTSD	0.00%	$0	$0	$2,895	$36,290	0.00	0.20	2.53
12	0	Less mild/Asympotmatic PTSD	60.66%	$1,533	$0	$2,880	$39,171	0.13	0.20	2.73
12	1	Mild PTSD treatment	1.38%	$34	$0	$2,880	$39,171	0.00	0.20	2.73
12	2	Moderate PTSD treatment	0.00%	$0	$0	$2,880	$39,171	0.00	0.20	2.73
12	3	Severe PTSD treatment	0.00%	$0	$0	$2,880	$39,171	0.00	0.20	2.73
12	4	Dead	1.65%	$0	$0	$2,880	$39,171	0.00	0.20	2.73
12	5	Mild PTSD	20.09%	$634	$0	$2,880	$39,171	0.04	0.20	2.73
12	6	Moderate PTSD	9.06%	$406	$0	$2,880	$39,171	0.02	0.20	2.73
12	7	Severe PTSD	5.11%	$272	$0	$2,880	$39,171	0.01	0.20	2.73
12	8	Relapse mild PTSD	2.04%	$0	$0	$2,880	$39,171	0.00	0.20	2.73
12	9	Relapse moderate PTSD	0.00%	$0	$0	$2,880	$39,171	0.00	0.20	2.73
12	10	Relapse severe PTSD	0.00%	$0	$0	$2,880	$39,171	0.00	0.20	2.73
